# Increase of enzyme activity through specific covalent modification with fragments†
†Electronic supplementary information (ESI) available: Description of the experimental methods, chemical synthesis and analysis, details of the results and supplementary figures and tables. See DOI: 10.1039/c7sc01966a



**DOI:** 10.1039/c7sc01966a

**Published:** 2017-09-27

**Authors:** John F. Darby, Masakazu Atobe, James D. Firth, Paul Bond, Gideon J. Davies, Peter O'Brien, Roderick E. Hubbard

**Affiliations:** a Department of Chemistry , University of York , Heslington , York , YO10 5DD , UK . Email: roderick.hubbard@york.ac.uk; b Asahi Kasei Pharma Corporation , 632-1 Mifuku, Izunokuni , Shizuoka 410-2321 , Japan; c Vernalis (R&D) Ltd , Granta Park, Abington , Cambridge , CB21 6GB , UK

## Abstract

Modulation of enzyme activity is a powerful means of probing cellular function and can be exploited for diverse applications. Here, we explore a method of enzyme activation where covalent tethering of a small molecule to an enzyme can increase catalytic activity (*k*_cat_/*K*_M_) up to 35-fold. Using a bacterial glycoside hydrolase, BtGH84, we demonstrate how small molecule “fragments”, identified as activators in free solution, can be covalently tethered to the protein using Michael-addition chemistry. We show how tethering generates a constitutively-activated enzyme-fragment conjugate, which displays both improved catalytic efficiency and increased susceptibility to certain inhibitor classes. Structure guided modifications of the tethered fragment demonstrate how specific interactions between the fragment and the enzyme influence the extent of activation. This work suggests that a similar approach may be used to modulate the activity of enzymes such as to improve catalytic efficiency or increase inhibitor susceptibility.

## Abstract 2

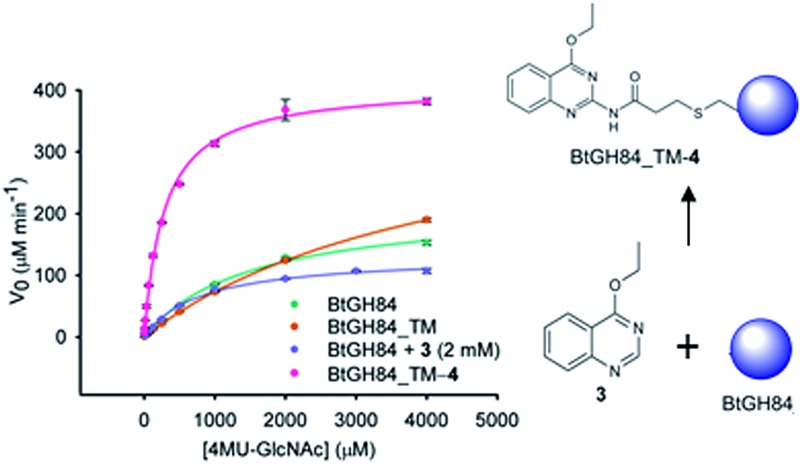
Structure-guided tethering of a fragment activator significantly increases enzyme activity.

## Introduction

In nature, direct binding of an enzyme to another molecule to increase catalytic activity has evolved as a control mechanism in many biological systems; either through binding of another protein molecule (such as cyclin binding to cyclin-dependent kinases,^1^ co-chaperones binding to Hsp90 ^2^ and GAPs binding to K-Ras^3^) or less frequently through binding of an endogenous small molecule (such as nicotinamide binding to sirtuins^4^ and AMP binding to AMP-activated kinase^5^). There have, however, been relatively few examples where such activation mechanisms have been exploited “artificially” using small molecules;^6^ notable exceptions being activators of SIRT1,^7^ glucokinase^8^ and ALDH2.^9^ For those artificial systems where mechanistic insight has been obtained, the activators manipulate catalysis through affecting allosteric regulation,^8^ conformational change,^10^ enzyme dynamics^11,12^ or substrate binding.^9^ Enzyme activation with small molecules *via* these mechanisms often requires that the activator be present in excess of the enzyme concentration. There are some studies which demonstrate that this limitation can be overcome through covalent modification. Work from nearly 20 years ago on subtilisin *Bacillus lentus* demonstrated activation from chemical modification of a cysteine introduced in the substrate binding site.^13,14^ A different strategy is to screen for disulphide containing compounds that react with natural or introduced cysteine residues,^15^ a tethering approach which has in one instance led to increase in activity of a kinase.^16^ Although these studies demonstrate that covalent attachment of a synthetic molecule to an enzyme can lead to increased activity, they depend on prior knowledge of where to introduce the covalent ligand. In the present work we demonstrate a rational approach to designing such covalent modifications through structure-guided incorporation of small molecule activators at a site identified from fragment screening.

We have used a glycoside hydrolase from *Bacteroides thetaiotaomicron*, BtGH84,^17^ as a model system to explore activation by small molecules. The catalytic domain of BtGH84 is homologous to that of the human enzyme O-GlcNAcase (OGA) which removes the *N*-acetylglucosamine post-translational modification on serine and threonine amino acids^18^ and BtGH84 itself indeed functions as a generic hexosaminidase.^19^ BtGH84 has been a useful model for analysis of compounds such as PUGNAc (**1**)^20^ and thiamet-G (**2**),^21^ and the utility of this model has recently been confirmed by publication of the first hOGA crystal structures.^22–24^ These compounds modify the O-GlcNAc status in cells, with thiamet-G demonstrating impact on a variety of biological processes and with therapeutic potential, particularly for neurodegeneration.^25–27^ Previously,^28^ we described the unusual activation of BtGH84 by small molecules. We used ligand-observed NMR spectroscopy to identify small organic molecules (fragments) which bound to BtGH84. Most were inhibitory and competitive with PUGNAc but some, such as **3**, were non-competitive. Furthermore, **3** not only enhanced the binding of PUGNAc, but also increased the catalytic activity of the enzyme through a non-essential activator mechanism.^29^ Subsequent optimisation and characterisation of the fragments (including a crystal structure – PDB code: ; 4UR9) demonstrated that the fragments bind close to the active site and appear to stabilise an active “loop-closed” conformation.

As with BtGH84, a large number of enzymes perform catalysis with mechanisms that exploit conformational changes.^30^ Fragment-based activator discovery could therefore be an approach to identify compounds that affect conformational change, to probe the biological role of an enzyme in the cell, as a potential therapeutic agent (as with glucokinase^31^) but also to increase the activity of enzymes used in biotechnological or industrial processes. Although there is extensive work in optimising conditions for the activity of industrial enzymes (*e.g.* pH, solvents, and immobilisation as reviewed in ref. 33 and 34), there have not been reports on identifying activators. Such an approach using non-covalent compounds may indeed not be commercially feasible, due to the cost of the activator compound required, which would be expensive to recover or separate from the products of the catalytic process – analogous to some of the challenges surrounding cofactor-dependent industrial biocatalysts.^34^ However, chemical attachment of the activator to the enzyme could give increased activation and circumvent the issues of activator recovery and separation. This would provide an alternate strategy to current techniques, such as directed evolution, to engineer improvements in the performance of an industrial enzyme.

Here, we validate the covalent-activation strategy using a tethering approach established for irreversible inhibition.^35^ We describe the design, synthesis and characterisation of fragment activators modified to attach covalently to a specific attachment site (cysteine) introduced into BtGH84 and show that specific interactions made by the small molecule affect the catalytic activity. This model system demonstrates that tethering of a fragment can lead to a modified enzyme with significantly enhanced activity. The work provides the foundation for a combined fragment screening and tethering approach as a general strategy to be considered for enzyme optimisation in future. This strategy may find use in the activation of enzymes used for industrial processes or to enhance the effects of pharmacological small-molecule activators.

## Results

### Design and synthesis of tethering compounds and mutant BtGH84

**Fig. 1:**
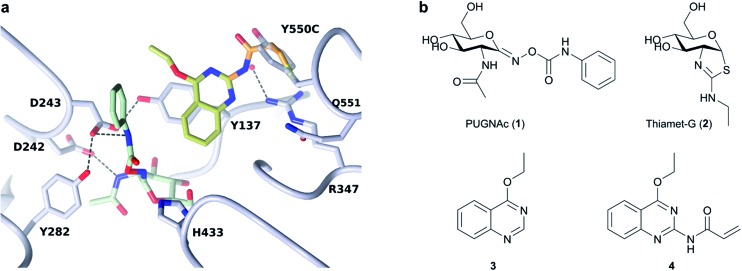
Tether design and chemical structures: (a) Structure of BtGH84 (grey) in complex with **3** (yellow) and PUGNAc (green) from PDB:; 4UR9. Model of designed acrylamide linker is shown in orange, demonstrating the possibility of linking fragment **3** to an introduced cysteine at the position of Y550. (b) Chemical structures for the BtGH84 inhibitors PUGNAc (**1**) and thiamet-G (**2**), activator **3** and tethering compound **4**.

Previous work had identified small molecule activators of BtGH84,^25^ including activator **3**. Here, we have designed fragment tethers using the previously solved crystal structure of **3** and PUGNAc (**1**) bound to BtGH84 (see Fig. 1, PDB code: ; 4UR9), in order to conjugate the activators to the enzyme. We noted that position 2 on the quinazoline ring of **3** presented a vector towards Cγ of Y550, positioned on a flexible loop above the enzyme active site. We designed tethering compound **4**, which we predicted would undergo Michael-style conjugate addition to the acrylamide from the free cysteine thiol introduced by the mutation Y550C. Modelling of the resulting linker showed that the tethered quinazoline ring should be able to occupy the position observed in the crystal structure without significant linker strain. In order to prevent promiscuous off-site reactivity, we analysed the positions of the three other free cysteine residues in BtGH84. C420 and C654 are solvent exposed whilst the third, C278, is buried close to the binding site and is likely inaccessible to modification. In order to direct reactivity of **4** with BtGH84 to the desired site, a triple mutant (Y550C, C420S, C654S, hereafter BtGH84_TM – signifying triple mutant) was expressed. Enzymatic activity of this variant was reduced, with the *k*_cat_/*K*_M_ lowered to 60% of that of wild-type BtGH84 (Table 1).

**Table 1:** Kinetic and inhibitor binding data for BtGH84, mutant BtGH84_TM and covalently modified BtGH84_TM

Protein		BtGH84	BtGH84_TM							
Modification		None	None	**4**	**5**	**6**	**7**	**8**	**9**	**10**
*V*_max_	μM min^–1^	200 ± 14	380 ± 20	415 ± 9	69 ± 2	510 ± 60	250 ± 6	320 ± 60	305 ± 85	320 ± 35
*K*_M_	μM	1460 ± 220	4680 ± 220	280 ± 10	370 ± 70	190 ± 2	400 ± 25	200 ± 10	145 ± 18	230 ± 46
*k*_cat_	s^–1^	67 ± 5	127 ± 6	138 ± 3	23 ± 1	171 ± 21	82 ± 2	108 ± 19	102 ± 28	107 ± 12
*k*_cat_/*K*_M_	M^–1^ sec^–1^	46 000 ± 5900	27 200 ± 2460	498 200 ± 29 400	64 200 ± 10 900	888 000 ± 99 000	207 000 ± 19 000	541 000 ± 70 100	694 000 ± 110 000	471 000 ± 143 000
Fold	WT	1.0	0.6 ± 0.05	10.8 ± 0.6	1.4 ± 0.2	19.3 ± 2.1	4.5 ± 0.4	11.7 ± 1.5	15.1 ± 2.4	10.2 ± 3.1
Fold	Parent	n/a	n/a	18.3 ± 1.1	2.4 ± 0.4	32.6 ± 3.6	7.6 ± 0.7	19.9 ± 2.6	25.5 ± 4.0	17.3 ± 5.3
*K*_d_ (PUGNAc)	nM	2500 ± 200	2600 ± 110	170 ± 10	830 ± 530	520 ± 200	2550 ± 580	77 ± 7.7	780 ± 135	ND
*K*_d_ (thiamet-G)	nM	50	52 ± 5.7	50 ± 7.6	45 ± 16	51 ± 10	73 ± 21	44 ± 15	59 ± 27	75 ± 16

The designed covalent tether was attached to BtGH84 in aqueous solution by combining BtGH84_TM with an excess of **4** (synthesised *via* the Curtius rearrangement as shown in ESI Scheme S1†). This reaction resulted in complete conversion to the conjugate product, BtGH84_TM-**4**, within 2–4 hours. This conversion was confirmed by intact protein ESI mass spectrometry (ESI†) which showed a species of the expected molecular weight for BtGH84_TM-**4**, with no BtGH84_TM observed in the sample. To corroborate this the product was further characterised using Ellman's reagent^32^ (5,5′-dithiobis-(2-nitrobenzoic acid) or DTNB), an accurate colorimetric method to quantify free thiol concentration present in a sample. BtGH84_TM-**4** was denatured and the thiol concentration shown to be equivalent to one thiol per molecule of BtGH84_TM-**4**, whereas unlabelled BtGH84_TM contained two thiols per molecule (ESI Fig. 1†). These results demonstrate complete conversion to BtGH84_TM-**4** at the desired site since any reactivity at the buried C278 should show a mixture of mono- and di-substituted product evident in either the mass spectrometry or thiol quantification data.

**Fig. 2:**
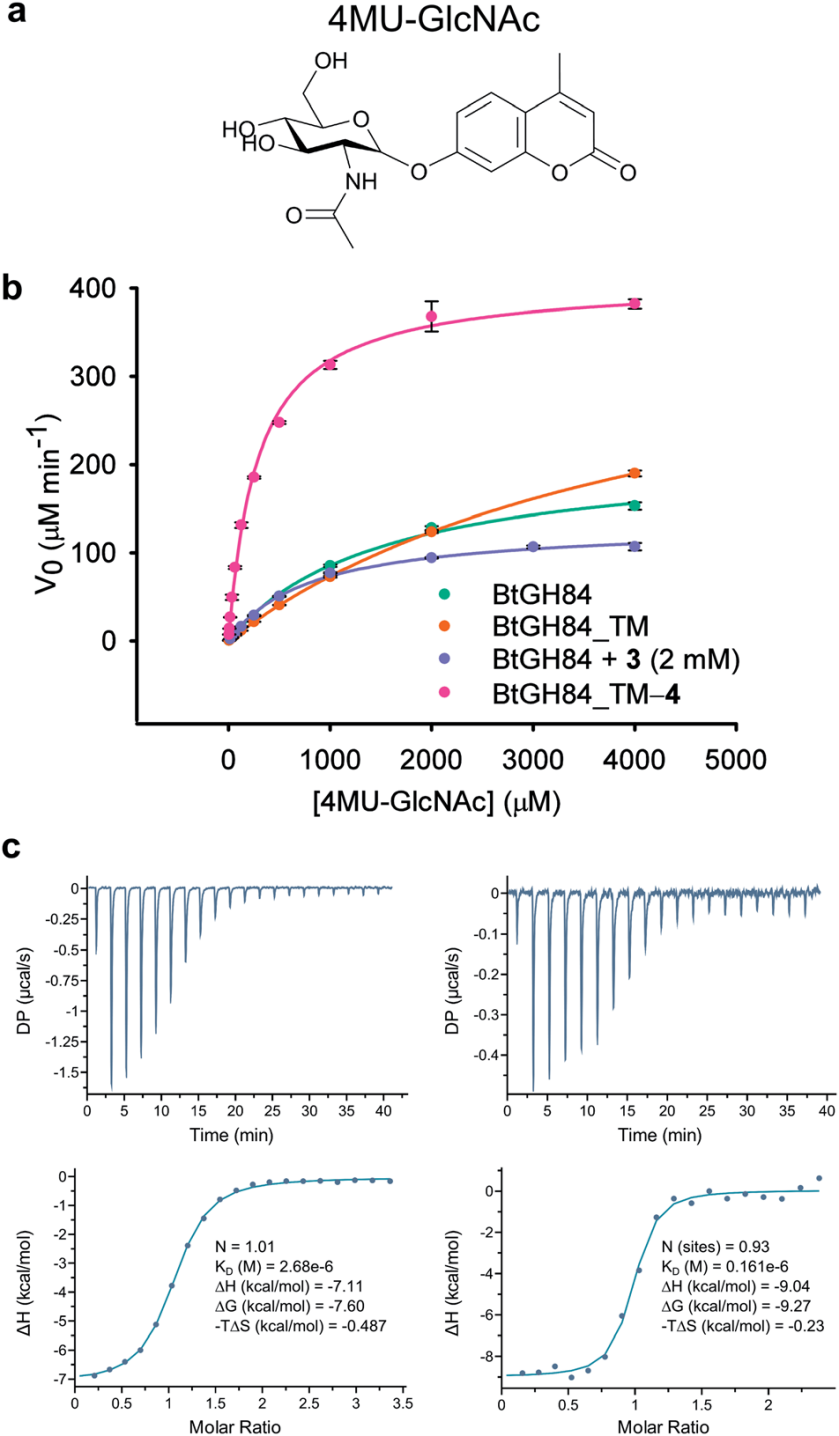
Activity and inhibitor binding of BtGH84_TM-**4** conjugate: (a) structure of the 4MU-GlcNAc substrate used in this study. (b) Michaelis–Menten plot for the hydrolysis of 4MU-GlcNAc by BtGH84_TM-**4** (pink), in comparison to native enzyme (green), unmodified BtGH84 (orange) and the non-covalent activator **3** (purple). (c) Representative ITC data for PUGNAc binding to BtGH84_TM (left) and BtGH84_TM-**4** (right).

In order to assess the impact of the tether on catalytic efficiency, the activity of the resultant BtGH84_TM-**4** conjugate was determined by conversion of the synthetic substrate 4-methylumbelliferyl *N*-acetyl-β-d-glucosaminide (4MU-GlcNAc, Fig. 2a) to the fluorescent 4-methylumbelliferone (4MU) product. As shown through Michaelis–Menten kinetics, Fig. 2b, there is a dramatic change in activity both in terms of the Michaelis constant (*K*_M_) and maximum velocity (*V*_max_) of the covalently modified enzyme BtGH84_TM-**4** towards this substrate. Using the *k*_cat_/*K*_M_ as a quantification of enzyme activity, BtGH84_TM-**4** demonstrated a 10-fold increase over the activity of wild-type BtGH84 and an 18-fold increase over the parent BtGH84_TM enzyme, Table 1.

We hypothesized that covalent tethering might also increase the affinity for inhibitors with chemical similarity to the substrate used (which would also further increase the utility of the tethering approach for exploring biological processes). The BtGH84 inhibitor PUGNAc (**1**) contains a hydrophobic phenyl ring linked to *N*-acetyl-β-d-glucosaminide, similar to the structure of the 4MU-GlcNAc substrate – a hydrophobic aglycone linked to the protein binding sugar (ESI Fig. 8†). We therefore assessed the binding of PUGNAc in the presence and absence of the covalent tether using isothermal titration calorimetry (ITC). The BtGH84_TM-**4** conjugate binds PUGNAc with a *K*_d_ of 0.17 μM; a more than 10-fold increase in affinity from BtGH84_TM, Fig. 2c and d, showing that the presence of the tether does indeed increase the binding affinity of PUGNAc.

In order to understand the mode of action and binding, X-ray crystal structures of the tethered BtGH84_TM-**4**, and structures of BtGH84_TM-**4** with the active site occupied by inhibitors thiamet-G (**2**) and PUGNAc (**1**), were determined by X-ray crystallography at resolutions of 1.8, 2.0 and 2.15 Å respectively, ESI Table 1.† The structure of BtGH84_TM-**4** in the absence of an inhibitor shows density for the tethered compound **4**, with the best model for this density positioning **4** rotated away from the active site, ESI Fig. 4,† compared to the position observed for the non-covalent activator **3** (ESI Fig. 5†). In the absence of an inhibitor in this structure the active site is occupied by ethylene glycol.

The structure of BtGH84_TM-**4** with thiamet-G, an inhibitor lacking a hydrophobic aglycone with which the quinazoline ring can interact, shows clear electron density for **4**, but again with some uncertainty over the precise position of the fragment, ESI Fig. 4 and 5.† The flexible linker apparently allows the fragment to occupy multiple conformations in these cases where there are no constraining interactions. In comparison to the apo structure, the best fitting position of the tether in the thiamet-G complex is flipped by 180°, but remains positioned away from the active site.

**Fig. 3:**
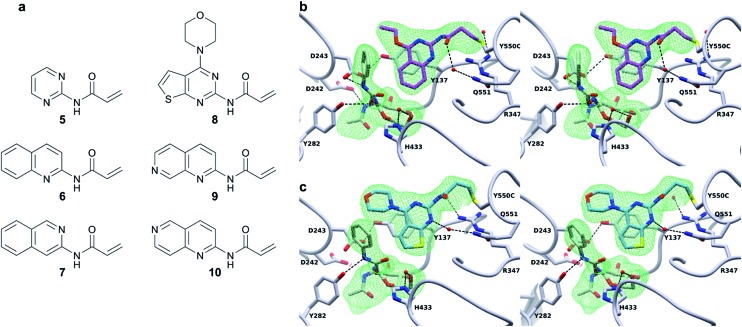
Covalent activator chemical structures and X-ray crystal structures of BtGH84_TM conjugates. (a) Chemical structures of the six additional acrylamide containing compounds used to label the BtGH84_TM mutant. (b) Stereo image of the BtGH84_TM-**4** conjugate (grey) X-ray crystal structure with PUGNAc (green carbons) bound to the active site. The covalent modification is highlighted (pink carbons) and the SA Fo-Fc omit map of PUGNAc and **4** is shown as green mesh contoured at 3.5*σ*. (c) Stereo image of the BtGH84_TM-**8** conjugate (grey) X-ray crystal structure with PUGNAc (green carbons) bound to the active site. The covalent modification is highlighted (blue carbons) and the SA Fo-Fc omit map of PUGNAc and **8** is shown as green mesh contoured at 3.5*σ*.

Excitingly, obtaining a structure of BtGH84_TM-**4** in complex with PUGNAc (**2**) shows the tether clearly in a position consistent with the model in Fig. 1 based on the non-covalent complex of BtGH84, **3** and PUGNAc. The presence of the inhibitor aglycone locks the flexible modification of **4** at Y550C into a single position and the electron density of the modification is well defined, Fig. 3b. The quinazoline ring is seen stacking onto Y137 and pointing towards the active site, with the amide linker coordinating a water molecule with Q551. The varying position of the tether in these three structures demonstrates the flexibility of the linker, however the fragment is in each case centred on the π-stacking interaction with Y137 (ESI Fig. 9†). This led us to consider how the specific interactions of the tether contribute towards the effects seen on the enzymatic activity and inhibitor binding of BtGH84.

### Investigating the chemistry of the covalent modification

In order to dissect the role of the specific chemistry of the BtGH84_TM-**4** conjugate in altering enzyme activity, we designed and synthesised a small family of analogues of **4** to interrogate specific interactions observed between the non-covalent activator **3** and BtGH84, and which are also seen in the BtGH84_TM-**4** PUGNAc-bound structure. The π-stacking of the activators **3** and **4** onto Y137 shown in Fig. 1 and 3b may promote formation of the H-bond from Y137 to the catalytic aspartate D243. If this interaction is critical to the activation mechanism a simplified pyrimidine activator such as **5** should retain a good degree of activation. Alternatively, if a direct interaction between the substrate aglycone and the fragment is important, removing the fused phenyl ring from the activator should reduce enzyme activation. We also considered if we retained the fused ring whether other interactions between **3** and BtGH84 could be related to the activation effects.

One such interaction observed in the X-ray crystal structures is formed between the nitrogen at position 1 of the quinazoline ring and R347, for compound **3**, and water-mediated interactions with the linker in the case of **4** (Fig. 3b). Compounds **6** and **7** remove the two ring nitrogens at positions 1 and 3 respectively, to explore if either is critical to enzyme activation. Further interactions identified in our previously published work,^25^ such as changes to the ethoxy group at position 4, led to improved activation. Compound **8** is designed to mimic these improved non-covalent activators. We also considered whether mobility of the tether between the varying positions seen in the X-ray crystal structures could promote or hinder activation. Compounds **9** and **10** attempt to coordinate the water seen H-bonded to H433 in the structure of BtGH84_TM-**4**, potentially stabilising the conformation seen in this structure and reducing linker mobility.

Modification of BtGH84_TM with the acrylamide containing fragments **5–10** using the same protocol as **4** resulted in incomplete conversion to the desired products. This is perhaps due to reduced reactivity of these compounds or weaker binding to the enzyme activator site. Tethering conditions were modified by altering the pH of the reaction buffer to affect the protonation state of Y550C – changing the proportion of the reactive thiolate anion. Increasing the pH led to better conversion to the modified protein, with over 90% conversion in 2 hours at pH 7.8 (ESI Fig. 1†). Labelling BtGH84_TM overnight at pH 7.8 with **5–10** led to complete conversion to the desired products as demonstrated by thiol quantification and mass spectrometry (ESI Fig. 1†).

### Investigating the mechanism of activation

Successful production of homogenous samples of tether labelled BtGH84_TM permitted us to obtain Michaelis–Menten kinetic parameters, Table 1, to quantify activation for each modification. Activity of BtGH84_TM-**5** was significantly lower than seen for BtGH84_TM-**4**, with only a small 1.4-fold increase in *k*_cat_/*K*_M_, compared to wild-type BtGH84. The lack of activation with this tether suggests that the Y137 π-stacking interaction is not sufficient for activation and perhaps had limiting effects on the *V*_max_ of BtGH84_TM. In contrast, the tethers designed to investigate the importance of the nitrogen position in the quinazoline ring, BtGH84_TM-**6** and BtGH84_TM-**7**, were both able to increase activity above that of the wild-type enzyme. These conjugates showed 18 and 4-fold increases in *k*_cat_/*K*_M_ over BtGH84 respectively. The much higher activity of BtGH84_TM-**6** gives a clear preference for the nitrogen position that retains the interactions observed in the X-ray crystal structures; in fact BtGH84_TM-**6** demonstrated the highest activity of the fragment modified enzymes investigated in this work. The tether based on the compounds showing the highest activation in our previous work on non-covalent activators,^25^ BtGH84_TM-**8** (crystal structure shown in Fig. 3c), increased *k*_cat_/*K*_M_ 11-fold, a similar change to BtGH84_TM-**4**. This suggests that when tethered to BtGH84 the morpholino and thiophene groups have little effect on the activating behaviour of the modification. BtGH84_TM-**9** and BtGH84_TM-**10** were intended to coordinate a water molecule with H433. These modifications increased enzyme *k*_cat_/*K*_M_ by 15 and 10-fold respectively, a significant change in activity but not an improvement over BtGH84_TM-**6**. The varying activities of each of these enzyme-fragment conjugates demonstrate that specific interactions between the modification and the protein are required for activation. All the activator modifications retain the ability to form a π-stacking interaction with Y137, potentially affecting the behaviour of this residue in BtGH84 catalysis.

Inspection of the 3-D structure of the catalytically competent conformation of BtGH84 (Fig. 3b) indicated an H-bond between Y137 and D243. We hypothesised that stabilization of this interaction might contribute to the mechanism of fragment activation. To test this hypothesis, we generated variants of BtGH84 and BtGH84_TM containing a Y137F mutation, BtGH84_Y137F and BtGH84_QM (signifying quadruple mutant) respectively, which will not have an H-bond to the catalytic D243. Michaelis–Menten kinetic analysis of these variants, ESI Table 2,† showed reduced activity when compared to BtGH84 and BtGH84_TM demonstrating that maintaining the Y137 to D243 interaction contributes to efficient catalysis in the wild-type enzyme.

**Fig. 4:**
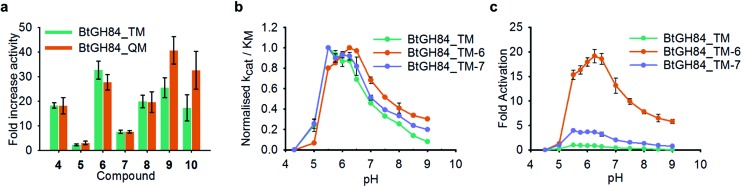
Effect of Y137F mutation and buffer pH on BtGH84 activation. (a) Fold increase in specificity constant over the parent enzyme for BtGH84_TM and BtGH84_QM fragment conjugates. BtGH84_TM and BtGH84_QM activation are shown as green and orange bars respectively. (b) and (c) BtGH84_TM (green), BtGH84_TM-**6** (orange), and BtGH84_TM-**7** (blue) activity across pH range 4.3–9.0. Activity in (b) is individually normalised to the highest activity for each conjugate for ease of comparison. Activity in (c) is normalised to BtGH84_TM at pH 5.5.

In order to understand whether activation of BtGH84 also depends on stabilisation of this key interaction we generated tether modified versions of BtGH84_QM in an analogous manner to BtGH84_TM. The tether variants of BtGH84_QM showed increases in activity, measured as *k*_cat_/*K*_M_, over the parent enzyme BtGH84_QM comparable to those seen for BtGH84_TM conjugates when compared to BtGH84_TM, Fig. 4a and ESI Table 3.† This suggests that the interaction between Y137F and D243 is not critical to the activation mechanism. As an alternative explanation we considered whether direct interaction between the substrate and activator could be responsible for increasing activity.

Each of the covalent modifications investigated in the current work, with the exception of **5**, contain an aromatic ring that can be orientated towards the active site and form a potential interaction with the aglycone group of an inhibitor or a substrate. To investigate this interaction we considered that PUGNAc could be an approximate surrogate for 4MU-GlcNAc based on structural similarity (ESI Fig. 8†). We measured the affinity of BtGH84_TM-fragment conjugates for PUGNAc using ITC, Table 1, ESI Table 4 and Fig. 3.† There is correlation between affinity and degree of activation for some of the modifications, although the enzyme modified with the best activator, **6**, binds PUGNAc some 7-fold more weakly than when modified with the weaker activator, **8**, perhaps because the large morpholino substituent of **8** is more suited to interact with PUGNAc than with 4MU-GlcNAc. In contrast, affinities of thiamet-G, which lacks a hydrophobic aglycone group, for the BtGH84-fragment conjugates, showed no correlation, Table 1.

These changes in binding affinity for PUGNAc suggest that similar changes in 4MU-GlcNAc affinity should lead to an increase in the catalytic rate. One possible explanation is that the direct interaction between substrate and tether modification could alter the pH dependency of enzyme activity by improving the ability of 4MU to act as the leaving group. To investigate this, we measured the modified BtGH84_TM enzyme activity across a 12-point pH range, generating pH-activity profiles for each of the BtGH84_TM conjugates, Fig. 4b and c (full data shown in ESI Fig. 7†). While the changes in the pH optima are moderate, the tethered enzymes that showed strong activation such as with **6**, **9** and **10** have a higher pH optima of 6.25–6.5, than the unconjugated enzyme or enzyme conjugated with poor activating modifications such as **5** or **7**, which range from pH 5.5–6.0. In addition the degree of activation seen at pH values above the optima is greater than at the pH optima, ESI Fig. 7.† This slight change in pH preference suggests that the tethers may affect the protonation of the leaving group at high(er) pH in addition to altering the binding affinity of the substrate. This data provides the first steps to understanding the mechanism of BtGH84 activation by covalent modification for the 4MU-GlcNAc.

## Discussion

Many different protein engineering approaches to changing biocatalyst activity have been developed over the past 30 years with applications including improved enzymes for production of pharmaceuticals,^36^ fine chemicals,^37^ lab-based biocatalysis for synthetic chemistry^38^ and for use in industrial processes such as biofuel generation.^39^ Perhaps the most powerful and widely used approach is directed evolution, where random mutagenesis by error-prone PCR generates extremely large libraries of enzyme variants.^40,41^ Improved enzymes are identified either by selection based upon survival advantage or by high-throughput methods of analysing activity.^42^ Such directed evolution is powerful, but can only explore the limited chemistries available through the genetic code. There has been some work exploring incorporation of unnatural amino acids acids using synthetic biology methods,^43^ but the scope is limited by the number of modified tRNAs available.

In comparison to these biological approaches, chemical modification methods are less common. PEGylation of industrial enzymes has been used to improve physico-chemical properties, as reviewed in ref. 44, and there was early exploration of chemical modification of mutant subtilisin. However, such small molecule approaches have not been widely used. Fragment-based approaches are now well established for lead discovery in the pharmaceutical industry^45^ and the work presented here demonstrates how these can provide an alternative method to expand and alter the chemistry of enzymes.

We have demonstrated the design of a covalent tether to optimise the previously-observed^25^ binding mode of a non-covalent activator of BtGH84. Remarkably, not only was the binding mode retained but the effects of the modification on enzyme activity were more profound than those seen with non-covalent activators, with a greater than 10-fold (up to 35 fold) increase in specific activity for the most active enzyme-fragment conjugates. Only limited optimisation of the chemistry of the modification was carried out so there is potential for even larger effects.

We have shown that the mechanism of activity enhancement is consistent with a direct interaction between substrate and the modification – effectively allowing binding site optimisation for the substrate leaving group aglycone. Furthermore, given that the activation is *via* the aglycone and not the “-1 subsite” sugar, covalent activator modifications similarly improve the binding affinity of an “aglycone-containing” inhibitor PUGNAc, but have no effect on a sugar mimicking inhibitor such as thiamet-G. Perturbation of enzyme dynamics and conformation of the “catalytic loop” by the modifications, may also contribute to the observed effects.

Our results also demonstrate that the degree of activation is dependent on subtle combinations of interactions that can be achieved between activator, enzyme and substrate. This suggests that the initial non-covalent fragment activator should be identified in assays with the target substrate. Further work on the mechanism of activation will require consideration of enzyme dynamics and the impact of the modifications on the rate limiting chemical steps.

## Conclusion

In this study we have demonstrated proof of principle for a fragment-based discovery approach to enzyme engineering that could prove complementary to directed evolution and *de novo* design. The general strategy is to use sensitive biophysical methods to screen relevant targets to identify weak binding fragments. These fragment hits are then assessed in appropriate assays to identify fragments that bind allosterically or increase enzyme activity. If possible, the fragments are optimised at this stage to identify improved activity. Crystal structures of the bound fragments can then inform the design of linkers and the design of further chemical modifications to improve the activator as well as to aid introduction of appropriate synthetic handles on the enzymes.

As with fragment based discovery of inhibitors, we think this approach to enzyme engineering may have general applicability, not only for improving the performance of enzymes, but also for developing probes to investigate how modulating enzyme activity can impact on studies of cell biology, either through increasing activation or improving the affinity of inhibitors.

## Conflicts of Interest

The authors declare no competing financial interests in the work presented in this paper.
