# Supplementary material for: Increase of enzyme activity through specific covalent modification with fragments
Source: Chem Sci. 2017 Sep 27;8(11):7772–9. doi: 10.1039/c7sc01966a (PMC5674511; doi:10.1039/c7sc01966a)

Electronic Supplementary Material (ESI) for Chemical Science.
This journal is © The Royal Society of Chemistry 2017

Supplementary Information for

Increase of enzyme activity through specific covalent modification with fragments

John F. Darby[1], Masakazu Atobe[1,2], James D. Firth[1], Paul Bond[1], Gideon J. Davies[1], Peter O'Brien[1], Roderick E. Hubbard*[1,4]

Contents

Methods 3
Chemicals and reagents 3
Modelling of the linking strategy. 3
Expression, purification, crystallisation, structure determination of BtGH84_TM. 3
Tether modification protocol 5
Protein mass spectrometry 5
Ellman's reagent assay 6
Activity assay with 4MU-GlcNAc 7
ITC methods 7
Tables 8
Supplementary Table 1: Data collection and refinement statistics (molecular replacement) for structures of BtGH84-**4**, BtGH84-**4** PUGNAc, BtGH84-**4** Thiamet G, BtGH84-**8** PUGNAc 8
Supplementary Table 2: Kinetic data for unmodified protein and unmodified mutants 9
Supplementary Table 3: Kinetic data for modified BtGH84_QM mutant 10
Supplementary Table 4: Thermodynamic data for BtGH84_TM binding to PUGNAc 11
Figures 12
Supplementary Figure 1: Ellman's reagent results. 12
Supplementary Figure 2: Michaelis-Menten plots of enzyme conjugates. 13
Supplementary Figure 3: ITC data 14
Supplementary Figure 4: Comparison of the binding site in modified BtGH84_TM structures. 16
Supplementary Figure 5: Comparison of the binding site in modified BtGH84_TM structures to noncovalent activator binding. 17
.... 17
Supplementary Figure 6: Comparison of the full structures of modified BtGH84_TM proteins 18
Supplementary Figure 7: Comparison of BtGH84_TM conjugate activity across pH 19
Supplementary Figure 8: Structure of BtGH84_TM-**4** with substrate 4MU-GlcNAc model 20
Supplementary Figure 9: Overlays of modified BtGH84_TM structures 20

Supplementary Figure 10: Activity is not protein concentration dependent 21
Data 22
Intact protein mass spectrometry of BtGH84_TM and covalently modified BtGH84_TM.............. 22
Synthesis and compound characterisation 28
Description of synthetic strategy – Schemes S1 – S5.. 28
Experimental Details 31
References for synthesis section.. 48
NMR Spectra.. 49

Methods

Chemicals and reagents

Reagents of appropriate purity were purchase from Sigma unless otherwise noted. O-(2-Acetamido-2-deoxy-D-glucopyranosylidene) amino-Z-Nphenylcarbamate (PUGNAc) was purchased from Santa Cruz Biotechnology.

Modelling of the linking strategy.

Tether models were constructed using Discovery Studio 3.5 [1]. Coordinates of 4-ethoxyquinazoline (PDB: BK9) and the residue chosen for the tether were copied from 4UR9. The tether was then built from this by adding and removing atoms and manually rotating bonds. The resulting models were typed in CHARMm. Fixed atom constraints were added to N, CA, C and O of the amino acid, as well as the ring atoms and oxygen atom of BK9 (including hydrogens bonded to these atoms). Three rounds of a Standard Dynamics Cascade followed by a Minimisation were performed using default parameters to produce the final tether model.

[1] Discovery Studio, version 3.5. Accelrys Inc., San Diego, CA, USA: 2012.

Expression, purification, crystallisation, structure determination of BtGH84_TM.

The BtGH84 gene was expressed and purified as previously described [1]. Mutants were cloned using site-directed mutagenesis and expressed and purified in a manner analogous to wild-type BtGH84. Site-directed mutagenesis was performed with the Gibson Assembly method (New England Biolabs). Primers containing the appropriate mutations were used to prepare linear DNA amplicons with 16-22 bp overlaps. These fragments were mixed with linearised plasmid DNA with complementary overlaps and combined using the Gibson Assembly master mix. This allowed the introduction of multiple mutations in a single step. The resulting clones were fully sequenced to confirm that the correct mutations had occurred.

Primer sequences for BtGH84_TM - C420S, Y550C, C654S (5’ → 3’)
Overlaps:
F1+R5,F2+R1,F3+R2,F4+R3,F5+R4
PCR pairs:
F1+R1,F2+R2,F3+R3,F4+R4,F5+R5
F1
AACTTTAAGAAGGAGATATACCATGGGCAG
F2
AACTTGAAAGCTTTGCAATGCACAATTCCGA
F3
CAGAATCCTTGCCAACCGGGAGTAAA
F4
GGATGCTCCTAGCACATGGGGACGCC
F5
CGCGCCTTCTCCTCACTGTTCCA

R1

CATTGCAAAGCTTTCAAGTTCTTCCGCT
R2
GGTTGGCAAGGATTCTGATTACTCGTC
R3
ATGTGCTAGGAGCATCCTTACCAAAGTTTATC
R4
GTGAGGAGAAGGCGCGTTATTTCTTCT
R5
GGTATATCTCCTTCTTAAAGTTAAACAAAATTATTTCT

For Y137F mutants the BtGH84 and BtGH84_TM plasmids were modified using a similar approach to previously using the following primers:
Forward (5' → 3')
AAGGGTTTTTTGGAACTCCCTGGAGC
Reverse (5' → 3')
GAGTTCCAAAAAACCCTTCTACCACTC

BtGH84_TM crystals were grown using the sitting-drop vapour diffusion method. 1 μl of 12 mg/ml BtGH84 was mixed with 1 μl of reservoir solution (125 mM imidazole pH 8.0, 4% PEG8000 (w/v), 3% trimethylamine N-oxide-dehydrate, 15% ethylene glycol) and the crystals were allowed to grow at room temperature for at least 2 days. Crystal soaking was performed by gradually adding well solution containing 10 mM PUGNac or Thiamet G. Crystals were picked with a nylon fibre loop (Hampton Research) and plunged into liquid nitrogen. Data were collected at Diamond beamline I02 or I03 using a Pilatus 6M-F or Pilatus3 6M detector respectively (Dectris) at 100 K. An oscillation range of 0.1° degree was chosen and 180° were collected. Data were autoprocessed in the Diamond xia2 pipeline, data reduction was carried out using Aimless (CCP4i2) and molecular replacement performed using MolRep (CCP4i2) from PDB 2CHO (A chain only). The model was improved and adjusted by alternating cycles of manual rebuilding and real-space refinement in COOT [2] and reciprocal-space refinement in Refmac [3] and Phenix [4]. Coordinate files and libraries for the ligand were created using Make Ligand (CCP4i2) or eLBOW (Phenix) from a SMILES string of the ligand. The quality of the final model was evaluated using the MolProbity-Server [5]. Figures were created using CCP4MG.

Resultant structures have been deposited in the Protein Data Bank as 5MI4, 5MI5, 5MI6, 5MI7 for BtGH84_TM-**4**, BtGH84_TM-**4** with PUGNAc, BtGH84_TM-**4** with thiamet G, and BtGH84_TM-**8** with PUGNAc respectively.

[1] Dennis et al. Nature Structural & Molecular Biology. 2006, 13, 365-371

[2] P. Emsley, B. Lohkamp, W. G. Scott, K. Cowtan, Acta crystallographica. Section D, Biological crystallography 2010, 66, 486-501.

[3] G. N. Murshudov, A. A. Vagin, E. J. Dodson, Acta crystallographica. Section D, Biological

crystallography 1997, 53, 240-255.

[4] P. D. Adams, P. V. Afonine, G. Bunkoczi, V. B. Chen, I. W. Davis, N. Echols, J. J. Headd, L. W. Hung, G. J. Kapral, R. W. Grosse-Kunstleve, A. J. McCoy, N. W. Moriarty, R. Oeffner, R. J. Read, D. C. Richardson, J. S. Richardson, T. C. Terwilliger, P. H. Zwart, Acta crystallographica. Section D, Biological crystallography, 2010, 66, 213-221.

[5] V. B. Chen, W. B. Arendall, 3rd, J. J. Headd, D. A. Keedy, R. M. Immormino, G. J. Kapral, L. W.Murray, J. S. Richardson, D. C. Richardson, Acta crystallographica. Section D, Biological crystallography. 2010, 66, 12-21.

Tether modification protocol

Modification of BtGH84_TM (and BtGH84_QM) by acrylamide tether compounds **4-10** at position Y550C was performed in aqueous conditions at 4°C or RT. Initially, 60-100 µM BtGH84_TM was combined with 2 mM 4 (from 100 mM stock in MeOH) in buffer (50 mM MES, pH 6.5, 200 mM NaCl) with 0.5mM THP (Tris(3-hydroxypropyl)phosphine as a disulphide reducing agent). Reaction volumes of 60-120 µl were incubated for 4 hours at RT. The subsequently optimised labelling reaction typically comprised 100 µM BtGH84_TM or BtGH84_QM combined with 2-4 mM acrylamide-containing tether compound (from 100 mM stock in MeOH) in buffer (200 mM Tris, pH 7.8, 200 mM NaCl) with 0.5mM THP. Reaction volumes of 60-120 µl were incubated overnight at 4 °C. For all reactions the resulting product was separated from unreacted labelling compound using Zeba spin desalting columns, 7 kDa MWCO (Thermo). This clean-up step enabled simultaneously transfer of the product into appropriate buffer for the subsequent application.

Finally, all protein concentration values were determined via absorbance at 280 nm using extinction coefficients calculated using the ExPASy ProtParam tool.

Protein mass spectrometry

Electrospray Mass Spectrometry was conducted on a Waters LCT Premier XE system with MassLynx 4.1 software. The system was calibrated with sodium formate solution and calibration verified/corrected with horse heart myoglobin (16951.5 ± 1.5 Da).

Samples were purified from salt containing buffer solutions into ammonium acetate buffer using Zeba Spin Desalting Columns (7K MWCO, 0.5 mL) according to the manufacturer's protocol. Samples in ammonium acetate buffer were diluted directly into 1:1 acetonitrile-water containing 0.1% formic acid.

Sample was infused into the MS source using a syringe pump. When a stable signal was achieved, data was collected for 3 minutes over a scan range of 200 - 2000 m/z. The scans were combined and baseline subtracted. For samples of unknown mass, the spectrum was smoothed and centroided, then peak series identified by eye and measured using the Manual Find Component procedure in MassLynx. When the components present were known or identified, the data in the m/z range containing the peak series was then processed using the Maxent1 procedure over an appropriate mass range with suitable peak half-width settings determined from the raw data.

The resultant deconvoluted data is shown in the Supplementary Data section below with corrected masses noted for the major species. The secondary peak seen at +178.6 Da ± 2.49 Da in each spectra is likely to be α-N-6-phosphogluconoylation of the His-tag as previously seen in recombinantly expressed proteins in *E. coli*. [1]

[1] Geoghegan et al. Spontaneous α-N-6-Phosphogluconoylation of a “His Tag” in Escherichia coli:The Cause of Extra Mass of 258 or 178 Da in Fusion Proteins. Analytical Biochemistry **267** (1) 169-184.

Ellman’s reagent assay

Estimation of thiol concentration in samples of unmodified and modified BtGH84_TM and BtGH84_QM was carried out using Ellman’s reagent (5,5’-dithiobis(2-nitrobenzoic acid) or DTNB) which reacts with free thiols to release the coloured 2-nitro-5-thiobenzoic acid (TNB) anion [1]. This quantification was performed by combining 40 µl protein sample (150 µM), with 33 µl DTNB (10 mM in 0.1 M Na_2HPO_4, pH 8.0) and 927 µl of denaturing buffer (6 M guanidinium chloride in 0.1 M Na_2HPO_4, pH 8.0). Absorbance at 412 nm was recorded using a Cary 100 UV-Vis spectrophotometer. Values were collected for the denaturing buffer alone, following the addition of DTNB and finally after addition of the protein sample. The concentration of thiol present was calculated using the molar absorbance of the TNB anion (E_{412} TNB^{2-} = 1.37 x 10^4 M^{-1} cm^{-1}) from the background corrected values for absorbance. This was compared to the protein concentration calculated using absorbance at 280 nm to determine the ratio of labelled protein. Unlabelled denatured protein contains two thiols per protein molecule whereas fully labelled denatured protein contains a single thiol per protein molecule.

[1] Aitken, A. and M. Learmonth (2003). "Quantitation and location of disulfide bonds in proteins." Methods Mol Biol **211**: 399-410.

Activity assay with 4MU-GlcNAc

Measurements of enzyme activity were performed using a fluorescent kinetic assay. Assays were carried out in a 96-well plate (Black Nunc F96 MicroWell Plates) at 25°C in a total volume of 100 µl per well. Each well contained 90 µl buffer (50 mM MES pH 6.5, 200 mM NaCl, 0.01% Tween-20) and substrate 4-methylumbelliferyl-β-D-GlcNAc (4MU-GlcNAc) across a 2-fold serial dilution range from 4 mM – 3.91 µM. Reactions were initiated by addition of 10 µl of protein, to a final concentration of 50 nM, followed by 5 s of shaking. Fluorescent 4-methylumbelliferyl release was recorded continuously at 355 nm excitation and 460 nm emission for 10 s using a BMG PolarStar Optima plate reader. Initial velocity rates were calculated using Sigmaplot as the slope of the curve of the collected data. Corrections were made to the detected gradients to adjust for the inner filter effect of 4MU-GlcNAc and convert the gradients into units of µM min^{-1} according to a standard curve of 4MU. Using this data Michaelis-Menten plots of initial velocity versus substrate concentration were fitted to the Michaelis-Menten equation in SigmaPlot. Values quoted throughout are mean and standard deviation of three independent plate replicates with each replicate containing either duplicate or triplicate on-plate wells.

Additional experiments (results in Supplementary Figure 10) were performed to demonstrate that the enzyme kinetics is not dependent on protein concentration.

ITC methods

Isothermal titration calorimetry (ITC) measurements were carried out using a MicroCal Auto-iTC200 (Malvern Instruments) at 25°C. BtGH84 was prepared in filtered and degassed 50 mM MES, pH 6.5, 200 mM NaCl, and concentrated to an appropriate range. PUGNAc and Thiamet G were prepared in the same buffer. Samples were centrifuged and degassed prior to use.

Syringe concentrations of PUGNAc and Thiamet G were 240-360 µM and 180-190 µM respectively, final concentrations were calibrated and corrected by collecting ^{1}H NMR spectra with a DSS standard at 100 µM. Cell concentrations of BtGH84 or BtGH84_TM were 20-40 µM and 15 µM for PUGNAc and Thiamet G titrations respectively. Titrations were performed by an initial injection of 0.5 µL followed by 19 x 2 µl injections of the inhibitor into the BtGH84 solution with stirring at 750 rpm.

Using Microcal PEAQ-ITC Analysis software (v1.1.0.1262) the enthalpy for each injection was plotted against the molar ratio and fitted to a bimolecular model. The stoichiometry (N), enthalpy (ΔH), and association constant (Ka) were obtained from this fitting, and were used to calculate the Gibbs free energy (ΔG) and entropy (TΔS).

Tables

*Supplementary Table 1: Data collection and refinement statistics (molecular replacement) for structures of BtGH84-**4**, BtGH84-**4** PUGNAc, BtGH84-**4** Thiamet G, BtGH84-**8** PUGNAc*

	**BtGH84_TM-4**	**BtGH84_TM-4 PUGNAc**	**BtGH84_TM-4 Thiamet G**	**BtGH84_TM-8 PUGNAc**
**PDB accession**	5MI4	5MI5	5MI6	5MI7
**Data collection**				
**Space group**	C121	C121	C121	C121
**Cell dimensions**				
***a*, *b*, *c* (Å)**	195.03, 51.66, 111.87	198.22, 52.12, 112.78	196.02, 51.66, 112.26	195.19, 51.82, 108.23
**α,β,γ (°)**	90.00, 113.41, 90.00	90.00, 113.24, 90.00	90.00, 113.34, 90.00	90.00, 111.61, 90.00
**Resolution (Å)**	57.14-1.80 (1.83-1.80)*	51.81-2.15 (2.21-2.15)*	49.66-2.00 (2.04-2.00)*	100.62-2.10 (2.16-2.10)*
R_{merge}	0.052(0.375)	0.131(0.588)	0.112(0.75)	0.08(0.73)
$I / \sigma I$	12.1(2.8)	4.3(1.2)	4.4(1.2)	6.9(1.5)
**Completeness (%)**	100(99.9)	99.8(99.6)	99.5(97.2)	100(99.7)
**Redundancy**	4.1(4.2)	3.9(4.1)	3.1(3.0)	4.0(4.0)
**Refinement**				
**Resolution (Å)**	57.14-1.8	51.81-2.15	103.07-2.00	100.62-2.10
**No. reflections**	95258	58090	69993	59272
R_{work} / R_{free}	0.16 / 0.20	0.23/0.27	0.20/0.24	0.21/0.25
**No. atoms**	6406	5785	5971	6042
**Protein**	5659	5489	5598	5991
**Ligand/ion**	43	44	51	5
**Water**	704	252	322	141
***B*-factors**				
**Protein**	29.26	49.26	36.46	52.52
**Ligand/ion**	44.60	51.77	44.83	68.28
**Water**	38.58	45.12	37.06	40.83
**R.m.s. deviations**				
**Bond lengths (Å)**	0.0146	0.0084	0.0134	0.0041
**Bond angles (°)**	1.563	0.892	1.586	0.581

*A single crystal was used for each dataset. *Values in parentheses are for highest-resolution shell.

[AU: Equations defining various *R*-values are standard and hence are no longer defined in the footnotes.]
[AU: Ramachandran statistics should be in Methods section at the end of Refinement subsection.]

Data was collected at the Diamond synchrotron funded under grants mx-13587 and mx-9948

Supplementary Table 2: Kinetic data for unmodified protein and unmodified mutants

		BtGH84	BtGH84_TM	BtGH84 Y550C	BtGH84_QM	BtGH84 Y137F
V_{max}	μM/min	200 ± 14	381 ± 19	251 ± 73	232 ± 44	262 ± 11
K_m	μM	1462 ± 223	4682 ± 224	5568 ± 875	11215 ± 3274	6610 ± 1282
k_{cat}	1/s	67 ± 5	127 ± 6	84 ± 24	77 ± 15	87 ± 4
k_{cat}/K_m	1/Msec	46086 ± 5897	27209 ± 2459	14838 ± 2029	7054 ± 821	13483 ± 2017
**Fold**	WT	1.0 ± 0.13	0.6 ± 0.05	0.3 ± 0.04	0.2 ± 0.02	0.3 ± 0.04

Supplementary Table 3: Kinetic data for modified BtGH84_QM mutant

		4	5	6	7	8	9	10
V_{max}	μM/min	224 ± 27	40 ± 3	319 ± 44	116 ± 20	124 ± 1	301 ± 29	253 ± 16
K_m	μM	597 ± 179	626 ± 101	551 ± 135	731 ± 178	305 ± 69	356 ± 83	380 ± 113
k_{cat}	1/s	75 ± 9	13 ± 1	106 ± 15	39 ± 7	41 ± 0.5	100 ± 10	84 ± 5
k_{cat}/K_m	1/Msec	128547 ± 23187	21667 ± 5030	195418 ± 21626	53523 ± 3866	138861 ± 29860	287002 ± 39796	230158 ± 54350
Fold	WT	2.8 ± 0.5	0.5 ± 0.1	4.2 ± 0.5	1.2 ± 0.1	3.0 ± 0.6	6.2 ± 0.9	5.0 ± 1.2
Fold	Parent	18.2 ± 3.3	3.1 ± 0.7	27.7 ± 3.1	7.6 ± 0.5	19.7 ± 4.2	40.7 ± 5.6	32.6 ± 7.7

Supplementary Table 4: Thermodynamic data for BtGH84_TM binding to PUGNAc

Protein	***Ligand***	***K_d (nM)***	***N***	***ΔH (kcal/mol)***	***ΔG (kcal/mol)***	***TΔS (kcal/mol)***
BtGH84_TM	PUGNAc	2605 ± 106	1.02 ± 0.05	-7.12 ± 0.74	-7.62 ± 0.03	-0.50 ± 0.76
BtGH84_TM-**4**	PUGNAc	172 ± 9.9	0.96 ± 0.08	-8.70 ± 0.33	-9.23 ± 0.03	-0.53 ± 0.30
BtGH84_TM-**5**	PUGNAc	829 ± 533	1.07 ± 0.16	-6.40 ± 0.81	-8.37 ± 0.35	-1.98 ± 1.11
BtGH84_TM-**6**	PUGNAc	522 ± 204	1.03 ± 0.06	-7.87 ± 0.97	-8.60 ± 0.23	-0.73 ± 1.21
BtGH84_TM-7	PUGNAc	2547 ± 582	0.80 ± 0.1	-8.32 ± 0.14	-7.64 ± 0.15	0.68 ± 0.10
BtGH84_TM-**8**	PUGNAc	77 ± 7.7	0.98 ± 0.04	-7.27 ± 0.22	-9.71 ± 0.06	-2.44 ± 0.23
BtGH84_TM-**9**	PUGNAc	780 ± 135	1.13 ± 0.08	-7.51 ± 0.25	-9.23 ± 0.85	-1.72 ± 1.08
BtGH84_TM-**10**	PUGNAc	ND				
BtGH84_TM	Thiamet G	52 ± 5.7	1.02 ± 0.02	-8.28 ± 0.06	-9.94 ± 0.06	-1.66 ± 0.13
BtGH84_TM-**4**	Thiamet G	50 ± 7.6	0.95 ± 0.04	-8.79 ± 0.59	-9.95 ± 0.07	-1.18 ± 0.68
BtGH84_TM-**5**	Thiamet G	45 ± 15.6	0.99 ± 0.03	-9.53 ± 0.95	-10.1 ± 0.22	-0.52 ± 1.14
BtGH84_TM-**6**	Thiamet G	51 ± 10.1	0.97 ± 0.02	-8.94 ± 0.57	-9.94 ± 0.09	-1.01 ± 0.44
BtGH84_TM-**7**	Thiamet G	73 ± 20.9	0.94 ± 0.01	-9.22 ± 0.85	-9.75 ± 0.17	-0.54 ± 1.02
BtGH84_TM-**8**	Thiamet G	44 ± 15.0	0.95 ± 0.04	-9.07 ± 0.59	-10.1 ± 0.21	-0.99 ± 0.38
BtGH84_TM-**9**	Thiamet G	59 ± 27	0.98 ± 0.02	-7.98 ± 0.27	-9.88 ± 0.25	-1.90 0.58
BtGH84_TM-**10**	Thiamet G	75 ± 16	0.98 ± 0.01	-8.74 ± 0.16	-9.86 ± 0.33	-1.14 ± 0.49

Note that the date in Supplementary Table 4 are the mean and standard deviation of 3 repeats. This gives slightly different values to those shown in Figure 2b of the main text which is for a single titration

Figures

Supplementary Figure 1: Ellman's reagent results.

(a) Labelling efficiency after 2 hours with varying pH as determined by Ellman's reagent quantification of free thiols. Increasing pH showed decreased concentration of free thiol and an increase in the labelled fraction of protein. (b) Quantification of free thiol concentration in denatured samples of BtGH84_TM conjugates. The measured concentrations are closely matched to that expected for one free thiol remaining (100% labelled). (c) Quantification of free thiol concentration in denatured samples of BtGH84_QM conjugates.

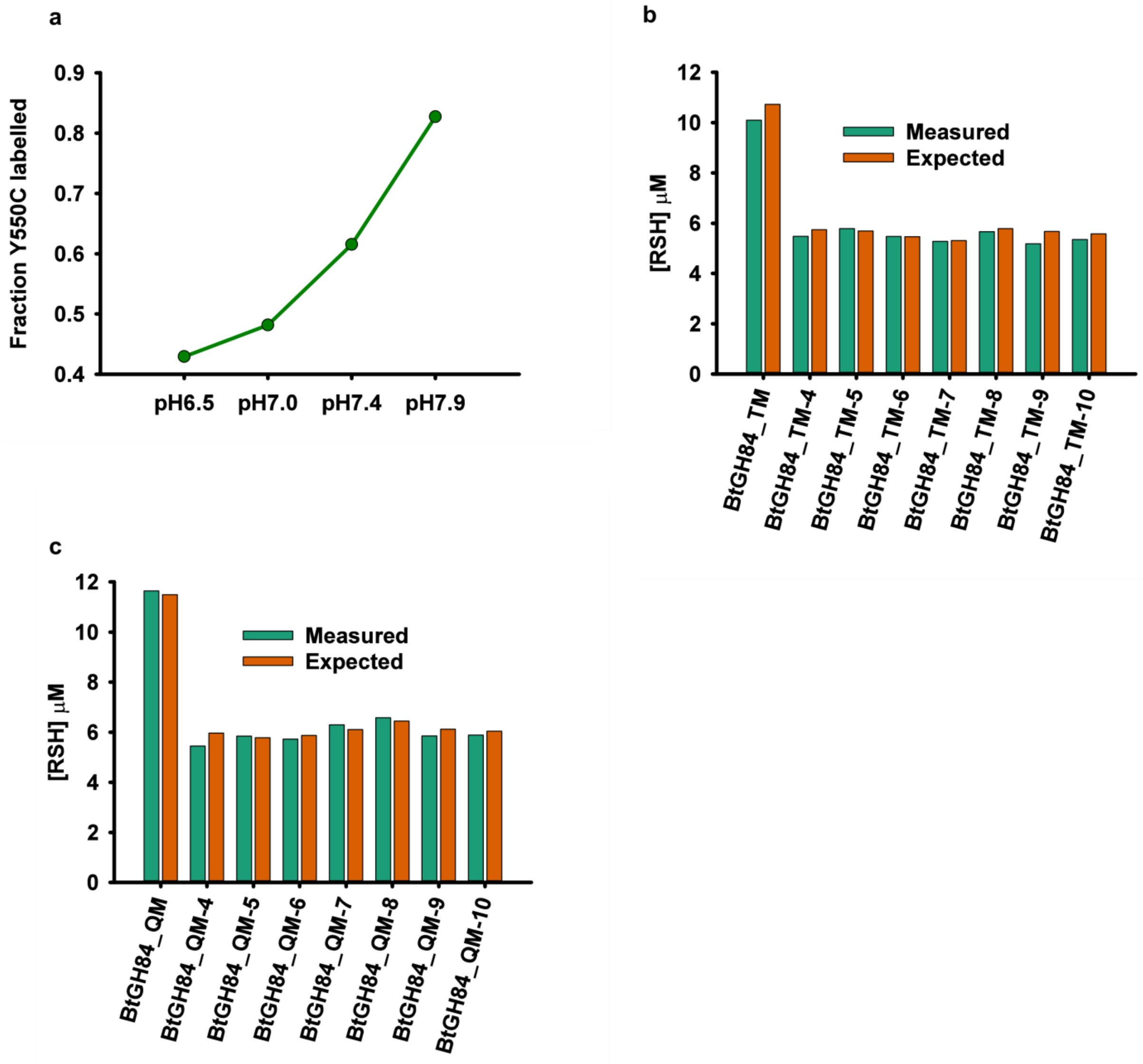

Supplementary Figure 2: Michaelis-Menten plots of enzyme conjugates.

(a) Representative Michaelis-Menten plots for BtGH84_TM-fragment conjugates obtained using the 4MU-GlcNAc substrate as described in Methods. Final kinetic parameters can be found in Table 1.

(b) Representative Michaelis-Menten plots for BtGH84_QM-fragment conjugates obtained using the 4MU-GlcNAc substrate as described in Methods. Final kinetic parameters can be found in Supplementary Table 3.

a

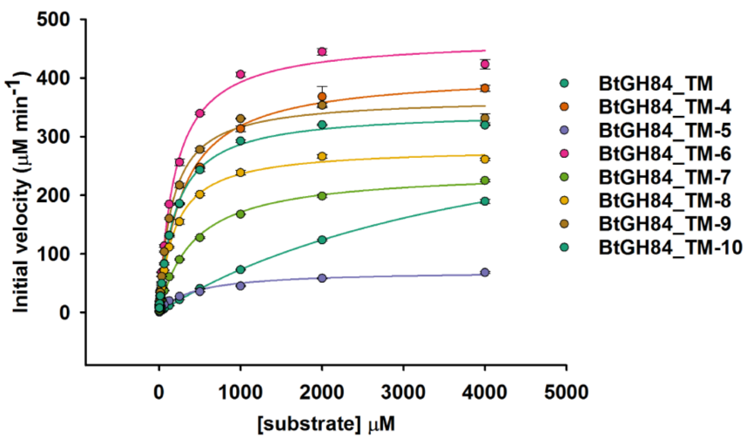

b

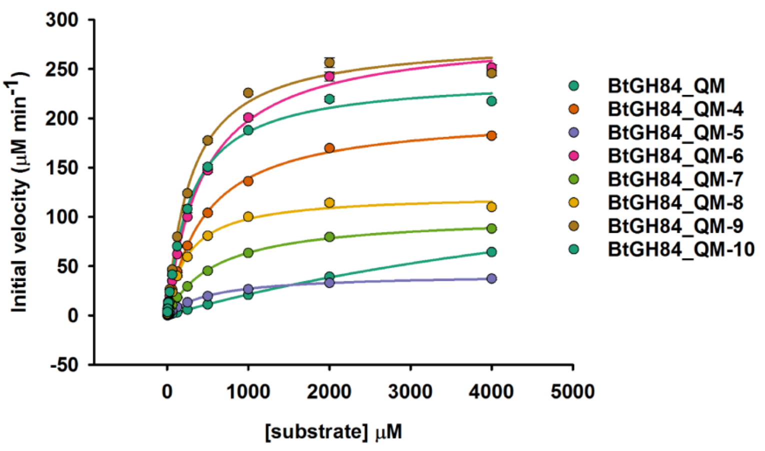

Supplementary Figure 3: ITC data

(a-g) Representative ITC data for PUGNAc binding to BtGH84_TM-fragment conjugates, panels a-g correspond to modifications **4**-**10** respectively. (h-n) Representative ITC data for thiamet-G binding to BtGH84_TM-fragment conjugates, panels h-n correspond to modifications **4**-**10** respectively.

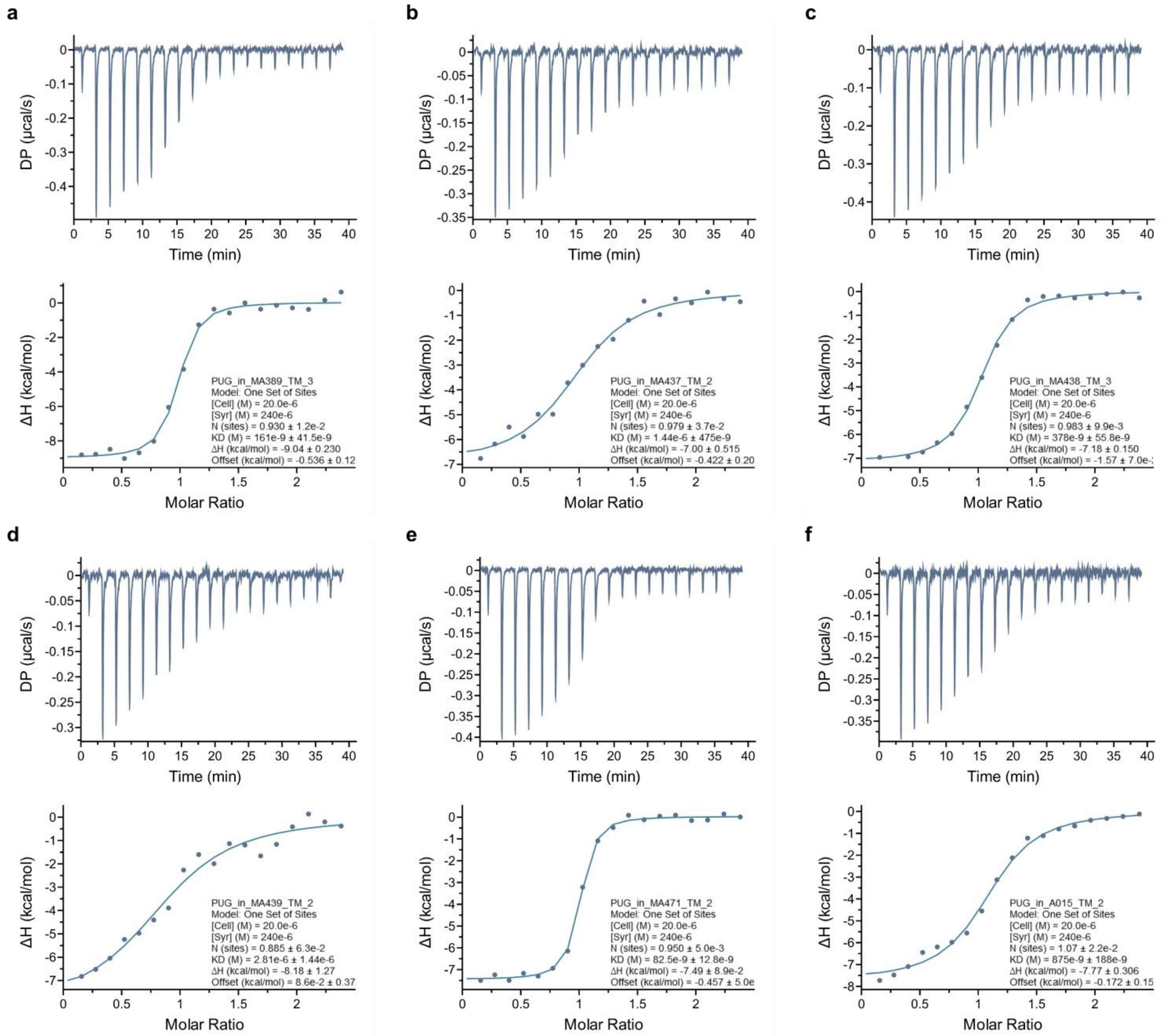

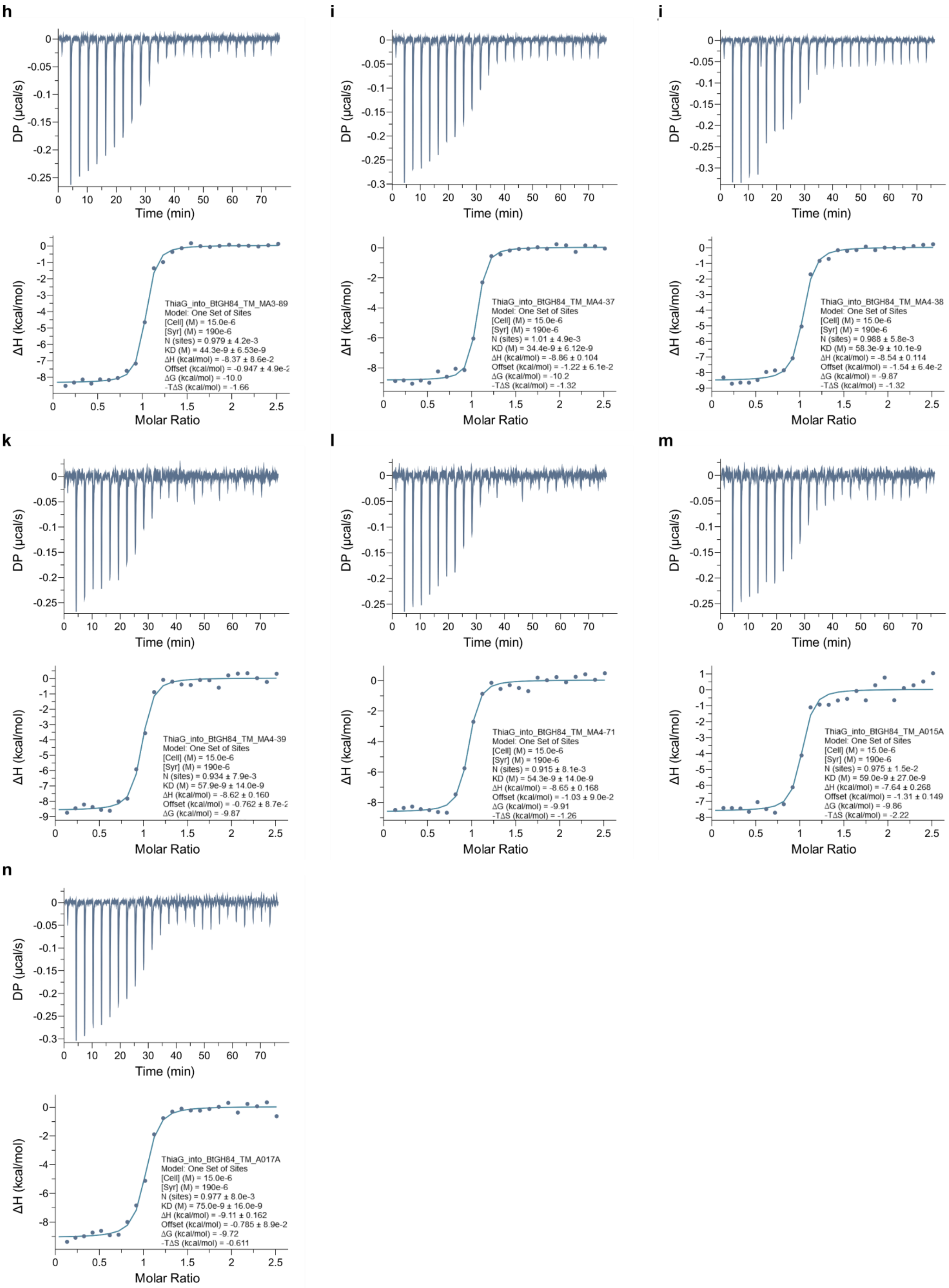
h
i
j
k
l
m
n
DP (µcal/s)
Time (min)
ΔH (kcal/mol)
Molar Ratio
ThiaG_into_BtGH84_TM_MA3-89
Model: One Set of Sites
[Cell] (M) = 15.0e-6
[Syr] (M) = 190e-6
N (sites) = 0.979 ± 4.2e-3
KD (M) = 44.3e-9 ± 6.53e-9
ΔH (kcal/mol) = -8.37 ± 8.6e-2
Offset (kcal/mol) = -0.947 ± 4.9e-[illegible]
ΔG (kcal/mol) = -10.0
-TΔS (kcal/mol) = -1.66
ThiaG_into_BtGH84_TM_MA4-37
Model: One Set of Sites
[Cell] (M) = 15.0e-6
[Syr] (M) = 190e-6
N (sites) = 1.01 ± 4.9e-3
KD (M) = 34.4e-9 ± 6.12e-9
ΔH (kcal/mol) = -8.86 ± 0.104
Offset (kcal/mol) = -1.22 ± 6.1e-2
ΔG (kcal/mol) = -10.2
-TΔS (kcal/mol) = -1.32
ThiaG_into_BtGH84_TM_MA4-38
Model: One Set of Sites
[Cell] (M) = 15.0e-6
[Syr] (M) = 190e-6
N (sites) = 0.988 ± 5.8e-3
KD (M) = 58.3e-9 ± 10.1e-9
ΔH (kcal/mol) = -8.54 ± 0.114
Offset (kcal/mol) = -1.54 ± 6.4e-2
ΔG (kcal/mol) = -9.87
-TΔS (kcal/mol) = -1.32
ThiaG_into_BtGH84_TM_MA4-39
Model: One Set of Sites
[Cell] (M) = 15.0e-6
[Syr] (M) = 190e-6
N (sites) = 0.934 ± 7.9e-3
KD (M) = 57.9e-9 ± 14.0e-9
ΔH (kcal/mol) = -8.62 ± 0.160
Offset (kcal/mol) = -0.762 ± 8.7e-[illegible]
ΔG (kcal/mol) = -9.87
ThiaG_into_BtGH84_TM_MA4-71
Model: One Set of Sites
[Cell] (M) = 15.0e-6
[Syr] (M) = 190e-6
N (sites) = 0.915 ± 8.1e-3
KD (M) = 54.3e-9 ± 14.0e-9
ΔH (kcal/mol) = -8.65 ± 0.168
Offset (kcal/mol) = -1.03 ± 9.0e-2
ΔG (kcal/mol) = -9.91
-TΔS (kcal/mol) = -1.26
ThiaG_into_BtGH84_TM_A015A
Model: One Set of Sites
[Cell] (M) = 15.0e-6
[Syr] (M) = 190e-6
N (sites) = 0.975 ± 1.5e-2
KD (M) = 59.0e-9 ± 27.0e-9
ΔH (kcal/mol) = -7.64 ± 0.268
Offset (kcal/mol) = -1.31 ± 0.149
ΔG (kcal/mol) = -9.86
-TΔS (kcal/mol) = -2.22
ThiaG_into_BtGH84_TM_A017A
Model: One Set of Sites
[Cell] (M) = 15.0e-6
[Syr] (M) = 190e-6
N (sites) = 0.977 ± 8.0e-3
KD (M) = 75.0e-9 ± 16.0e-9
ΔH (kcal/mol) = -9.11 ± 0.162
Offset (kcal/mol) = -0.785 ± 8.9e-2
ΔG (kcal/mol) = -9.72
-TΔS (kcal/mol) = -0.611

Supplementary Figure 4: Comparison of the binding site in modified BtGH84_TM structures.

(a) BtGH84_TM-**4** conjugate (grey) X-ray crystal structure. The covalent modification is highlighted (pink) and the SA F_o-F_c omit map of **4** is shown as green mesh contoured at 3.5σ. (b) BtGH84_TM-**4** conjugate (grey) X-ray crystal structure with PUGNAc (green) bound to the active site. The covalent modification is highlighted (pink) and the SA F_o-F_c omit map of PUGNAc and **4** is shown as green mesh contoured at 3.5σ. (c) BtGH84_TM-**4** conjugate (grey) X-ray crystal structure with thiamet-G (orange) bound to the active site. The covalent modification is highlighted (pink) and the SA F_o-F_c omit map of thiamet-G and **4** is shown as green mesh contoured at 3.5σ. (d) BtGH84_TM-**8** conjugate (grey) X-ray crystal structure with PUGNAc (green) bound to the active site. The covalent modification is highlighted (blue) and the SA F_o-F_c omit map of PUGNAc and **4** is shown as green mesh contoured at 3.5σ.

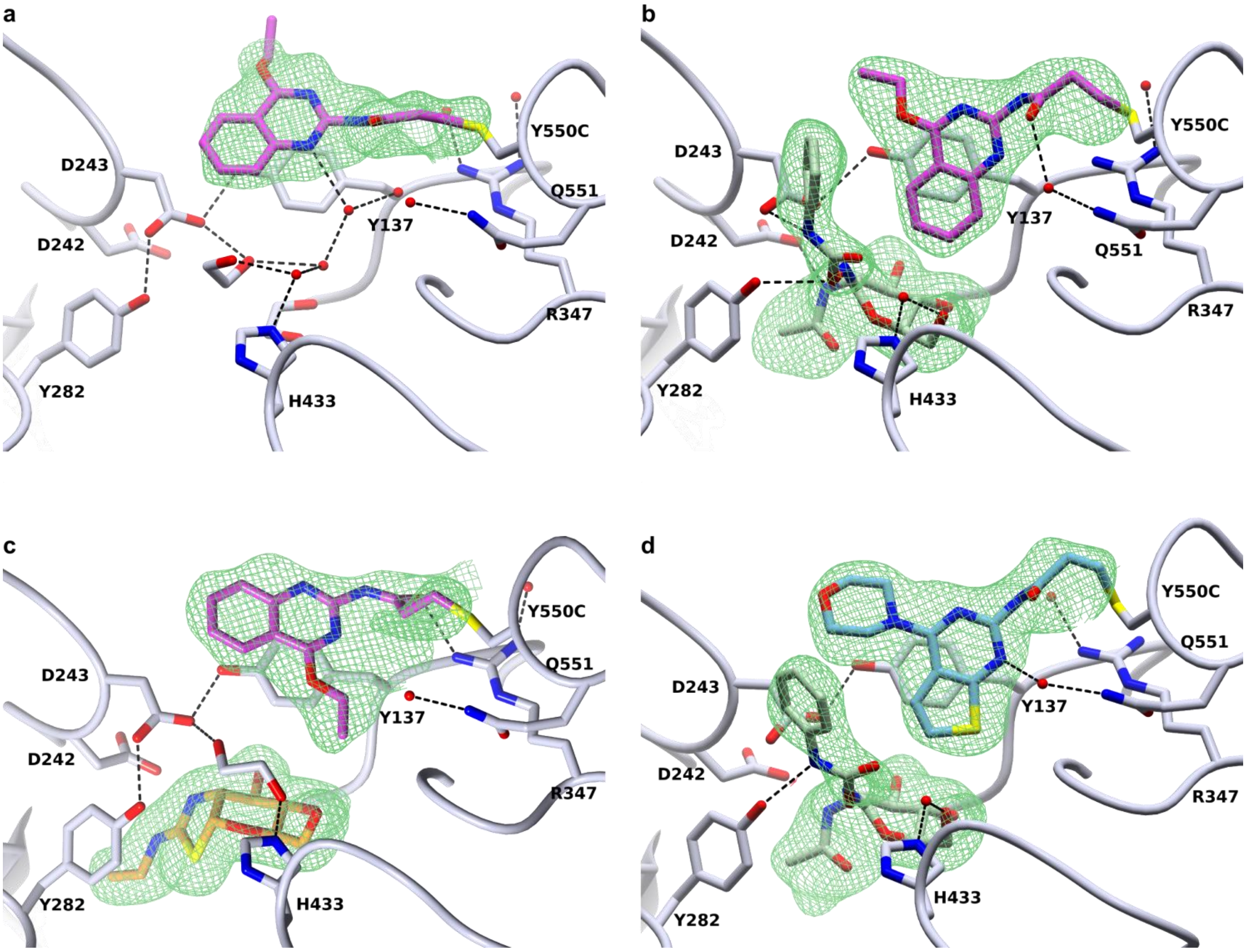

Supplementary Figure 5: Comparison of the binding site in modified BtGH84_TM structures to noncovalent activator binding.

(a-d) Figure as shown in Supplementary Figure 4 with the noncovalent activator **3** (gold) shown as an overlay. The position of **3** is as observed in previously solved structure PDB 4UR9 and overlaid to the covalently modified structures using secondary structure matching of the peptide A chain.

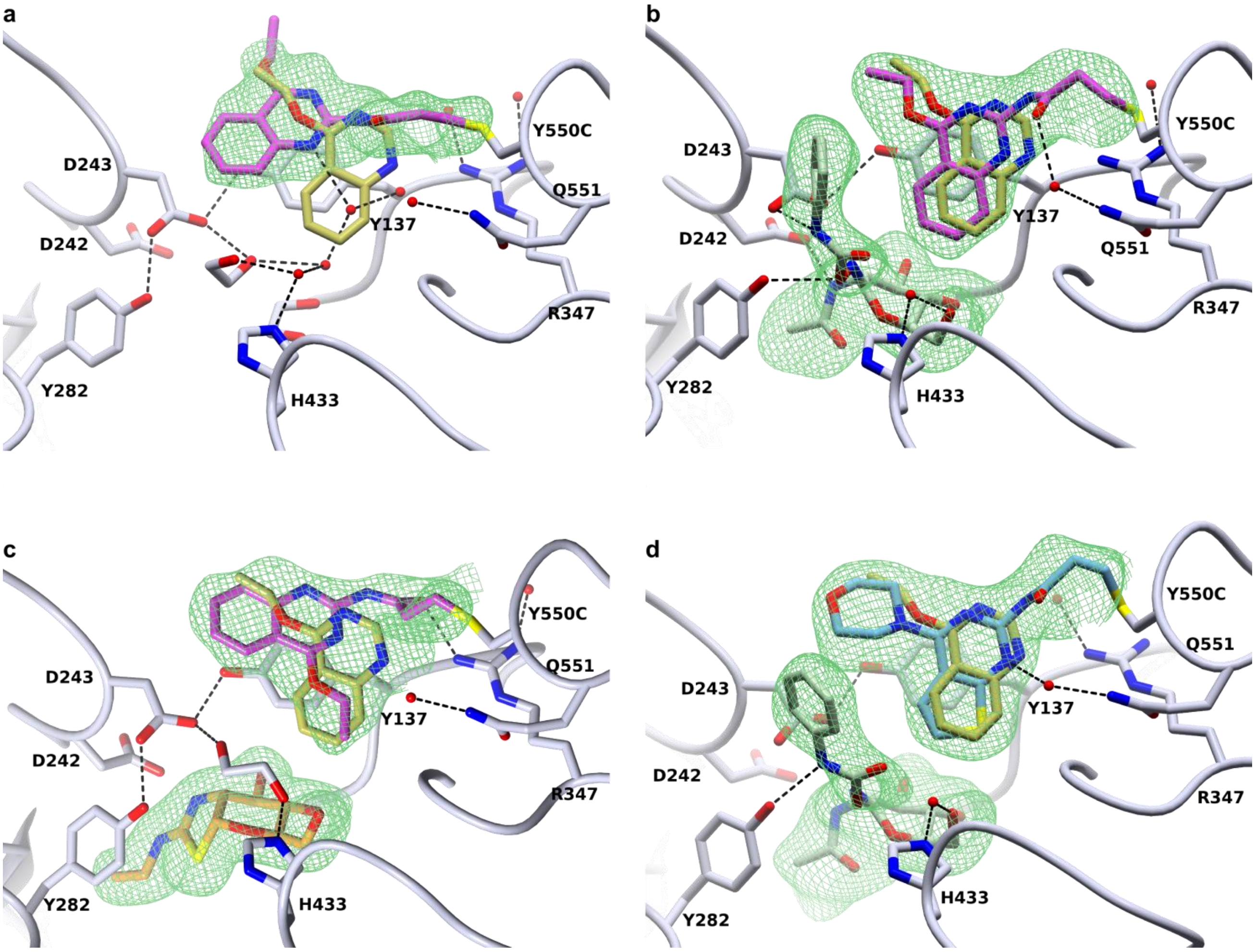

Supplementary Figure 6: Comparison of the full structures of modified BtGH84_TM proteins

(a) BtGH84_TM-**4** conjugate (blue) X-ray crystal structure. The covalent modification is highlighted (pink). (b) BtGH84_TM-**4** conjugate (blue) X-ray crystal structure with PUGNAc (green) bound to the active site. The covalent modification is highlighted (pink). (c) BtGH84_TM-**4** conjugate (blue) X-ray crystal structure with thiamet-G (orange) bound to the active site. The covalent modification is highlighted (pink). (d) BtGH84_TM-**8** conjugate (blue) X-ray crystal structure with PUGNAc (green) bound to the active site.

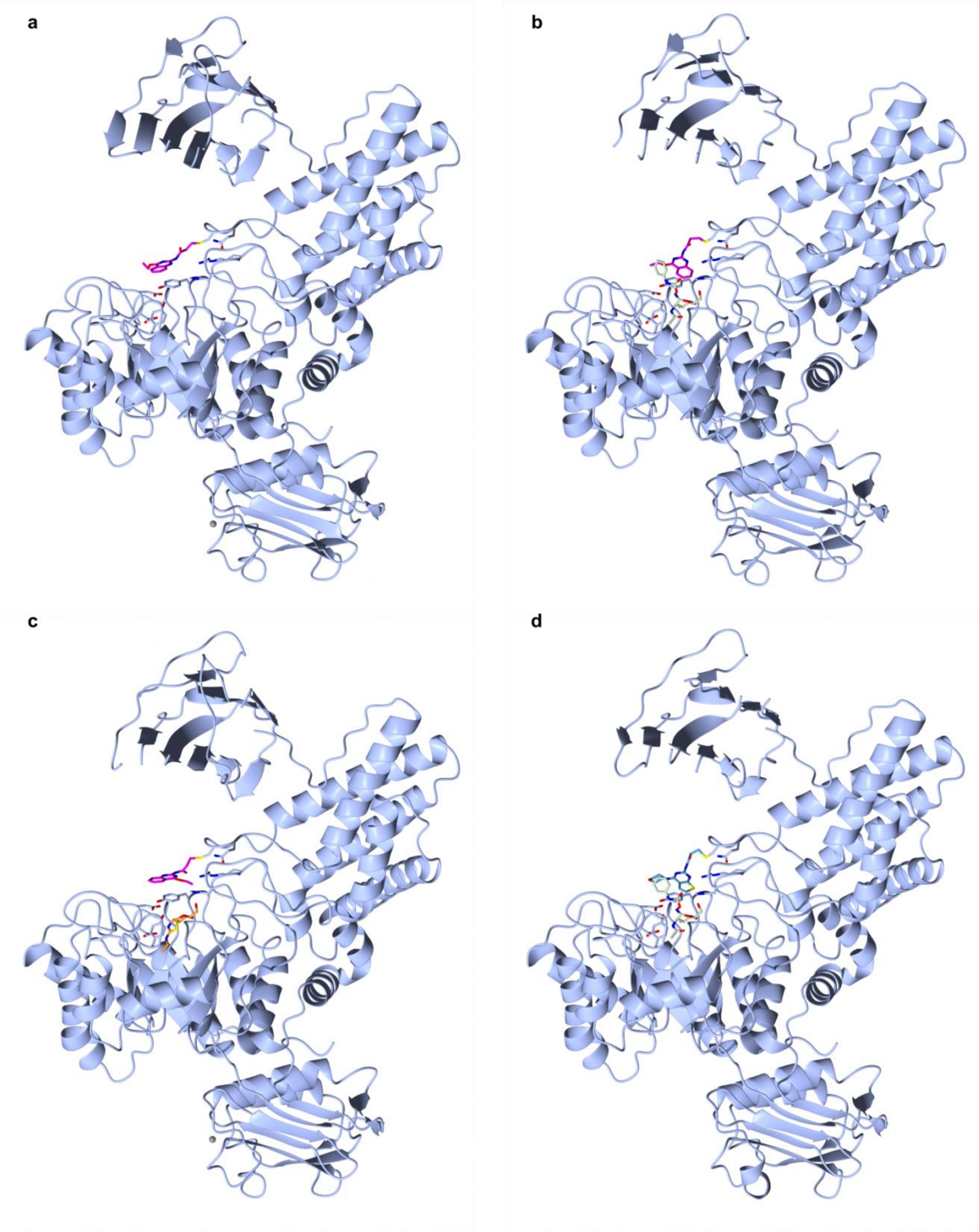

Supplementary Figure 7: Comparison of BtGH84_TM conjugate activity across pH

(a) Activity against buffer pH for BtGH84_TM-fragment conjugates, individually normalised to their respective highest activity. (b) Activity against buffer pH for BtGH84_TM-fragment conjugates, normalised to BtGH84_TM activity at pH 5.5. (c) Activity against buffer pH for BtGH84_TM-fragment conjugates, normalised to BtGH84_TM activity at each pH tested.

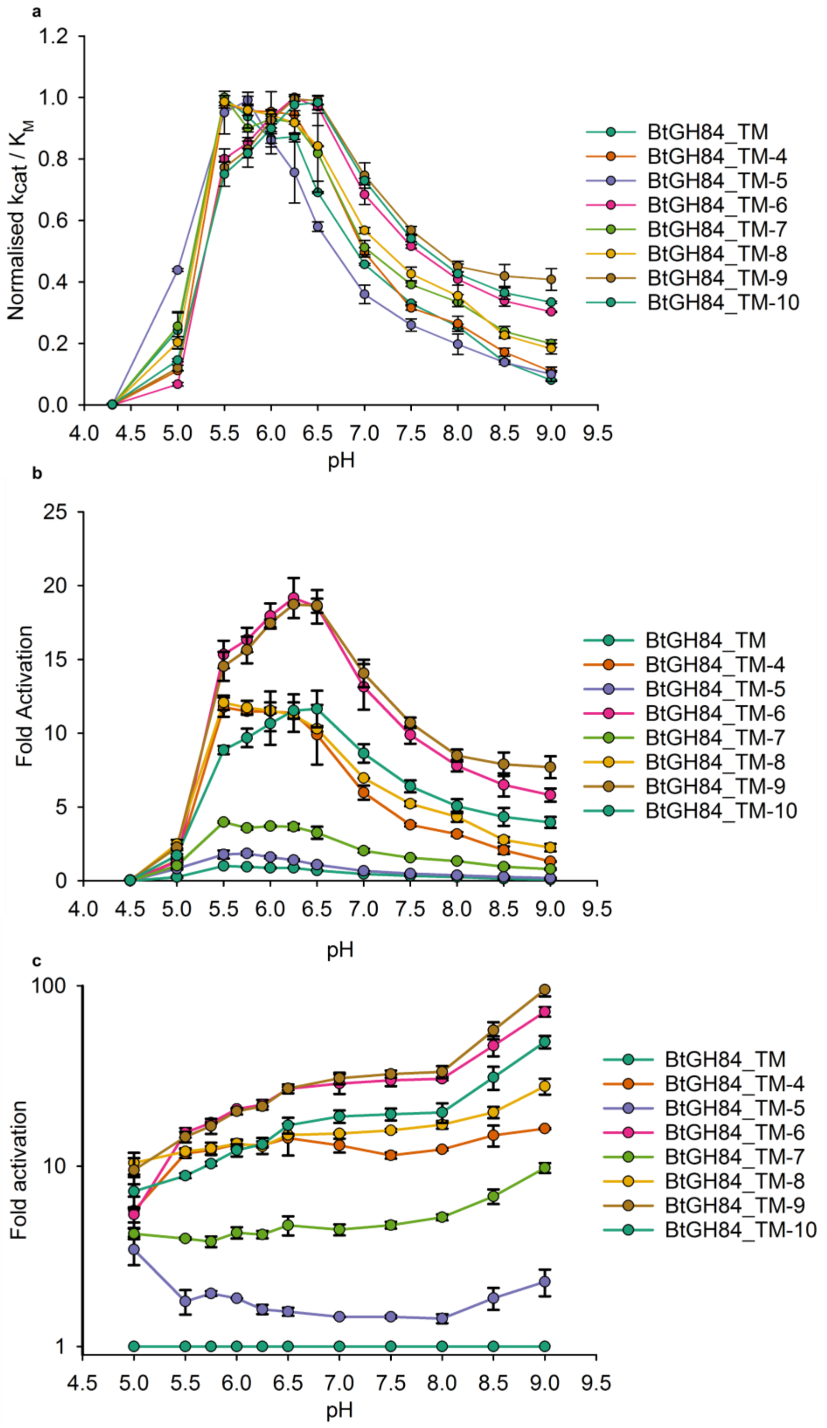

*Supplementary Figure 8: Structure of BtGH84_TM-**4** with substrate 4MU-GlcNAc model*

(a) Chemical structure of 4MU-GlcNAc. (c) Model of 4MU-GlcNAc (yellow) bound to BtGH84_TM-**4** in comparison to PUGNAc (green).

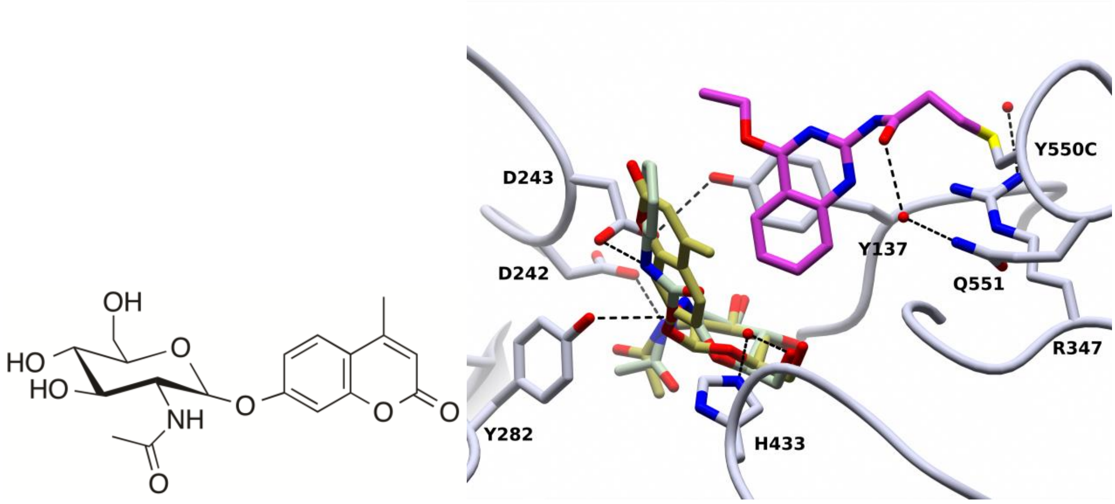

Supplementary Figure 9: Overlays of modified BtGH84_TM structures

(a) Stereo image of BtGH84_TM-**4** conjugate X-ray crystal structures. The covalent modification **4** is shown in dark pink for the complex structure with PUGNAc (green), in grey for the complex structure with thiamet-G (orange) and light pink for the apo structure. (b) Stereo image as in (a) with the additional modification **8** (blue) overlaid.

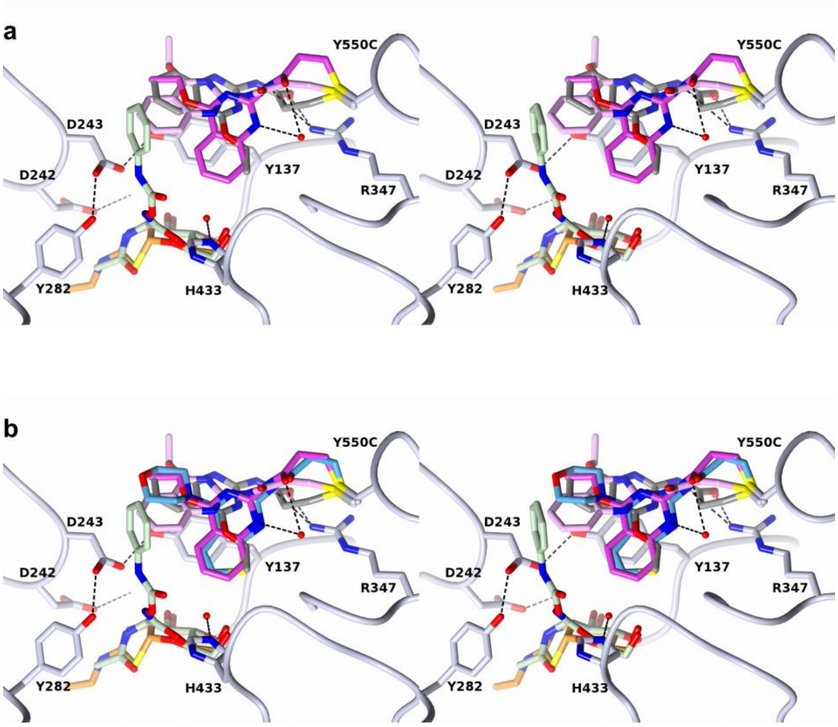

Supplementary Figure 10: Activity is not protein concentration dependent

(a) Michaelis-Menten plots normalised to protein concentration for wild-type BtGH84 at 12.5, 25, 50, and 100 nM shown with green, orange, purple and pink circles respectively. (b) Fold activation of two BtGH84_TM activator conjugates with enzyme concentration at 12.5, 25, 50, and 100 nM shown in green, orange, purple and pink respectively.

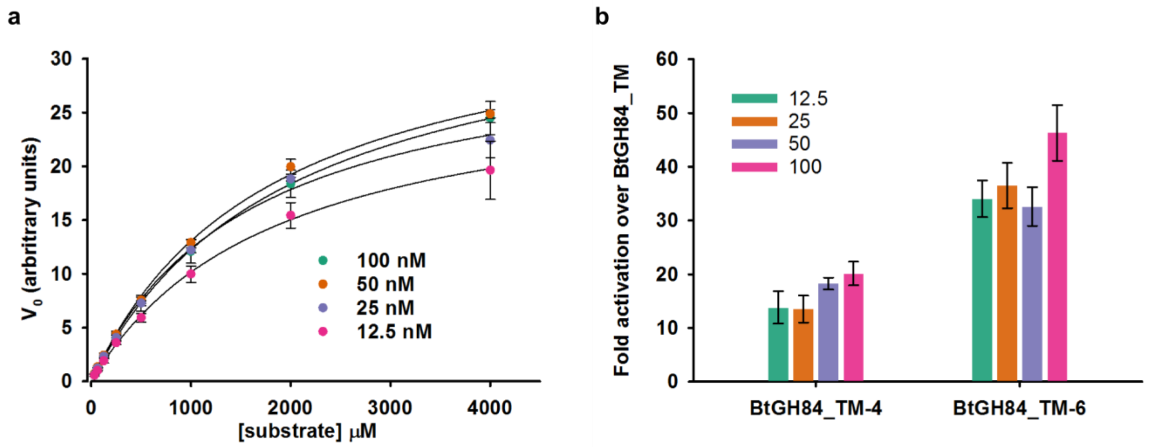

Data

Intact protein mass spectrometry of BtGH84_TM and covalently modified BtGH84_TM

See supplementary methods for information on sample preparation and data collection.

BtGH84_TM

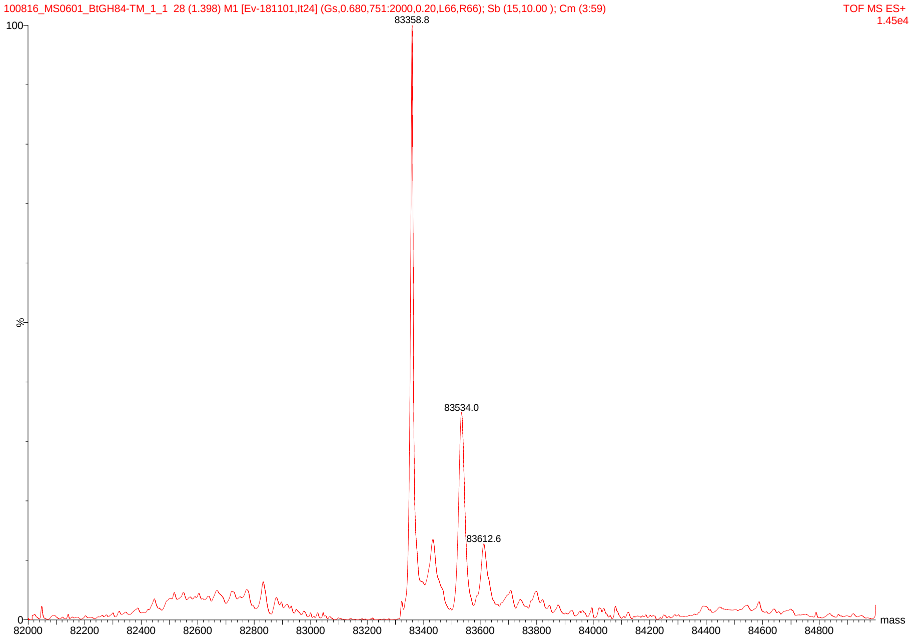

Peaks	83358.8 Da	corrected mass	**83367.1 Da** (expected 83363.4)
	83534.0 Da	corrected mass	83542.3 Da

BtGH84_TM-**4**

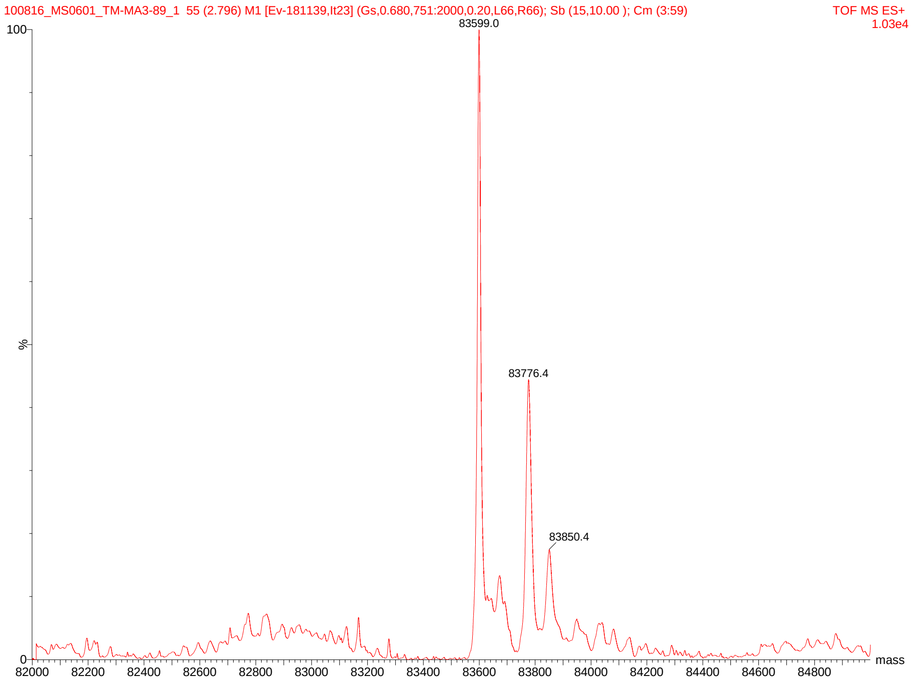

Peaks	83599.0 Da	corrected mass	**83607.3 Da** (expected 83606.4)
	83776.4 Da	corrected mass	83784.7 Da

BtGH84_TM-**5**

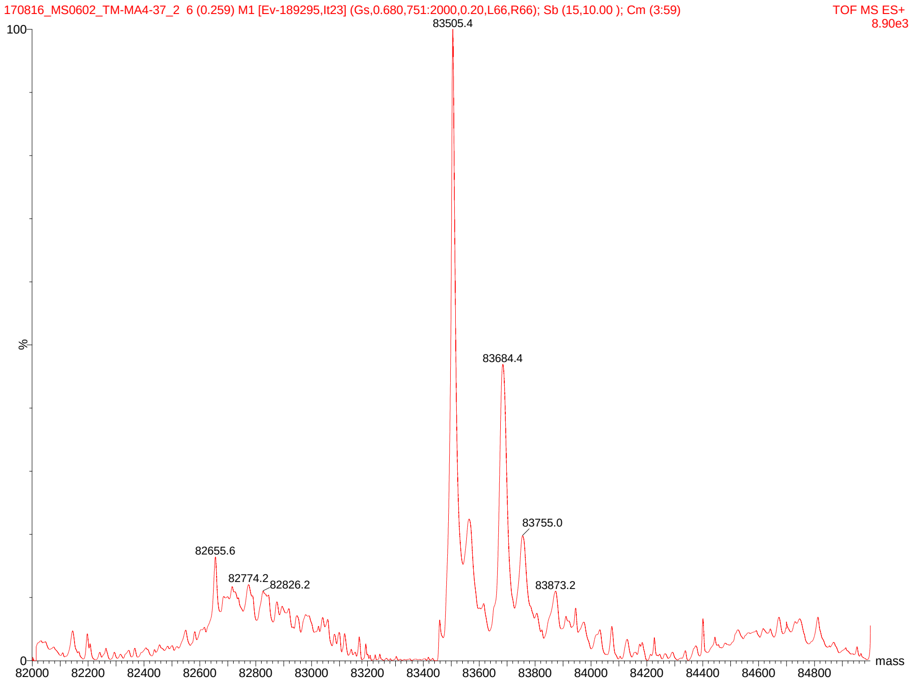

Peaks	83505.4 Da	corrected mass	**83514.2 Da** (expected 83512.2)
	83684.4 Da	corrected mass	83693.2 Da

BtGH84_TM-**6**

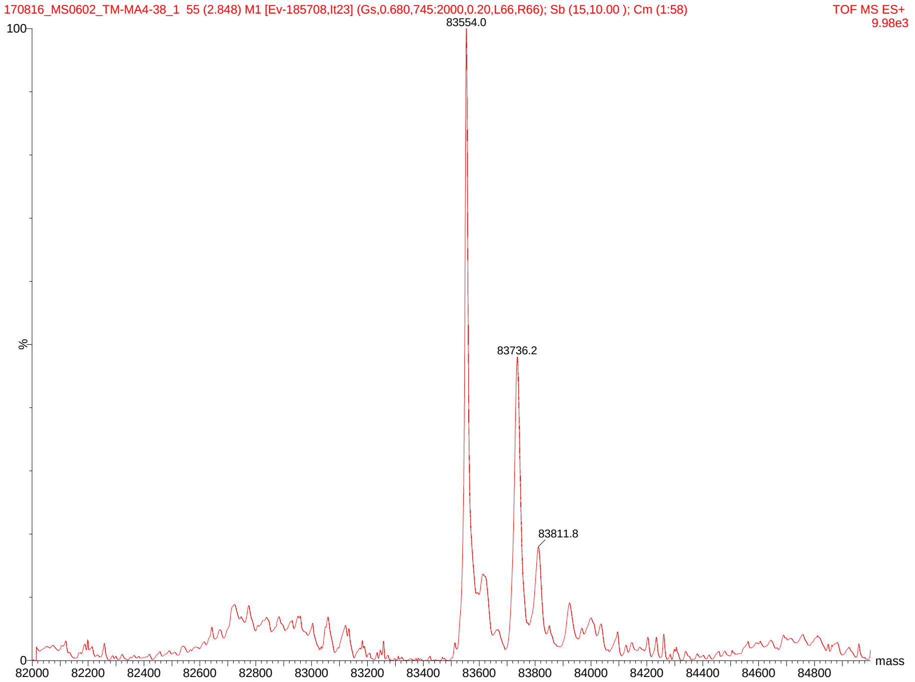

Peaks	83554.0 Da	corrected mass	**83562.8 Da** (expected 83561.6)
	83736.2 Da	corrected mass	83745.0 Da

BtGH84_TM-**7**

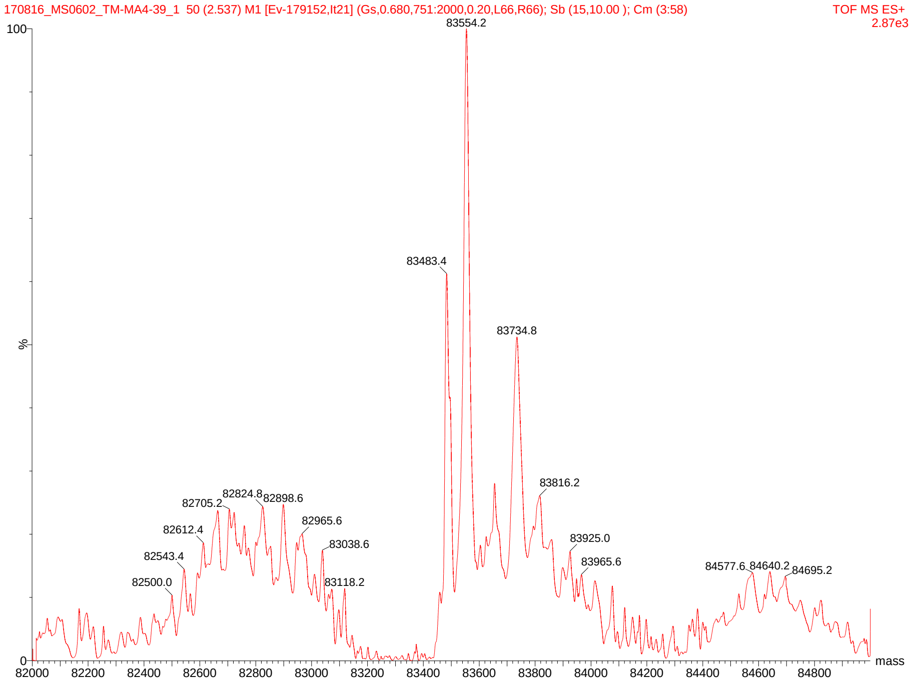

Peaks	83554.2 Da	corrected mass	**83563.0 Da** (expected 83561.6)
	83734.8 Da	corrected mass	83743.6 Da

BtGH84_TM-**8**

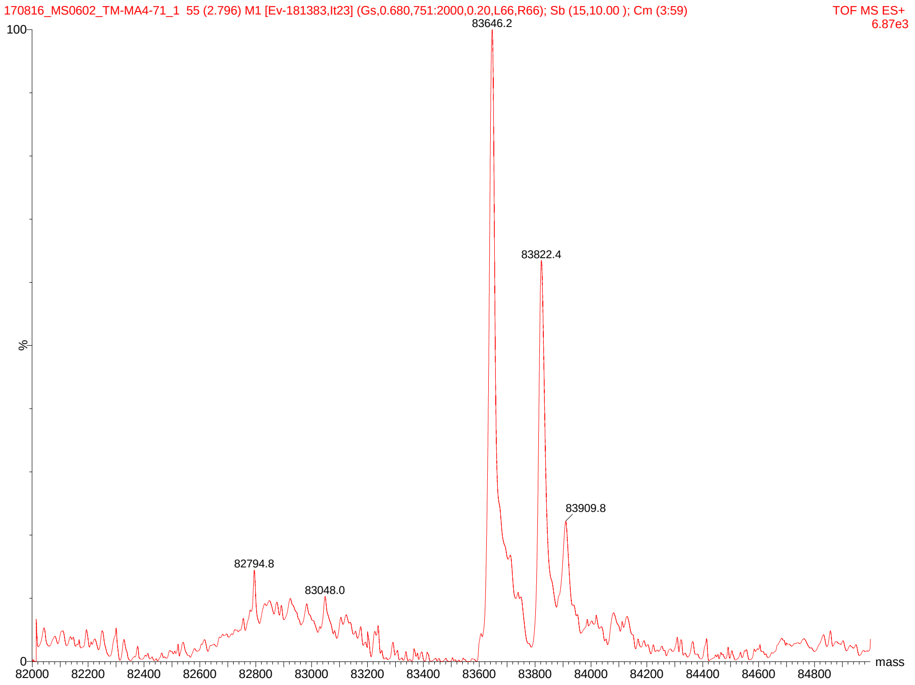

Peaks	83646.2 Da	corrected mass	**83655.0 Da** (expected 83653.7)
	83822.4 Da	corrected mass	83831.2 Da

BtGH84_TM-**9**

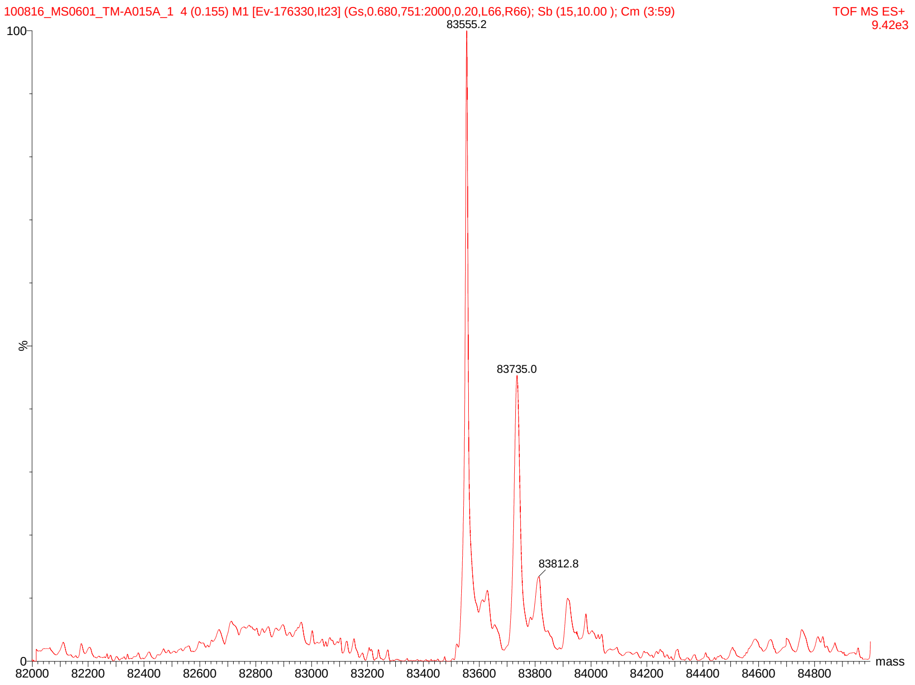

Peaks	83555.2 Da	corrected mass	**83563.5 Da** (expected 83562.6)
	83735.0 Da	corrected mass	83743.3 Da

BtGH84_TM-**10**

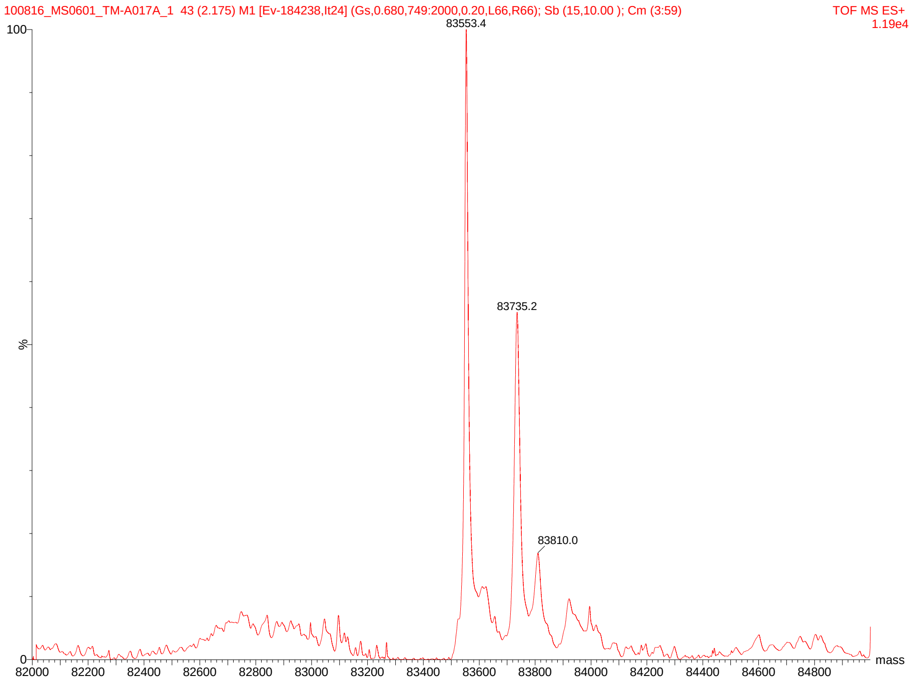

Peaks	83553.4 Da	corrected mass	**83561.7 Da** (expected 83562.6)
	83735.2 Da	corrected mass	83743.5 Da

BtGH84_QM

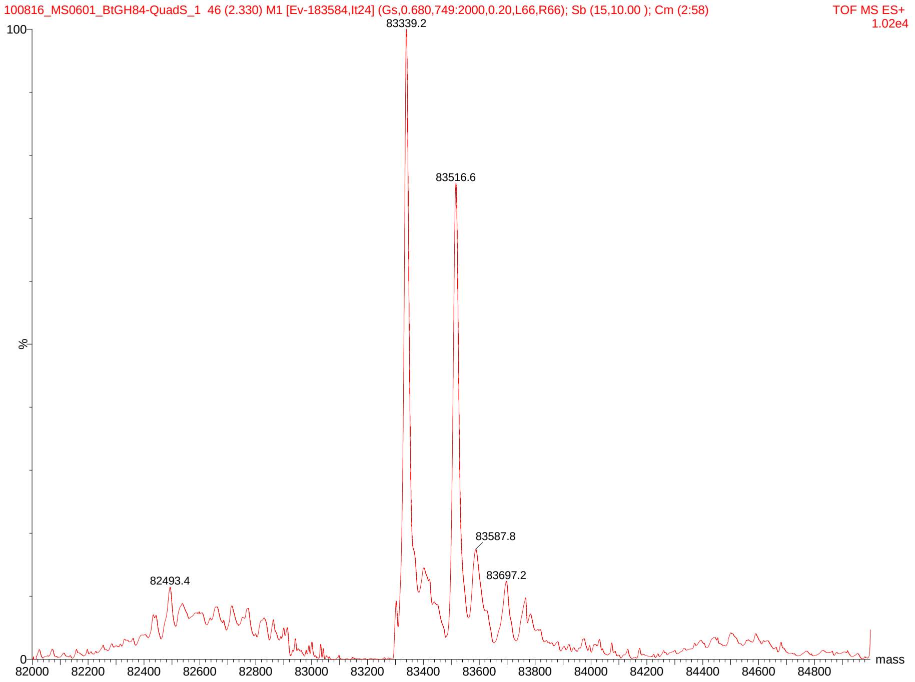

Peaks	83339.2 Da	corrected mass	**83347.5 Da** (expected 83347.6)
	83516.6 Da	corrected mass	83524.9 Da

BtGH84_QM-**4**

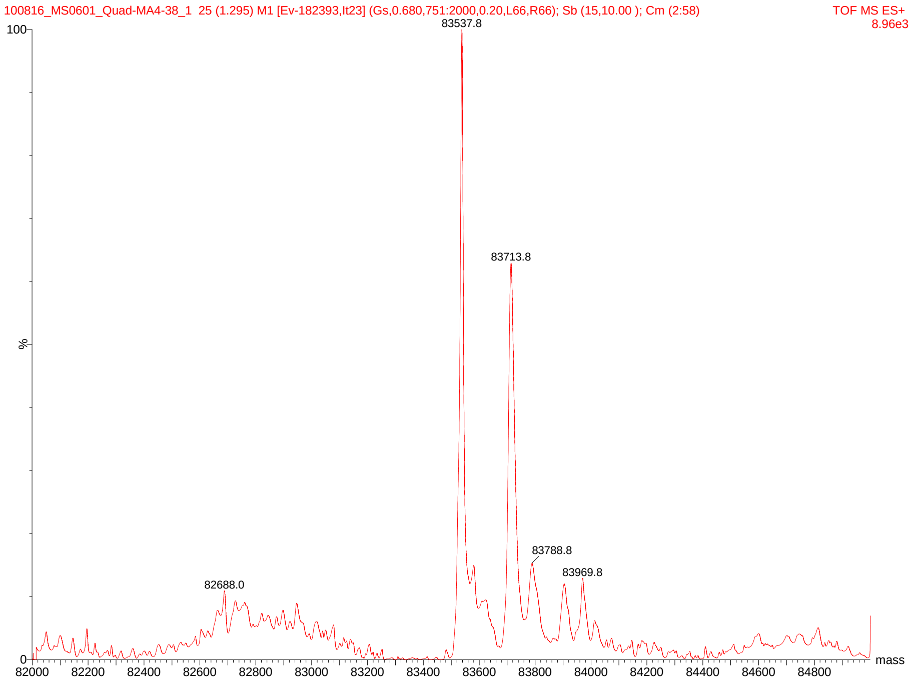

Peaks	83537.8 Da	corrected mass	**83546.1 Da** (expected 83545.8)
	83713.8 Da	corrected mass	83722.1 Da

Synthesis and compound characterisation

Description of synthetic strategy – Schemes S1 – S5

To generate our covalent activators, we developed a synthetic approach using the Curtius rearrangement reaction to construct the 2-amino-quinazoline structure. The synthetic route started from the commercially available isatoic anhydride, which was first amidated to the corresponding amide **S1** with NH_3 in MeOH and then cyclised with diethyl oxalate to give quinazolin-4-one **S2**. After chlorination of **S2**, to give **S3**, substitution at the 4-position of quinazoli-4-one gave **S4**. Hydrolysis of **S4** with 2M NaOH gave the corresponding carboxylic acid **S5**, which upon Curtius rearrangement formed Boc-carbamate **S6**. After deprotection of **S6** with TFA, acrylamidation of **S7** afforded the final compound **4** (Scheme S1).

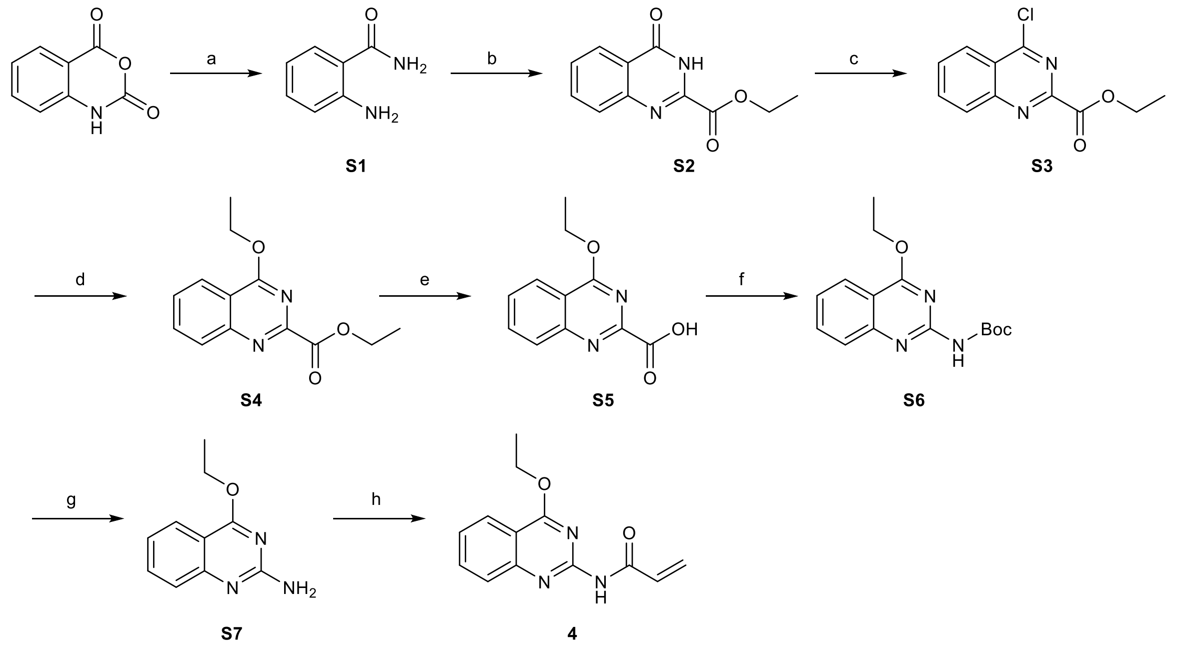

Reagents and conditions: (a) NH_3 in MeOH; (b) diethyl oxalate, 180 °C (2 steps, 43%); (c) $SOCl_2$, (cat)DMF, $CHCl_3$, reflux; (d) EtONa, EtOH, rt (2 steps, 71%); (e) 2M NaOH, EtOH, rt; (f) DPPA, Et_3N, tBuOH, reflux (2 steps, 48%); (g) TFA, CH_2Cl_2, rt (quant); (h) Acryloyl chloride, KHMDS, THF, 0 °C (36%).

(Scheme S1)

For compounds **5**-**7**, commercially available heterocyclic amines were amidated to give the corresponding acryl amide (Scheme S2).

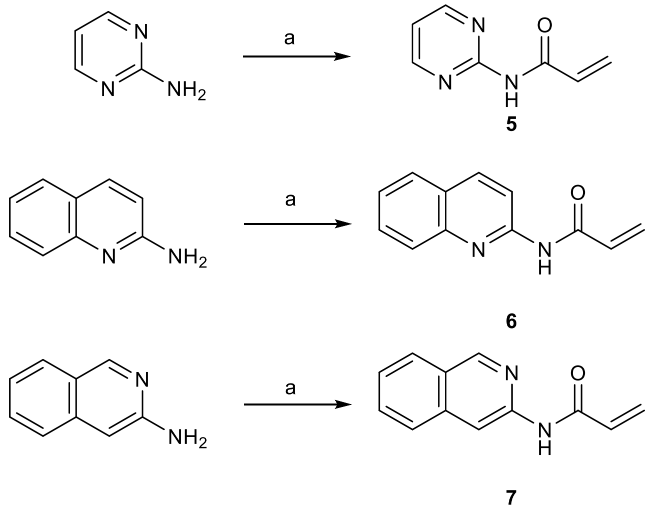

Reagents and conditions: (a) Acryloyl chloride, KHMDS, THF, 0 °C (**5**: 6%, **6**, 16%, **7**, 15%).

(Scheme S2)

Compound **8** was synthesized according to Scheme S3. The coupling reaction of between 4-(2-chlorothieno[2,3-d]pyrimidin-4-yl)morpholine and Boc-NH_2 gave **S8**. Treatment with TFA gave the amine **S9**. Finally, amidation of **S9** with acryloyl chloride and Et_3N afforded **8** (Scheme S3).

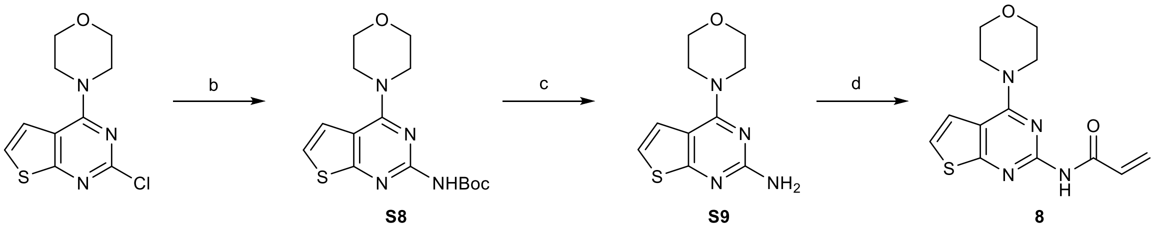

Reagents and conditions: (a) $Pd(OAc)_2$, Cs_2CO_3, BippyPhos, $BocNH_2$, dioxane, 110 °C (86%); (b) TFA, CH_2Cl_2, rt (72%); (c) Acryloyl chloride, Et_3N, CH_2Cl_2-DMF, rt (41%).

(Scheme S3)

Compound **9** was synthesised according to Scheme S4. 3-Aminopyridine-4-carboxylic acid was converted into the corresponding aldehyde **S11** before Friedlander reaction gave naphthyridine **S12.** Curtius rearrangement gave **S13** before amidation and deprotection gave Michael acceptor **9**.

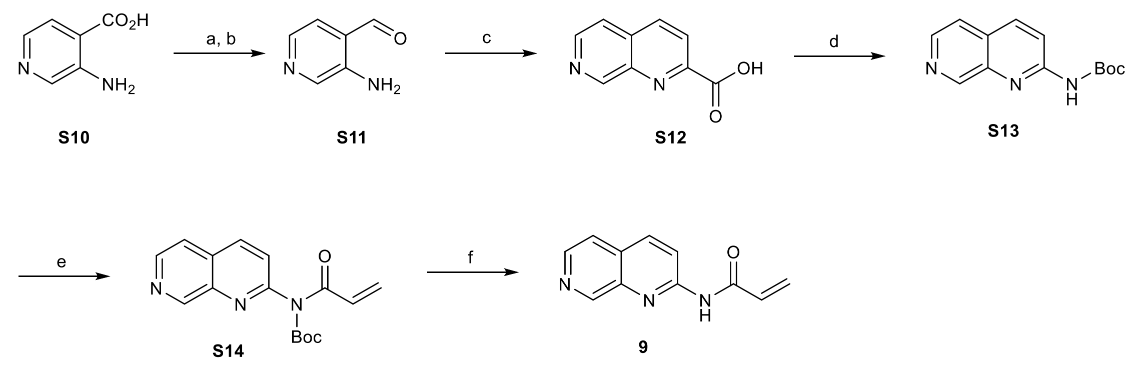

Reagents and conditions: (a) $LiAlH_4$, THF, reflux, 66%; (b) MnO_2, $CHCl_3$, 64%; (c) sodium pyruvate, NaOH, H_2O; (d) DPPA, Et_3N, tBuOH, toluene, 70 °C (2 steps, 22%); (e) Acryloyl chloride, Et_3N, CH_2Cl_2;(f) TFA, CH_2Cl_2, rt (8%, 2 steps).

(Scheme S4)

Analogously, compound **10** was synthesised as shown in Scheme S5. 1,6-Naphthyridine-2-carboxylic acid underwent Curtius rearrangement to give **S15** before amidation and deprotection gave Michael acceptor **10**.

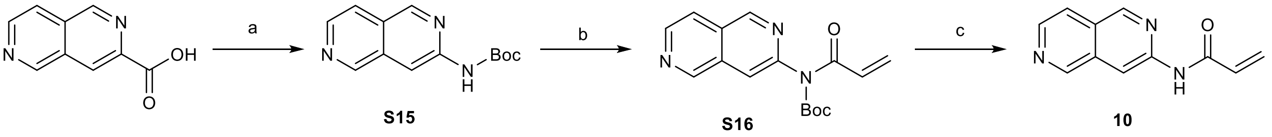

Reagents and conditions: (a) DPPA, Et_3N, tBuOH, toluene, 80 °C (48%); (b) Acryloyl chloride, Et_3N, CH_2Cl_2; (c) TFA, CH_2Cl_2, rt (7%, 2 steps).

(Scheme S5)

Experimental Details

Ethyl 4-Oxo-3,4-dihydroquinazoline-2-carboxylate S2

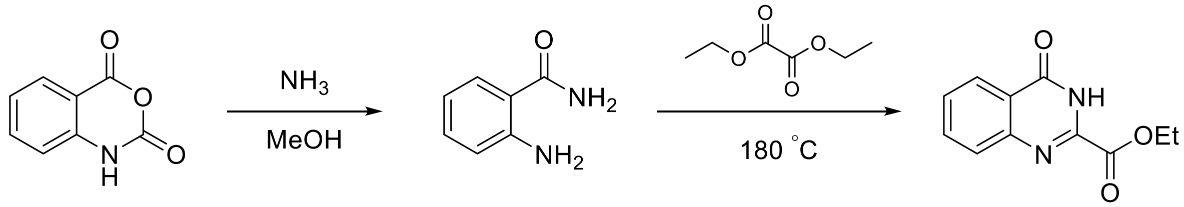

S1 **S2**

A mixture of isatoic anhydride (5.0 g, 30.7 mmol, 1.0 eq.) and 35% $NH_4OH_{(aq)}$ (14.6 mL, 307 mmol, 10.0 eq.) was stirred at rt for 1 h. The solvent was evaporated under reduced pressure to afford amide **S1** (4.18 g, quant) as a brown solid, which was used in the next step without purification. A mixture of amide **S1** (4.18 g, 30.7 mmol, 1.0 eq.) and diethyl oxalate (8.3 mL, 61.4 mmol, 2.0 eq.) was heated to 170 °C for 6 h. The mixture was cooled to rt and diluted with EtOH (10 mL) and hexane (10 mL). The resultant precipitate was collected by filtration and washed thoroughly with EtOH to give ester **S2** (2.87 g, 43% over 2 steps) as a yellow solid, mp 162-165 °C (lit.,[1] 184-185 °C), R_F (1:1 hexane-EtOAc) 0.5; IR (ATR) 3068 (NH), 1675 (C=O), 1304, 768 cm^{-1}; ^{1}H NMR (400 MHz, DMSO-*d*$_6$) δ 8.18 (dd, *J* = 8.0, 1.0 Hz, 1H, Ar), 7.89 (ddd, *J* = 8.0, 7.0, 1.0 Hz, 1H, Ar), 7.83 (br d, *J* = 8.0 Hz, 1H, Ar), 7.64 (ddd, *J* = 8.0, 7.0, 1.0 Hz, 1H, Ar), 4.38 (q, *J* = 7.0 Hz, 2H, CH_2), 1.35 (t, *J* = 7.0 Hz, 1H, CH_3); ^{13}C NMR (100.6 MHz, DMSO-*d*$_6$) δ 161.0 (C=O), 160.1 (C=O), 147.3 (*ipso*-Ar), 143.4 (*ipso*-Ar), 134.8 (Ar), 128.7 (Ar), 128.4 (Ar), 126.1 (Ar), 123.0 (*ipso*-Ar), 62.7 (CH_2), 13.9 (CH_3); MS (ESI) *m/z* 241 [(M + Na)$^+$, 100]; HRMS (ESI) *m/z* calcd for $C_{11}H_{10}N_2O_3$ (M + Na)$^+$ 241.0584, found 241.0589 (–1.9 ppm error). Spectroscopic data consistent with those reported in the literature.[1]

Lab Book Reference: MA 3-2 & 3-4

Ethyl 4-Ethoxyquinazoline-2-carboxylate S4

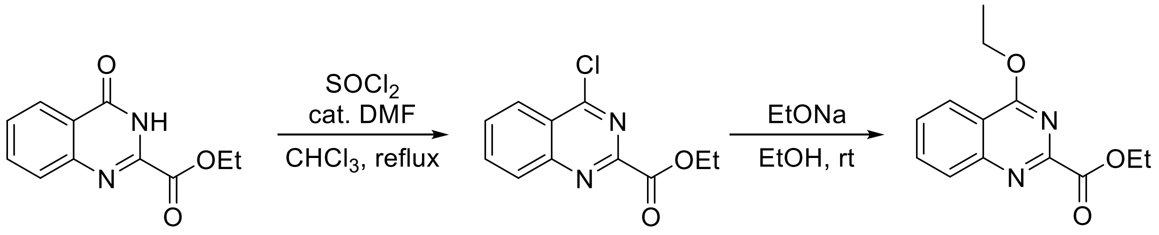

S2 **S3** **S4**

Thionyl chloride (11.5 mL, 158 mmol, 12 eq.) was added dropwise to a stirred solution of ester **S2** (2.87 g, 13.2 mmol, 1.0 eq.) and *N*,*N*-dimethylformamide (14 drops) in $CHCl_3$ (28 mL) at 0 °C. The resulting solution was stirred and heated at reflux for 3.5 h. The solvent was evaporated under reduced pressure to afford the chloride **S3** (3.12 g, quant.)[2] as a brown solid, which was used in the next step without purification. A mixture of the chloride **S3** (3.12 g, 13.2 mmol, 1.0 eq.) and EtONa (9.85 mL 21% solution in EtOH, 26.4 mmol, 2.0 eq.) in EtOH (50 mL) was stirred at rt to for 1 h. Then, the mixture was diluted with hexane (10 mL) and the resultant precipitate was collected by filtration and washed thoroughly with EtOH to give the crude product. Purification by flash column chromatography on silica with 4:1 hexane-EtOAc as eluent gave ethyl ether **S4** (1.74 g, 71%) as a yellow solid, mp 58-60 °C, R_F (1:1 hexane-EtOAc) 0.5; IR (ATR) 2964, 1724 (C=O), 1572, 1370, 1103, 774 cm^{-1}; ^{1}H NMR (400 MHz, DMSO-d_6) δ 8.23 (br d, J = 8.5 Hz, 1H, Ar), 8.13 (br d, J = 8.5 Hz, 1H, Ar), 7.92-7.87 (m, 1H, Ar), 7.66 (br dd, J = 8.0, 7.0 Hz, 1H, Ar), 4.76 (q, J = 7.0 Hz, 2H, CH_2), 4.55 (q, J = 7.0 Hz, 2H, CH_2), 1.54 (t, J = 7.0 Hz, 3H, CH_3), 1.48 (dt, J = 7.0, 0.5 Hz, 3H, CH_3); ^{13}C NMR (100.6 MHz, DMSO-d_6) δ 166.9 (C=O), 163.6 (C=O), 152.2 (*ipso*-Ar), 150.0 (*ipso*-Ar), 134.8 (Ar), 129.3 (Ar), 128.3 (Ar), 123.4 (Ar), 115.9 (*ipso*-Ar), 63.5 (CH_2), 61.7 (CH_2), 14.1 (CH_3); MS (ESI) *m/z* 269 [$(M + Na)^+$, 100], 247 [$(M + H)^+$, 40]; HRMS (ESI) *m/z* calcd for $C_{13}H_{14}N_2O_3$ $(M + Na)^+$ 269.0897, found 269.0893 (+1.4 ppm error).

Lab Book Reference: MA 3-7 & 3-12

4-Ethoxyquinazoline-2-carboxylic acid S5

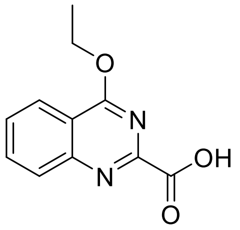

S5

2 M $NaOH_{(aq)}$ (4.5 mL, 9.0 mmol, 5.0 eq.) was added dropwise to a stirred solution of ethyl ether **S4** (450 mg, 1.83 mmol, 1.0 eq.) in EtOH (4.5 mL) at rt. The resulting solution was stirred at rt for 2 h. Then, the mixture was neutralised with 2 M $HCl_{(aq)}$ (4.5 mL) and the two layers were separated. The aqueous layer was extracted with CH_2Cl_2 (3 × 30 mL). The combined organic layers were dried ($MgSO_4$) and evaporated under reduced pressure to give carboxylic acid **S5** (431 mg, quant) as an orange solid, 123-125 °C, R_F (1:1 hexane-EtOAc) 0.1; IR (ATR) 3363, 3150, 1619 (C=O), 1472 cm^{-1}; ^{1}H NMR (400 MHz, DMSO-d_6) δ 8.22-8.20 (m, 1H, Ar), 8.04-8.03 (m, 2H, Ar), 7.81-7.77 (m, 1H, Ar), 4.67 (q, J = 7.0 Hz, 2H, CH_2), 1.47 (t, J = 7.0 Hz, 3H, CH_3); ^{13}C NMR (100.6 MHz, DMSO-d_6) δ 166.9 (C=O), 160.0 (*ipso*-Ar), 153.2 (*ipso*-Ar), 145.6 (*ipso*-Ar), 133.5 (Ar), 124.0 (Ar), 123.3 (Ar), 121.2 (Ar), 111.0 (*ipso*-Ar), 62.0 (CH_2), 14.3 (CH_3); MS (ESI) *m/z* 219 [(M + H)$^+$, 100]; HRMS (ESI) *m/z* calcd for $C_{11}H_{10}N_2O_3$ (M + H)$^+$ 219.0764, found 219.0758 (+1.2 ppm error).

Lab Book Reference: MA 3-15

***tert*-Butyl (4-Ethoxyquinazolin-2-yl)carbamate S6**

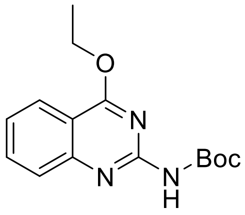

S6

Diphenylphosphoryl azide (693 μL, 3.2 mmol, 1.1 eq.) was added dropwise to a stirred solution of carboxylic acid **S5** (637 mg, 2.9 mmol, 1.0 eq.) and Et_3N (488 μL, 3.5 mmol, 1.2 eq.) in ^{t}BuOH (14 mL) at rt. The resulting solution was stirred and heated at reflux for 12 h. The solvent was evaporated under reduced pressure to afford the crude product. Purification by flash column chromatography on silica with 8:1 hexane-EtOAc as eluent gave carbamate **S6** (403 mg, 48%) as a white solid, mp 68-70°C, R_F (1:1 hexane-EtOAc) 0.7; IR (ATR) 2979, 1744 (C=O), 1576, 1142, 727 cm^{-1}; ^1H NMR (400 MHz, $CDCl_3$) δ 8.04 (br d, J = 8.0 Hz, 1H, Ar), 7.83 (d, J = 8.0 Hz, 1H, Ar), 7.71 (br dd, J = 8.0, 7.0 Hz, 1H, Ar), 7.31 (br dd, J = 8.0, 7.0 Hz, 1H, Ar), 7.30 (br s, 1H, NH), 4.57 (q, J = 7.0 Hz, 2H, CH_2), 1.55 (s, 9H, C*Me₃*), 1.49 (t, 3H, J = 7.0 Hz, CH_3); ^{13}C NMR (100.6 MHz, $CDCl_3$) δ 167.8 (C=O), 153.5 (N-*C*=N), 152.1 (*ipso*-Ar), 150.6 (*ipso*-Ar), 133.8 (Ar), 126.9 (Ar), 124.8 (Ar), 123.5 (Ar), 113.9 (*ipso*-Ar), 81.2 (*C*Me_3), 63.3 (CH_2), 28.3 (CMe_3), 14.4 (CH_3); MS (ESI) *m/z* 290 [(M + H)$^+$, 100]; HRMS (ESI) *m/z* calcd for $C_{15}H_{19}N_3O_3$ (M + H)$^+$ 290.1499, found 290.1504 (–1.5 ppm error).

Lab Book Reference: MA 3-20

4-Ethoxyquinazolin-2-amine S7

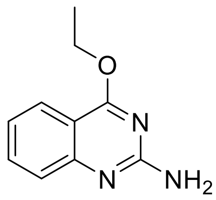

S7

TFA (6.0 mL, 78 mmol, 55 eq.) was added to a stirred solution of carbamate **S6** (466 mg, 1.6 mmol, 1.0 eq.) in CH_2Cl_2 (6 mL) at rt. The resulting solution was stirred at rt for 4 h. The solvent was evaporated under reduced pressure. Then, $NH_4OH_{(aq)}$ (10 mL) was added and the mixture was extracted with CH_2Cl_2 (3 × 20 mL). The combined organic layers were dried ($MgSO_4$) and evaporated under reduced pressure to give the crude product. Purification by flash column chromatography on silica with 200:9:1 CH_2Cl_2-MeOH-$NH_4OH_{(aq)}$ as eluent gave amine **S7** (304 mg, 100%) as a white solid, mp 168-170 °C, R_F (CH_2Cl_2:MeOH:$NH_4OH_{(aq)}$ 100:9:1) 0.3; IR (ATR) 3342 (NH), 3000, 1614, 1336, 1130 cm^{-1}; ^1H NMR (400 MHz, DMSO-*d*$_6$) δ 8.04 (dd, *J* = 8.0, 1.0 Hz, 1H, Ar), 7.89 (ddd, *J* = 8.0, 8.0, 1.0 Hz, 1H, Ar), 7.56 (br d, *J* = 8.0 Hz, 1H, Ar), 7.46 (ddd, *J* = 8.0, 8.0 1.0 Hz, 1H, Ar), 4.64 (q, *J* = 7.0 Hz, 2H, CH_2), 1.46 (t, *J* = 7.0 Hz, 3H, CH_3); ^{13}C NMR (100.6 MHz, DMSO-*d*$_6$) δ 168.5 (N-*C*=N), 155.7 (*ipso*-Ar), 141.3 (*ipso*-Ar), 136.5 (Ar), 124.9 (Ar), 124.8 (Ar), 117.1 (Ar), 110.5 (*ipso*-Ar), 65.0 (CH_2), 14.0 (CH_3); MS (ESI) *m/z* 190 [(M + H)$^+$]; HRMS *m/z* calcd for $C_{10}H_{11}N_3O$ (M + H)$^+$ 190.0975, found 190.0970 (+2.9 ppm error).

Lab Book Reference: MA 3-25

***N*-(4-Ethoxyquinazolin-2-yl)acrylamide 4**

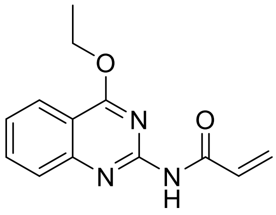

4

Acryloyl chloride (26 μL, 0.32 mmol, 1.0 eq.) was added dropwise to a stirred solution of amine **S7** (60 mg, 0.32 mmol, 1.0 eq.) and KHMDS (636 μL of a 0.5 M solution in toluene, 0.32 mmol, 1.0 eq.) in THF (3.2 mL) at 0 °C under Ar. The resulting solution was stirred at 0 °C for 3 h. Then, saturated $NaHCO_{3(aq)}$ (5 mL) was added and the mixture was extracted with EtOAc (3 × 10 mL). The combined organic layers were dried ($MgSO_4$) and evaporated under reduced pressure to give the crude product. Purification by flash column chromatography on silica with 3:1 hexane-EtOAc as eluent gave carbamate **4** (28 mg, 36%) as a white solid, mp 178-180 °C, R_F (1:1 hexane-EtOAc) 0.3; IR (ATR) 2964, 1696 (C=O), 1577, 1323, 1196, 1016 765 cm^{-1}; ^1H NMR (400 MHz, $CDCl_3$) δ 8.15 (br s, 1H, NH), 8.07 (ddd, J = 8.0, 1.0, 1.0 Hz, 1H, Ar), 7.77-7.12 (m, 2H, Ar), 7.40 (dd, J = 8.0, 8.0 Hz, 1H, Ar), 7.23-7.12 (m, 1H, =CH), 6.54 (dd, J = 17.0, 1.5 Hz, 1H, =CH), 5.85 (dd, J = 10.5, 1.5 Hz, 1H, =CH), 4.60 (q, J = 7.0 Hz, 2H, CH_2), 1.50 (t, J = 7.0 Hz, 3H, CH_3); ^{13}C NMR (100.6 MHz, $CDCl_3$) δ 168.0 (C=O), 165.2 (N-*C*=N), 153.3 (*ipso*-Ar), 151.8 (*ipso*-Ar), 134.2 (Ar), 130.7 (Ar), 129.4 (=CH_2), 126.6 (Ar), 125.3 (Ar), 123.8 (=CH), 114.0 (*ipso*-Ar), 63.7 (CH_2), 14.3 (CH_3); MS (ESI) *m/z* 244 [$(M + H)^+$, 100]; HRMS (ESI) *m/z* calcd for $C_{13}H_{13}N_3O_2$ $(M + H)^+$ 244.1081, found 244.1077 (+1.9 ppm error).

Lab Book Reference: MA 3-89

N-(Pyrimidin-2-yl)acrylamide 5

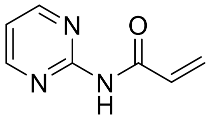

5

Acryloyl chloride (171 μL, 2.1 mmol, 1.0 eq.) was added dropwise to a stirred solution of 2-aminopyrimidine (200 mg, 2.1 mmol, 1.0 eq.) and KHMDS (4.21 mL of a 0.5 M solution in toluene, 2.1 mmol, 1.0 eq.) in THF (21 mL) at 0 °C under Ar. The resulting solution was stirred at 0 °C for 2 h. Then, saturated $NaHCO_{3(aq)}$ (20 mL) was added and the mixture was extracted with EtOAc (3 × 30 mL). The combined organic layers were dried ($MgSO_4$) and evaporated under reduced pressure to give the crude product. Purification by flash column chromatography on silica with 40:1 CH_2Cl_2-MeOH as eluent gave carbamate **5** (17 mg, 6%) as a white solid, mp 92-95 °C (lit.,[3] 125-126 °C), R_F (10:1 CH_2Cl_2-MeOH) 0.4; IR (ATR) 1684 (C=O), 1669, 1575 cm^{-1}; ^{1}H NMR (400 MHz, $CDCl_3$) δ 9.40 (br s, 1H, NH), 8.65 (d, J = 5.0 Hz, 2H, Py), 7.03 (t, J = 5.0 Hz, 1H, Py), 6.92 (br dd, J = 17.0, 10.5 Hz, 1H, =CH), 6.54 (dd, J = 17.0, 1.0 Hz, 1H, =CH), 5.85 (dd, J = 10.5, 1.0 Hz, 1H, =CH); ^{13}C NMR (100.6 MHz, $CDCl_3$) δ 164.9 (C=O), 158.5 (Py), 157.7 (*ipso*-Ar), 130.6 (Py), 129.7 (=CH_2), 116.5 (=CH); MS (ESI) *m/z* 172 [(M + Na)$^+$, 100]; HRMS (ESI) *m/z* calcd for $C_7H_7N_3O_2$ (M + Na)$^+$ 172.0481, found 172.0473 (+3.8 ppm error). Spectroscopic data consistent with those reported in the literature.[3]

Lab Book Reference: MA 4-37

***N*-(Quinolin-2-yl)acrylamide 6**

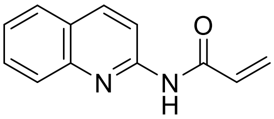

6

Acryloyl chloride (34 μL, 0.42 mmol, 1.0 eq.) was added dropwise to a stirred solution of 2-aminoquiniline (60 mg, 0.42 mmol, 1.0 eq.) and KHMDS (833 μL of a 0.5 M solution in toluene, 0.42 mmol, 4.0 eq.) in THF (4.2 mL) at 0 °C under Ar. The resulting solution was stirred at 0 °C for 2 h. Then, saturated $NaHCO_{3(aq)}$ (10 mL) was added and the mixture was extracted with EtOAc (3 × 30 mL). The combined organic layers were dried ($MgSO_4$) and evaporated under reduced pressure to give the crude product. Purification by flash column chromatography on silica with 3:1 hexane-EtOAc as eluent gave carbamate **6** (13 mg, 16%) as a white solid, mp 122-125 °C, R_F (10:1 CH_2Cl_2-MeOH) 0.3; IR (ATR) 1672 (C=O), 1620, 1602, 1577 cm^{-1}; ^1H NMR (400 MHz, $CDCl_3$) δ 8.51 (d, J = 9.0 Hz, 1H, Ar), 8.25 (br s, 1H, NH), 8.20 (d, J = 9.0, 1H, Ar), 7.82 (d, J = 8.0 Hz, 1H, Ar), 7.80 (d, J = 8.0 Hz, 1H, Ar), 7.68 (ddd, J = 8.0, 7.0, 1.0 Hz, 1H, Ar), 7.47 (ddd, J = 8.0, 7.0, 1.0 Hz, 1H, Ar), 6.52 (dd, J = 17.0, 1.0 Hz, 1H, =CH), 6.31 (dd, J = 17.0, 10.0 Hz, 1H, =CH), 5.86 (dd, J = 10.0, 1.0 Hz, 1H, =CH); ^{13}C NMR (100.6 MHz, $CDCl_3$) δ 164.2 (C=O), 151.2 (*ipso*-Ar), 146.6 (*ipso*-Ar), 138.9 (Ar), 131,1 (Ar), 130.2 (Ar), 129.1 (=CH_2), 127.7 (Ar), 127.3 (Ar), 126.5 (*ipso*-Ar), 125.4 (Ar), 114.7 (=CH); MS (ESI) *m/z* 199 [$(M + H)^+$, 100]; HRMS (ESI) *m/z* calcd for $C_{12}H_{10}N_2O$ $(M + H)^+$ 199.0866, found 199.0861 (+2.6 ppm error).

Lab Book Reference: MA 4-38

N-(Isoquinolin-3-yl)acrylamide 7

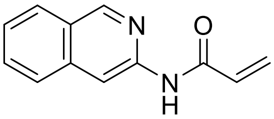

7

Acryloyl chloride (34 μL, 0.42 mmol, 1.0 eq.) was added dropwise to a stirred solution of 3-aminoisoquinoline (60 mg, 0.42 mmol, 1.0 eq.) and KHMDS (833 μL of a 0.5 M solution in toluene, 0.42 mmol, 4.0 eq.) in THF (4.2 mL) at 0 °C under Ar. The resulting solution was stirred at 0 °C for 2 h. Then, saturated $NaHCO_{3(aq)}$ (10 mL) was added and the mixture was extracted with EtOAc (3 × 30 mL). The combined organic layers were dried ($MgSO_4$) and evaporated under reduced pressure to give the crude product. Purification by flash column chromatography on silica with 3:1 hexane-EtOAc as eluent gave carbamate **7** (12 mg, 15%) as a white solid, mp 125-128°C, R_F (10:1 CH_2Cl_2-MeOH) 0.3; IR (ATR) 3252, 1695 (C=O), 1628, 1530 cm^{-1}; ^1H NMR (400 MHz, $CDCl_3$) δ 8.99 (s, 1H, Ar), 8.67 (s, 1H, Ar), 8.11 (br s, 1H, NH), 7.91 (br d, J = 8.0 Hz, 1H, Ar), 7.84 (br d, J = 8.0 Hz, 1H, Ar), 7.66 (ddd, J = 8.0, 7.0, 1.0 Hz, 1H, Ar), 7.49 (ddd, J = 8.0, 7.0, 1.0 Hz, 1H, Ar), 6.52 (dd, J = 17.0, 1.0 Hz, 1H, =CH), 6.32 (dd, J = 17.0, 10.0 Hz, 1H, =CH), 5.84 (dd, J = 10.0, 1.0 Hz, 1H, =CH); ^{13}C NMR (100.6 MHz, $CDCl_3$) δ 163.7 (C=O), 151.1 (Ar), 146.2 (*ipso*-Ar), 138.0 (*ipso*-Ar), 131,1 (Ar), 131.0 (Ar), 128.4 (=CH_2), 127.5 (Ar), 127.0 (Ar), 126.6 (*ipso*-Ar), 126.1 (Ar), 108.6 (=CH); MS (ESI) *m/z* 199 [$(M + H)^+$, 100]; HRMS (ESI) *m/z* calcd for $C_{12}H_{10}N_2O$ $(M + H)^+$ 199.0866, found 199.0859 (+3.9 ppm error).

Lab Book Reference: MA 4-39

***tert*-Butyl (4-Morpholinothieno[2,3-d]pyrimidin-2-yl)carbamate S8**

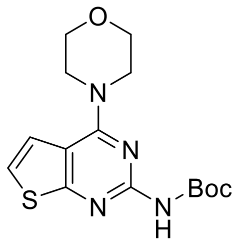

S8

$Pd(OAc)_2$ (11 mg, 0.05 mmol, 0.1 eq.) was added dropwise to a stirred solution of 4-(2-chlorothieno[2,3-d]pyrimidin-4-yl)morpholine (158 mg, 0.62 mmol, 1.0 eq.), $BocNH_2$ (176 mg, 1.5 mmol, 3.0 eq.), BippyPhos (76 mg, 0.15 mmol, 0.3 eq.) and Cs_2CO_3 (228 mg, 0.7 mmol, 1.4 eq.) in dioxane (4 mL) at rt. The resulting solution was stirred and heated at 110 °C for 3 h. The precipitate was removed by filtration over Celite® and the filtrate was evaporated under reduced pressure to give the crude product. Purification by flash column chromatography on silica with 100:9:1CH_2Cl_2-MeOH-$NH_4OH_{(aq)}$ as eluent gave carbamate **S8** (36 mg, 86%) as a yellow amorphous solid, mp 178-180 °C, R_F (100:9:1 CH_2Cl_2-MeOH-$NH_4OH_{(aq)}$) 0.3; IR (ATR) 2972, 2856, 1741 (C=O), 1561, 1512 cm^{-1}; ^1H NMR (400 MHz, $CDCl_3$) δ 7.23 (d, J = 6.5 Hz, 1H, Ar), 7.08 (d, J = 6.5 Hz, 1H, Ar), 3.91 (t, J = 6.0 Hz, 4H, CH_2), 3.81 (t, J = 6.0 Hz, 4H, CH_2), 1.52 (s, 9H, CMe_3); ^{13}C NMR (100.6 MHz, $CDCl_3$) (mixture of rotamers) δ 171.3 (C=O), 159.0 (*ipso*-Ar), 153.2 (*ipso*-Ar), 150.7 (*ipso*-Ar), 120.0 (Ar), 119.8 (Ar), 112.6 (*ipso*-Ar), 81.0 (*C*Me_3), 67.2 (OCH_2), 66.8 (OCH_2), 47.1 (NCH_2), 28.4 (C*Me*$_3$); MS (ESI) *m/z* 337 [$(M + H)^+$, 100]; HRMS (ESI) *m/z* calcd for $C_{15}H_{20}N_4O_3S$ $(M + H)^+$ 337.1329, found 337.1328 (−0.0 ppm error).

Lab Book Reference: MA 4-62

4-Morpholinothieno[2,3-d]pyrimidin-2-amine S9

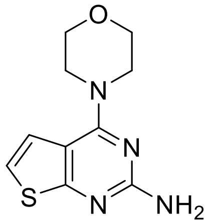

S9

TFA (2 mL) was added dropwise to a stirred solution of carbamate **S8** (134 mg, 0.4 mmol, 1.0 eq.) in CH_2Cl_2 (4 mL) at 0 °C. The resulting solution was stirred at rt for 1 h and the solvent was removed under reduced pressure. $NH_4OH_{(aq)}$ (5 mL) was added and the mixture was extracted with CH_2Cl_2 (3 × 10 mL). The combined organic layers were dried ($MgSO_4$) and evaporated under reduced pressure to give the crude product. Purification by flash column chromatography on silica with 100:9:1 CH_2Cl_2-MeOH-$NH_4OH_{(aq)}$ as eluent gave amine **S9** (68 mg, 72%) as a white solid, mp 132-134°C, R_F (10:1 CH_2Cl_2-MeOH) 0.3; IR (ATR) 3171 (NH_2), 1631, 1532 cm^{-1}; ^1H NMR (400 MHz, $CDCl_3$) δ 7.15 (d, 1H, J = 6.5 Hz, Ar), 6.87 (d, 1H, J = 6.5 Hz, Ar), 4.78 (br s, 2H, NH_2), 3.86-3.79 (m, 8H, CH_2); ^{13}C NMR (100.6 MHz, $CDCl_3$) δ 172.1 (*ipso*-Ar), 159.8 (*ipso*-Ar), 159.4 (*ipso*-Ar), 120.4 (Ar), 116.6 (Ar), 110.4 (*ipso*-Ar), 66.8 (OCH_2), 47.2 (NCH_2); MS (ESI) m/z 237 [(M + H)$^+$, 100]; HRMS (ESI) m/z calcd for $C_{10}H_{12}N_3OS$ (M + H)$^+$ 237.0805, found 237.0800 (+2.4 ppm error).

Lab Book Reference: MA 4-63

N-(4-Morpholinothieno[2,3-d]pyrimidin-2-yl)acrylamide 8

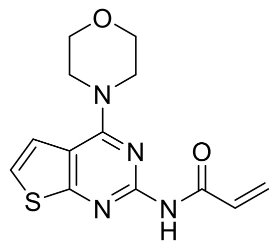

8

Acryloyl chloride (24 μL, 0.29 mmol, 1.0 eq.) was added dropwise to a stirred solution of amine **S9** (69 mg, 0.29 mmol, 1.0 eq.) and Et_3N (41 μL, 0.29 mmol, 1.0 eq.) in CH_2Cl_2-DMF (3 mL) at 0 °C. The resulting solution was stirred at 0 °C for 3 h. Then, saturated $NaHCO_{3(aq)}$ (5 mL) was added and the mixture was extracted with EtOAc (3 × 10 mL). The combined organic layers were dried ($MgSO_4$) and evaporated under reduced pressure to give the crude product. Purification by flash column chromatography on silica with 50:1 CH_2Cl_2-MeOH as eluent gave 35 mg of a 1:1 mixture of amine **S9** (23%) and carbamate **8** (23%) as a white solid, mp 178-180 °C, R_F (10:1 CH_2Cl_2-MeOH) 0.3; IR (ATR) 1684 (C=O), 1585 cm^{-1}; ^1H NMR of **8** (400 MHz, $CDCl_3$) δ 7.97 (br s, 1H, NH), 7.28 (d, J = 6.5 Hz, 1H, Ar), 7.00-6.82 (m, 1H, =CH), 7.14 (d, J = 6.5 Hz, 1H, Ar), 6.49 (dd, J = 17.0, 1.0 Hz, 1H, =CH), 5.81 (dd, J = 10.0, 1.0 Hz, 1H, =CH), 3.96-3.93 (m, 4H, CH_2), 3.85-3.80 (m, 4H, CH_2); ^{13}C NMR of **8** (100.6 MHz, $CDCl_3$) δ 170.8 (C=O), 159.0 (*ipso*-Ar), 152.8 (*ipso*-Ar), 130.9 (Ar), 128.9 (=CH_2), 120.4 (Ar), 120.3 (*ipso*-Ar), 120.3 (=CH), 112.9 (*ipso*-Ar), 66.8 (OCH_2), 47.2 (NCH_2); MS (ESI) *m/z* 291 [$(M + H)^+$, 100]; HRMS (ESI) *m/z* calcd for $C_{13}H_{14}N_4O_2S$ $(M + H)^+$ 291.0910, found 291.0909 (+0.4 ppm error).

Lab Book Reference: MA 4-71

(3-Aminopyridin-4-yl)methanol S10

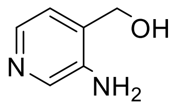

S10

A suspension of 3-aminopyridine-4-carboxylic acid (2.07 g, 15.0 mmol, 1.0 eq.) in THF (20 mL) was added to a suspension of $LiAlH_4$ (1.71 g, 45.0 mmol, 3.0 eq.) in THF (20 mL) at 0 °C under N_2. The resulting mixture was warmed to rt and stirred for 16 h. After cooling to 0 °C, the mixture was diluted with Et_2O (50 mL). Then water (1.7 mL), 2M $NaOH_{(aq)}$ (3.4 mL) and water (1.7 mL) were sequentially added dropwise and the mixture was warmed to rt and stirred for 30 min. Then, anhydrous $MgSO_4$ was added and the mixture was stirred for 1 h. The solids were removed by filtration through Celite® and washed with EtOAc (50 mL). The filtrate was evaporated under reduced pressure to give alcohol **S10** (1.22 g, 66%) as a yellow solid that was used without further purification, mp 106-108 °C (lit.,[4] 113-115 °C); IR (ATR) 3342, 3073, 2908, 2846, 2733, 1961, 1886, 1627, 1427, 1011, 781, 762, 742, 627, 545, 460 cm^{-1}; ^1H NMR (400 MHz, MeOD-*d₄*) δ 7.94 (s, 1H, Ar), 7.81 (d, J = 5.0 Hz, 1H, Ar), 7.20 (d, J = 5.0 Hz, 1H, Ar), 4.57 (s, 2H, CH_2); ^{13}C NMR (100.6 MHz, MeOD-d_4) δ 143.9 (*ipso*-Ar), 138.8 (Ar), 137.2 (Ar), 135.3 (*ipso*-Ar), 123.0 (Ar), 61.5 (CH_2); HRMS (ESI) *m/z* calcd for $C_6H_8N_2O$ $(M + H)^+$ 125.0709, found 125.0707 (+2.2 ppm error). Spectroscopic data consistent with those reported in the literature.[4]

Lab Book Reference: JDF_A005

***tert*-Butyl *N*-(1,7-naphthyridin-2-yl)carbamate S13**

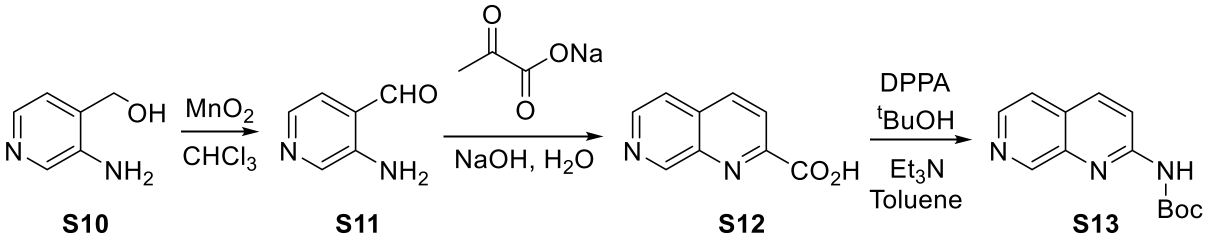

Activated MnO_2 (8.37 g, 96.0 mmol, 10.0 eq.) was added to a stirred suspension of alcohol **S10** (1.20 g, 9.6 mmol, 1.0 eq.) in $CHCl_3$ (100 mL) at rt and the resulting suspension was stirred at rt for 48 h. The solids were removed by filtration through Celite® and washed with $CHCl_3$ (100 mL). The filtrate was evaporated under reduced pressure to give crude aldehyde **S11** (752 mg, 64%) as a yellow solid which was used without further purification, ^{1}H NMR (400 MHz, $CDCl_3$) δ 10.04 (s, 1H, CHO), 8.87 (s, 1H, Ar), 7.95 (d, J = 5.5 Hz, 1H, Ar), 7.62 (d, J = 5.5 Hz, 1H, Ar). Spectroscopic data consistent with those reported in the literature.[4]

NaOH (342 mg, 8.6 mmol, 1.5 eq.) was added to a stirred solution of crude aldehyde **S11** (700 mg, 5.7 mmol, 1.0 eq.) and sodium pyruvate (693 mg, 6.3 mmol, 1.1 eq.) in water (25 mL) a rt. The resulting solution was stirred at rt for 3 h. The solution was acidified to pH 1-2 with 1 M $HCl_{(aq)}$ and the resulting solution was evaporated under reduced pressure. MeOH (20 mL) was added and the solids were removed by filtration through Celite® and washed with MeOH (50 mL). The filtrate was evaporated under reduced pressure to give crude acid **S12** (1.64 g) as a white solid that was used without further purification, ^{1}H NMR (400 MHz, MeOD-d_4) δ 9.92 (d, J = 0.5 Hz, 1H, Ar), 8.86 (dd, J = 8.5, 0.5 Hz, 1H, Ar), 8.81 (dd, J = 6.5, 0.5 Hz, 1H, Ar), 8.68 (d, J = 8.5 Hz, 1H, Ar), 8.59 (dd, J = 6.5, 0.5 Hz, 1H, Ar).

Diphenylphosphoryl azide (1.47 µL, 6.84 mmol, 1.2 eq.) was added dropwise to a stirred solution of crude carboxylic acid **S12** (1.6 g, 5.70 mmol max., 1.0eq.), Et_3N (2.38 mL, 17.1 mmol, 3.0 eq.) and ^{t}BuOH (1.64 mL, 17.1 mmol, 3.0 eq.) in toluene (25 mL) at rt. The resulting solution was stirred and heated at 70 °C for 16 h. After cooling to rt, EtOAc (50 mL) and water (30 mL) were added and the layers separated. The aqueous layer was extracted with EtOAc (3 × 30 mL) and the combined organic layers were dried ($MgSO_4$), filtered and evaporated under reduced pressure. Purification by flash column chromatography on silica with 1:1 hexane-EtOAc as eluent gave carbamate **S13** (280 mg, 22%) as a white solid, mp 248-252 °C (decomp.); R_F (1:1 hexane-EtOAc) 0.17; IR (ATR) 2976, 2934, 1718 (C=O), 1618, 1602, 1323, 1234, 1154, 1072, 846, 774, 640 cm^{-1}; ^{1}H NMR (400 MHz, $CDCl_3$) δ 9.25 (s, 1H, Ar), 8.55–8.45 (m, 2H, Ar), 8.36 (br s, 1H, NH), 8.15 (d, J = 9.0 Hz, 1H, Ar), 7.63 (d, J = 5.5 Hz, 1H, Ar), 1.51 (s, 9H, C*Me*$_3$); ^{13}C NMR (100.6 MHz, $CDCl_3$) δ 153.1 (C=O), 152.3 (*ipso*-Ar), 151.0 (Ar), 141.8 (*ipso*-Ar), 141.1 (Ar), 136.9 (Ar), 129.1 (*ipso*-Ar), 120.0 (Ar), 118.0 (Ar),

81.9 (*C*Me$_3$), 28.1 (C*Me*$_3$); HRMS (ESI) *m/z* calcd for $C_{13}H_{15}N_3O_2$ (M + H)$^+$ 246.1237, found 246.1246 (–3.2 ppm error).

Lab Book Reference: JDF_A010

N-(1,7-Naphthyridin-2-yl)prop-2-enamide 9

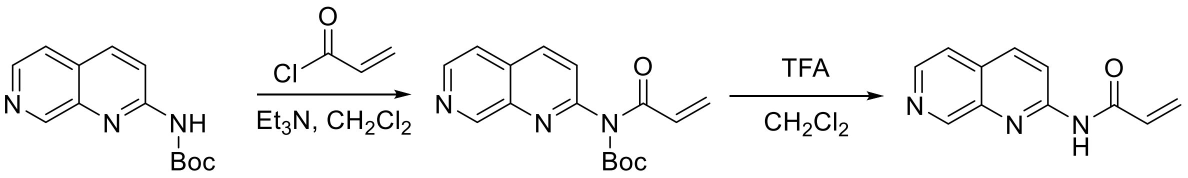

S13 **S14** **9**

Acryloyl chloride (106 µL, 1.30 mmol, 2.0 eq.) was added dropwise to a stirred solution of amine **S13** (160 mg, 0.65 mmol, 1.0 eq.) and Et_3N (272 µL, 1.95 mmol, 3.0 eq.) in CH_2Cl_2 (5 mL) at 0 °C. The resulting solution was warmed to rt and stirred at rt for 2 h. Then, saturated $NaHCO_{3(aq)}$ (5 mL) was added and the mixture was extracted with CH_2Cl_2 (3 × 10 mL). The combined organic layers were dried ($MgSO_4$) and evaporated under reduced pressure. Filtration through a plug of silica with 1:1 hexane-EtOAc as eluent gave the crude acrylamide **S14** (54 mg) which was used in the next step without further purification. TFA (0.5 mL) was added to a stirred solution of crude acrylamide **S14** (54 mg) in CH_2Cl_2 (2 mL) and the resulting solution was stirred at rt for 2 h. Then, saturated $NaHCO_{3(aq)}$ (5 mL) was added and the mixture was extracted with CH_2Cl_2 (3 × 10 mL). The combined organic layers were dried ($MgSO_4$) and evaporated under reduced pressure to give the crude product. Purification by flash column chromatography on silica with 96:4 CH_2Cl_2-MeOH as eluent gave acrylamide **9** (10 mg, 8%) as a white solid, mp 168-169 °C, R_F (96:4 CH_2Cl_2-MeOH) 0.16; IR (ATR) 2916, 1693 (C=O), 1628, 1600, 1582, 1322, 1193, 857, 789, 638 cm^{-1}; ^{1}H NMR (400 MHz, $CDCl_3$) δ 9.27 (s, 1H, Ar), 8.74 (d, *J* = 9.0 Hz, 1H, Ar), 8.62–8.45 (m, 2H, Ar + NH), 8.19 (d, *J* = 9.0 Hz, 1H, Ar), 7.63 (d, *J* = 5.5 Hz, 1H, Ar), 6.55 (dd, *J* = 17.0, 1.0 Hz, 1H, =CH), 6.36 (dd, *J* = 17.0, 10.0 Hz, 1H, =CH), 5.90 (dd, *J* = 10.0, 1.0 Hz, 1H, =CH); ^{13}C NMR (100.6 MHz, $CDCl_3$) δ 164.2 (C=O), 152.3 (*ipso*-Ar), 152.1 (Ar), 142.9 (Ar), 141.9 (*ipso*-Ar), 137.4 (Ar), 130.8 (=CH), 129.7 (=CH_2), 129.4 (*ipso*-Ar), 119.8 (Ar), 119.0 (Ar); HRMS (ESI) *m/z* calcd for $C_{11}H_9N_3O$ (M + H)$^+$ 200.0818, found 200.0815 (1.6 ppm error).

Lab Book Reference: JDF_A015

***tert*-Butyl *N*-(1,6-naphthyridin-2-yl)carbamate S15**

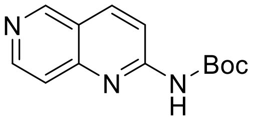

S15

Diphenylphosphoryl azide (647 µL, 3.0 mmol, 1.0 eq.) was added dropwise to a stirred solution of 1,6-naphthyridine-2-carboxylic acid (522 mg, 3.0 mmol, 1.0 eq.), Et_3N (627 µL, 4.5 mmol, 1.5 eq.) and ^{t}BuOH (717 µL, 7.5 mmol, 2.5 eq.) in toluene (10 mL) at rt. The resulting solution was stirred and heated at 80 °C for 12 h. After cooling to rt EtOAc (20 mL) and water (30 mL) were added and the layers separated. The aqueous layer was extracted with EtOAc (3 × 20 mL) and the combined organic were layers dried ($MgSO_4$) and evaporated under reduced pressure. Purification by flash column chromatography on silica with 1:1 hexane-EtOAc as eluent gave carbamate **S15** (350 mg, 48%) as a white solid, mp 143-145 °C, R_F (1:1 hexane-EtOAc) 0.16; IR (ATR) 2973, 1962, 1727 (C=O), 1604, 1475, 1391, 1338, 1162, 1108, 831 cm^{-1}; ^{1}H NMR (400 MHz, MeOD-*d₄*) δ 9.11 (s, 1H, Ar), 8.53 (d, J = 6.0 Hz, 1H, Ar), 8.40 (d, J = 9.0 Hz, 1H, Ar), 8.26 (d, J = 9.0 Hz, 1H, Ar), 7.70 (d, J = 6.0 Hz, 1H, Ar), 1.57 (s, 9H, C*Me*$_3$); ^{13}C NMR (100.6 MHz, MeOD-d_4) δ 157.6 (*ipso*-Ar), 154.2 (C=O), 152.7 (Ar), 151.6 (*ipso*-Ar), 147.2 (Ar), 139.3 (Ar), 122.9 (*ipso*-Ar), 122.1 (Ar), 116.0 (Ar), 82.4 (*C*Me$_3$), 28.5 (CMe$_3$); HRMS (ESI) *m/z* calcd for $C_{13}H_{15}N_3O_2$ (M + H)$^+$ 246.1237, found 246.1229 (3.0 ppm error).

Lab Book Reference: JDF_A012

_N_-(1,6-Naphthyridin-2-yl)prop-2-enamide 10

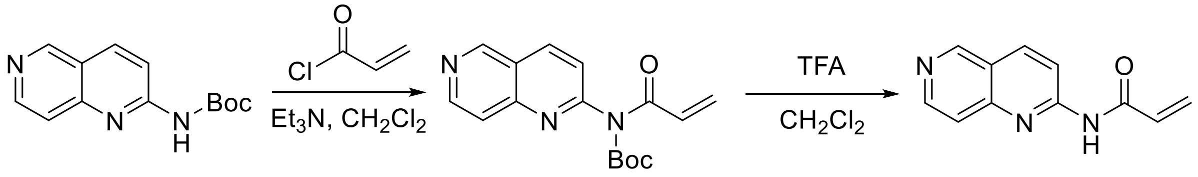

S15 **S16** **10**

Acryloyl chloride (106 µL, 1.30 mmol, 2.0 eq.) was added dropwise to a stirred solution of amine **S15** (160 mg, 0.65 mmol, 1.0 eq.) and Et_3N (272 µL, 1.95 mmol, 3.0 eq.) in CH_2Cl_2 (5 mL) at 0 °C. The resulting solution was warmed to rt and stirred at rt for 2 h. Then, saturated $NaHCO_{3(aq)}$ (5 mL) was added and the mixture was extracted with CH_2Cl_2 (3 × 10 mL). The combined organic layers were dried ($MgSO_4$) and evaporated under reduced pressure. Filtration through a plug of silica with 1:1 hexane-EtOAc as eluent gave the crude acrylamide **S16** (154 mg) which was used in the next step without further purification. TFA (0.5 mL) was added to a stirred solution of crude acrylamide **S16** (154 mg) in CH_2Cl_2 (2 mL) and the resulting solution was stirred at rt for 2 h. Then, saturated $NaHCO_{3(aq)}$ (5 mL) was added and the mixture was extracted with CH_2Cl_2 (3 × 10 mL). The combined organic layers were dried ($MgSO_4$) and evaporated under reduced pressure to give the crude product. Purification by flash column chromatography on silica with 1:1 to 3:7 hexane-EtOAc as eluent gave acrylamide **10** (13 mg, 7%) as a white solid, mp 165-166 °C, R_F (3:7 hexane-EtOAc) 0.15; IR (ATR) 3113, 2922, 2852, 2303, 1923, 1739, 1688 (C=O), 1615, 1466, 1408, 1330, 1263, 961, 943, 840, 793 cm^{-1}; ^{1}H NMR (400 MHz, MeOD-d_4) δ 9.14 (s, 1H, Ar), 8.54-5.83 (m, 2H, Ar), 8.47–8.37 (m, 1H, Ar), 7.72 (d, J = 6.0 Hz, 1H, Ar), 6.63–6.38 (m, 2H, =CH), 5.86 (dd, J = 9.5, 2.0 Hz, 1H, =CH); ^{13}C NMR (100.6 MHz, MeOD-d_4) δ 166.7 (C=O), 157.0 (*ipso*-Ar), 153.0 (Ar), 151.5 (*ipso*-Ar), 147.2 (Ar), 139.5 (Ar), 132.0 (Ar), 129.8 (=CH_2), 123.4 (*ipso*-Ar), 122.4 (=CH), 117.5 (Ar); HRMS (ESI) *m/z* calcd for $C_{11}H_9N_3O$ $(M + H)^+$ 200.0818, found 200.0823 (–1.8 ppm error).

Lab Book Reference: JDF_A017

References for synthesis section

(1) Süsse, M., Adler, F. & Johne, S. Chinazolincarbonsäuren. XII. Mitteilung. Synthese von [2-(Ethoxycarbonyl)-3,4-dihydro-4-oxochinazolin-3-yl]-, [2-(Ethoxycarbonyl)chinazolin-4-yloxy]- und (5,6,7,8-Tetrahydro-2-phenylchinazolin-4-ylthio)alkansäure-estern. *Helv. Chim. Acta.* **69**, 1017-1024 (1986).

(2) Yuan, Y., Zaidi, S. A., Stevens, D. L., Scoggins, K. I, Mosier, P. D., Kellogg, G. E., Dewey, W. L., Selley, D. E. & Zhang, Y. Design, syntheses, and pharmacological characterization of 17-cyclopropylmethyl-3,14β-dihydroxy-4,5α-epoxy-6α-(isoquinoline-3′-carboxamido)morphinan analogues as opioid receptor ligands. *Bioorg. Med. Chem.*, ***23***, 1701–1715 (2015).

(3) Greco, C. V. & Warchol, J. F. Cyclization of some 2-(haloacylamino)pyrimidines. *J. Org. Chem.*, ***36***, 604–607 (1971).

(4) Chen, Q. P. & Deady, L. W. Synthesis of Some Benzo [*b*][1,6]naphthyridines and Benzo [*b*][1,7]naphthyridine. *Aust. J. Chem.*, ***46***, 987–993 (1993).

NMR Spectra: 400 MHz ^{1}H NMR spectrum; 100.6 MHz ^{13}C NMR spectrum; DMSO-d_6

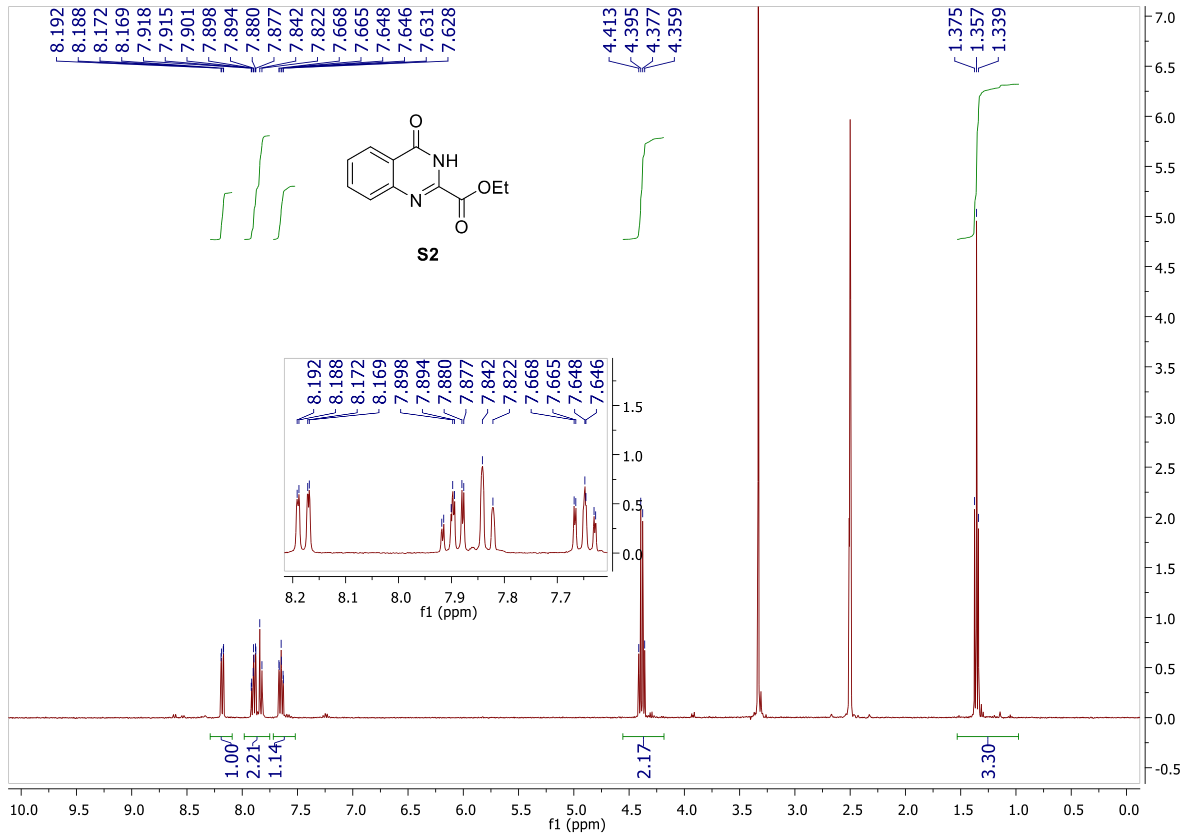

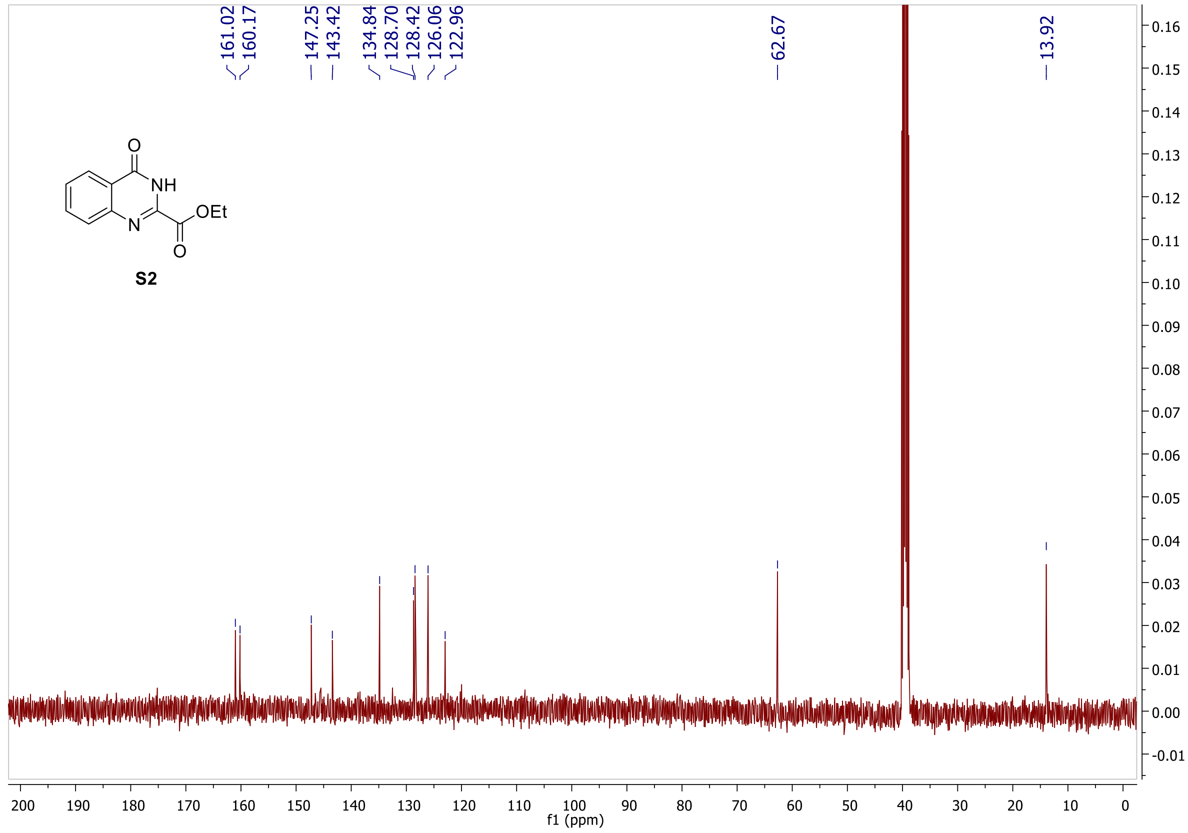

400 MHz ^{1}H NMR spectrum; 100.6 MHz ^{13}C NMR spectrum; $CDCl_3$

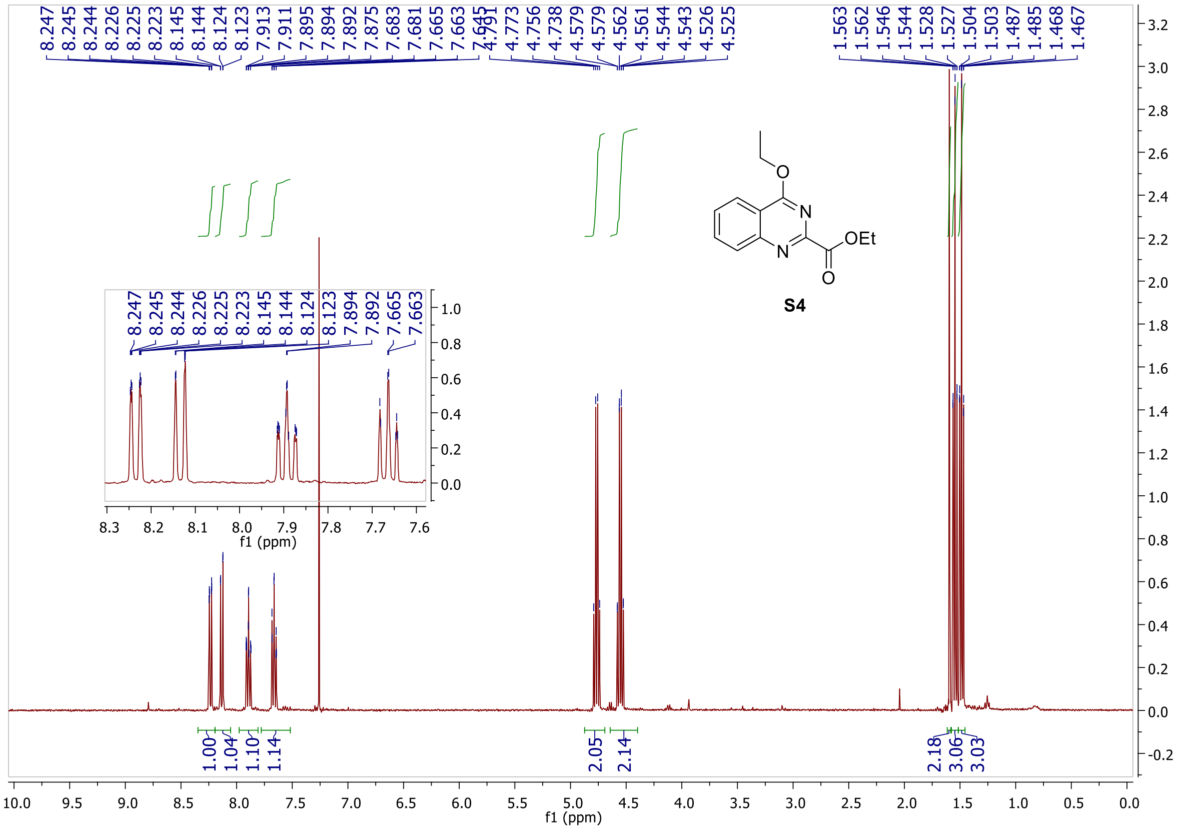

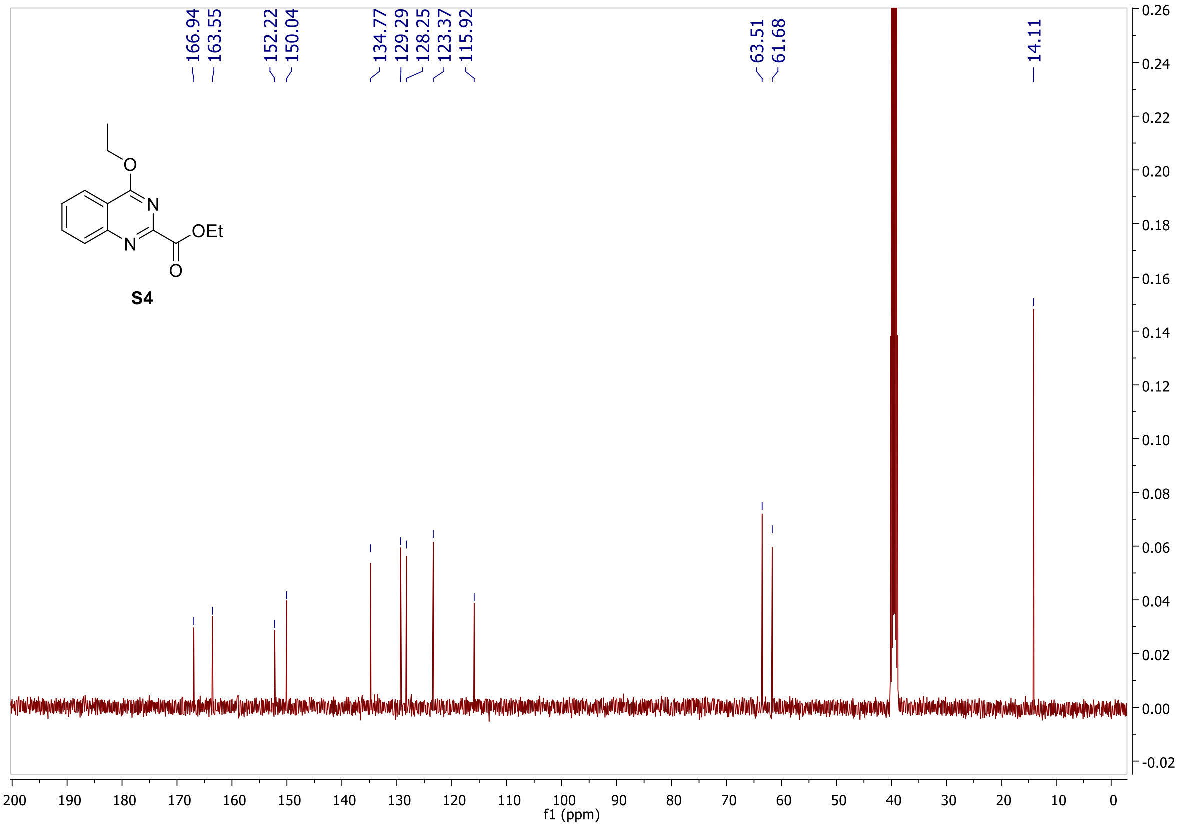

400 MHz ^{1}H NMR spectrum; 100.6 MHz ^{13}C NMR spectrum; DMSO-d_6

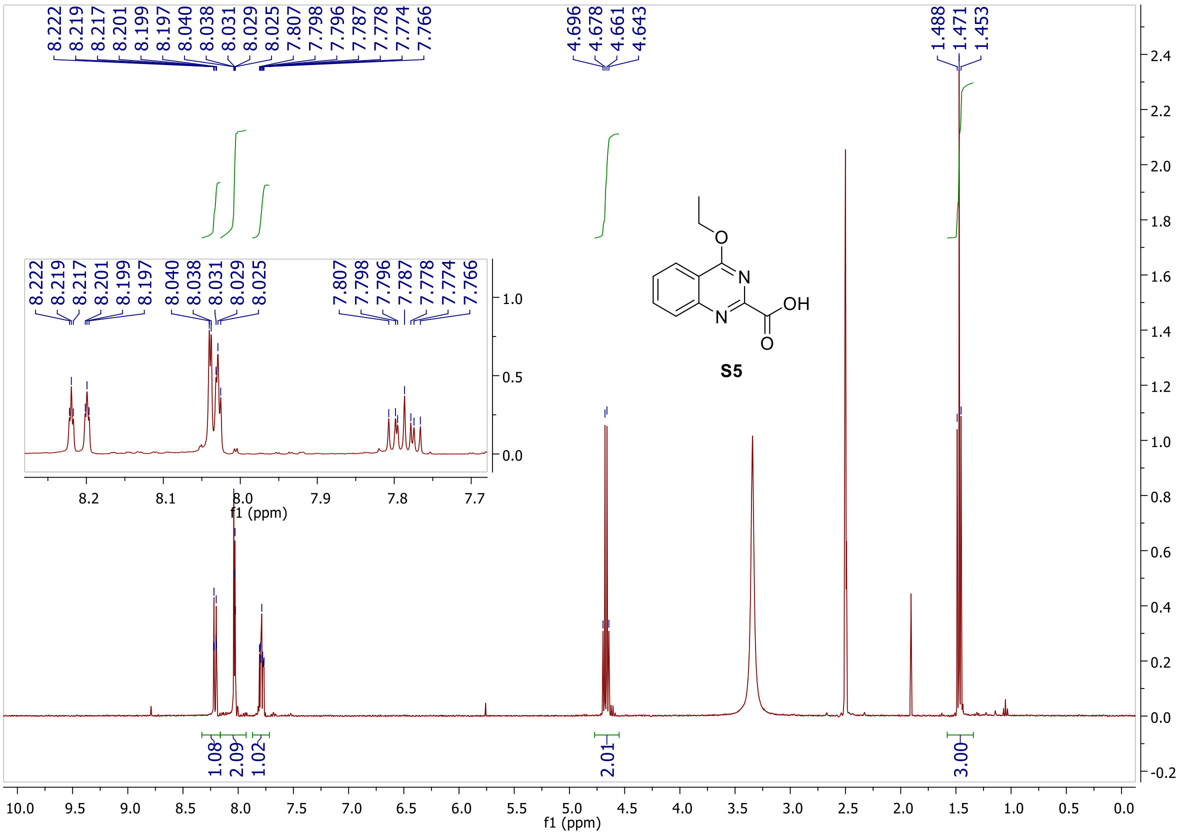

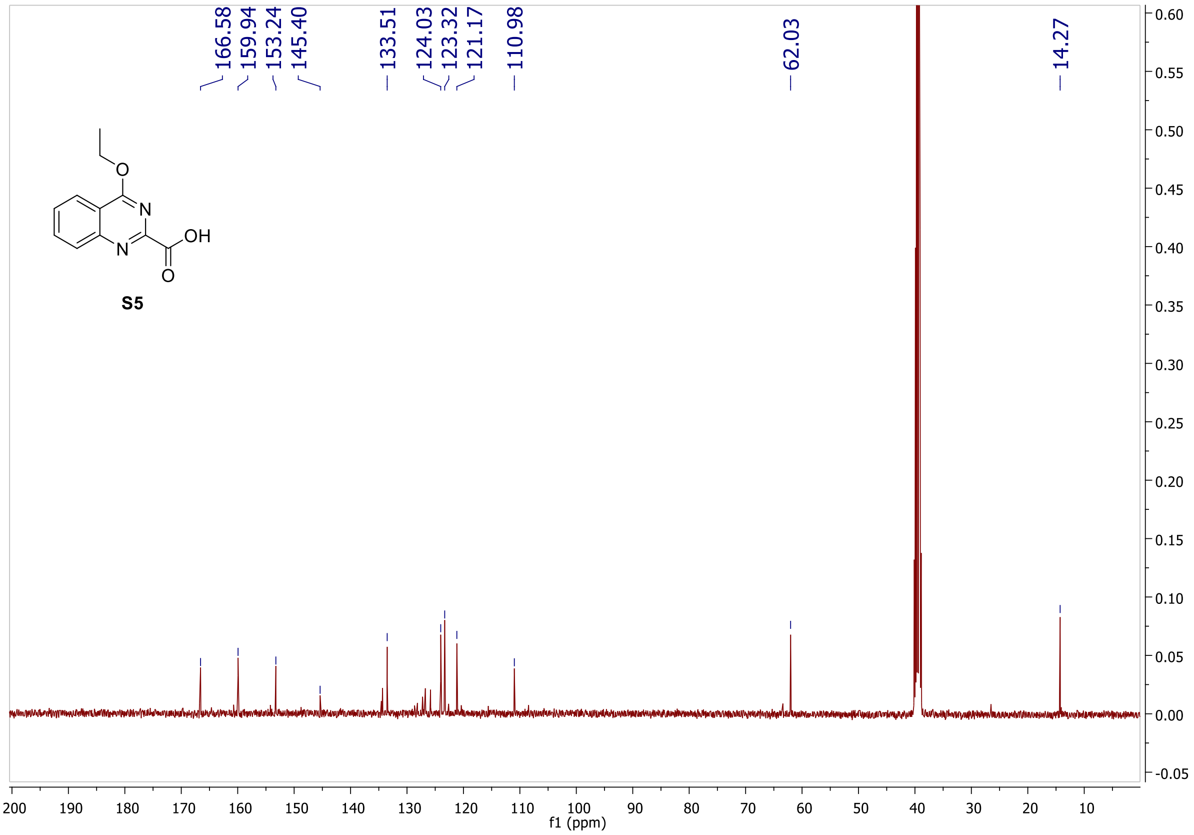

400 MHz ^{1}H NMR spectrum; 100.6 MHz ^{13}C NMR spectrum; $CDCl_3$

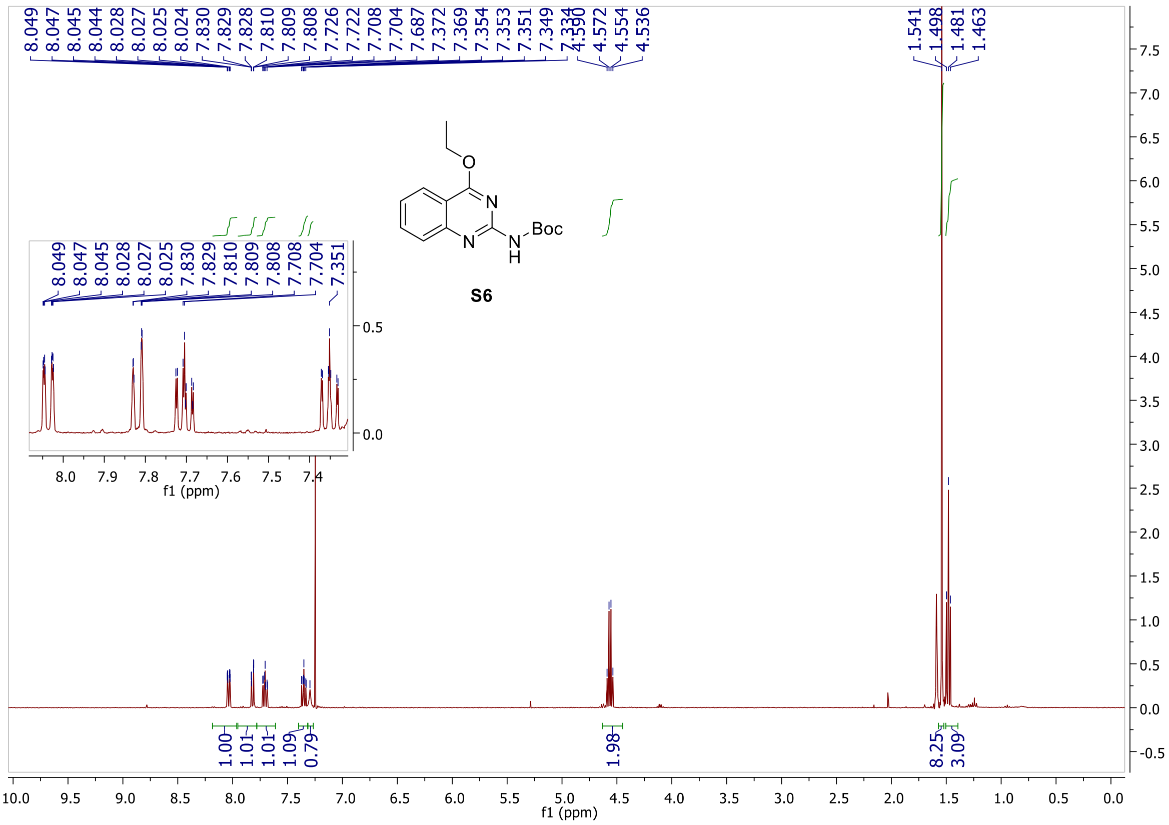

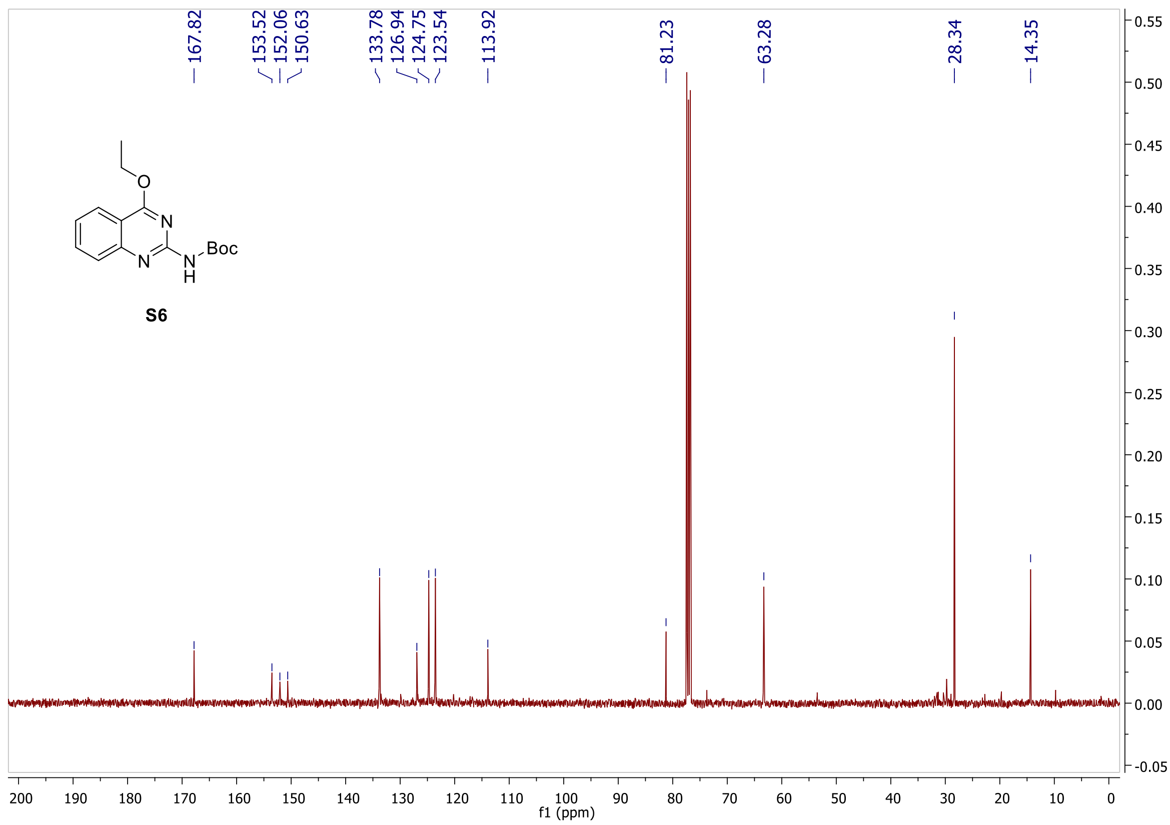

400 MHz ^{1}H NMR spectrum; 100.6 MHz ^{13}C NMR spectrum; DMSO-d_6

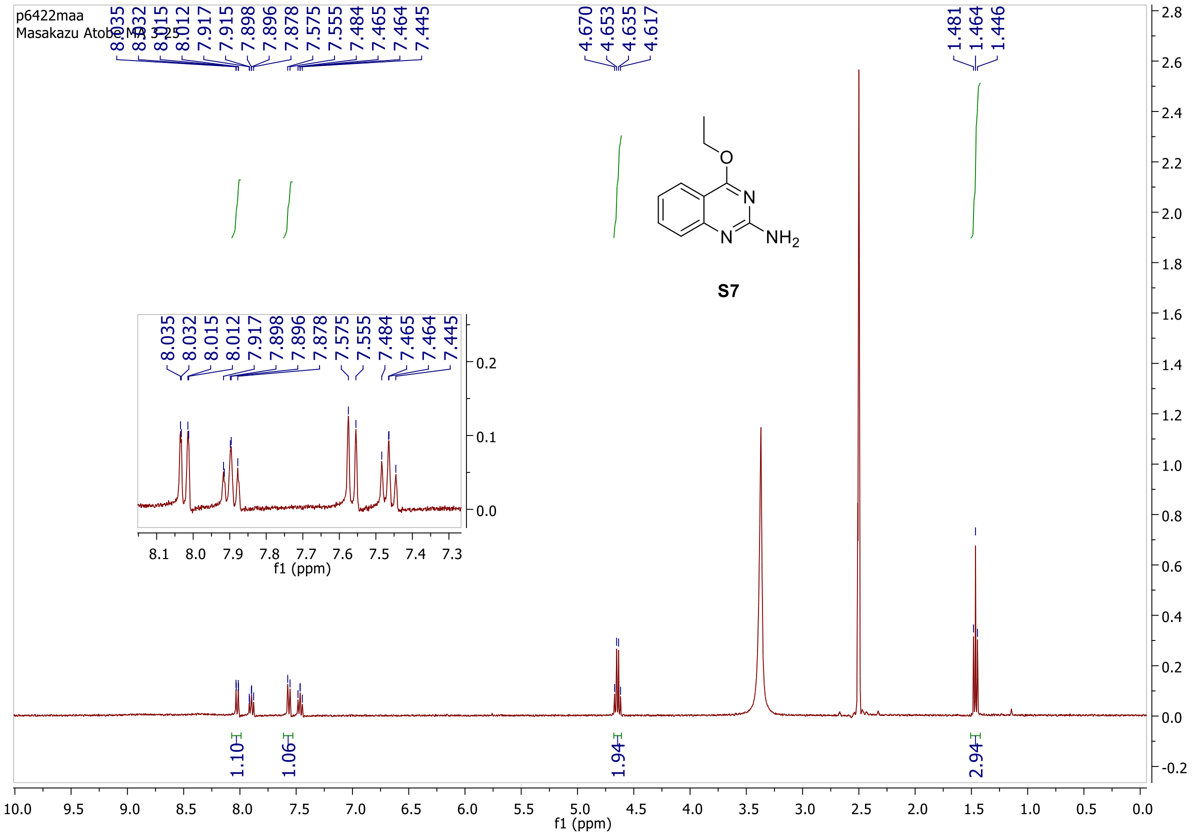

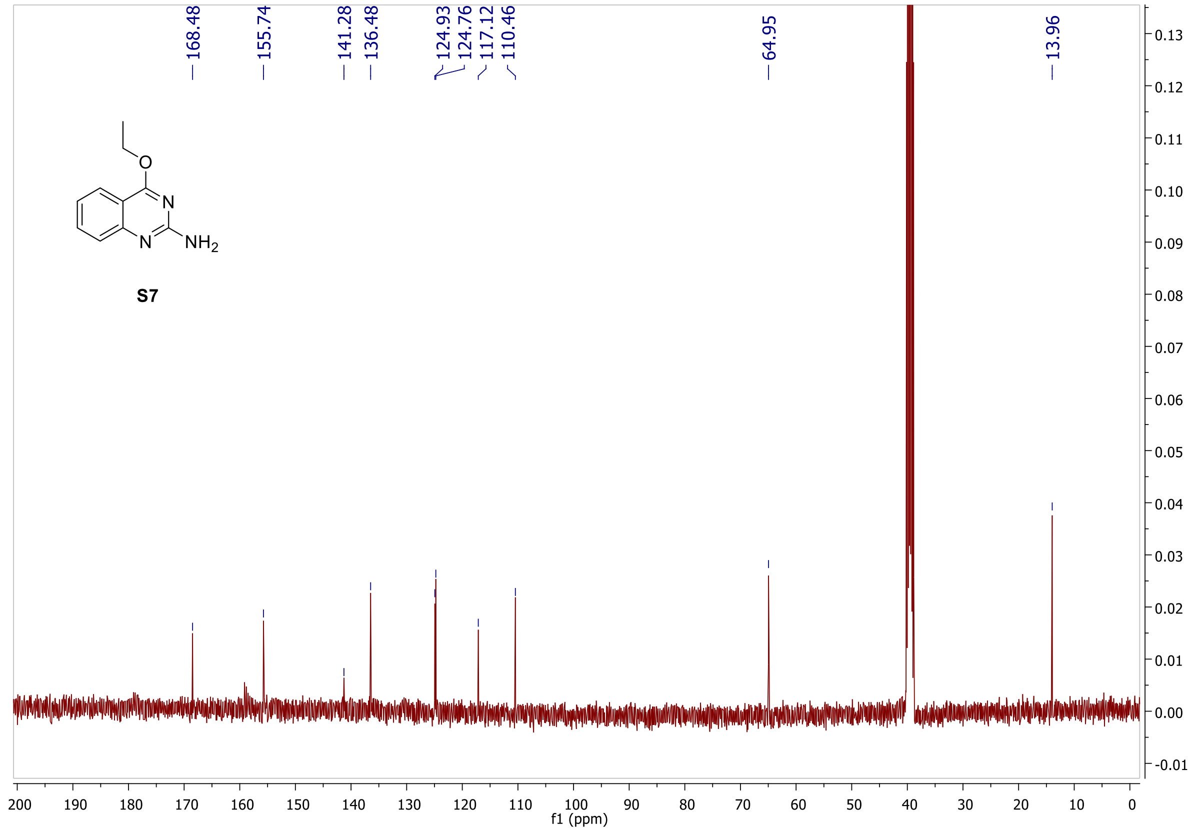

400 MHz ^{1}H NMR spectrum; 100.6 MHz ^{13}C NMR spectrum; $CDCl_3$

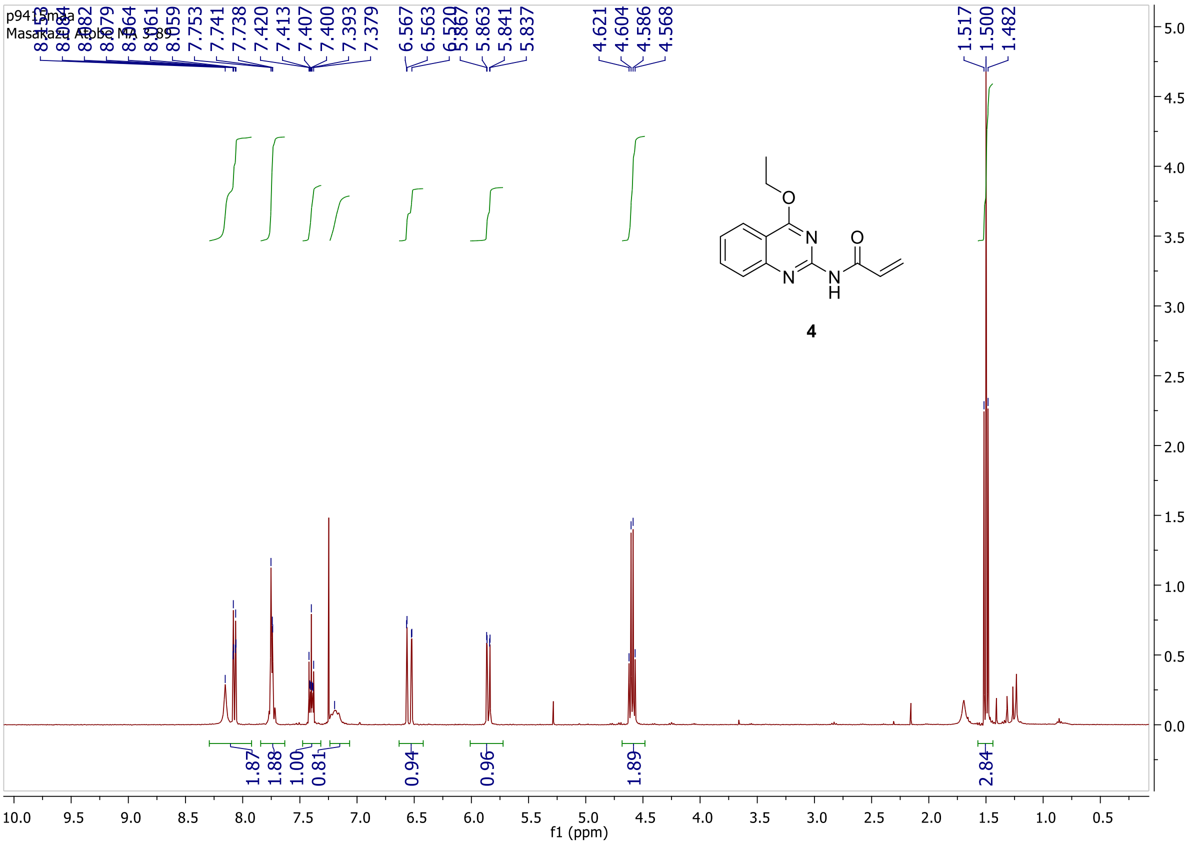

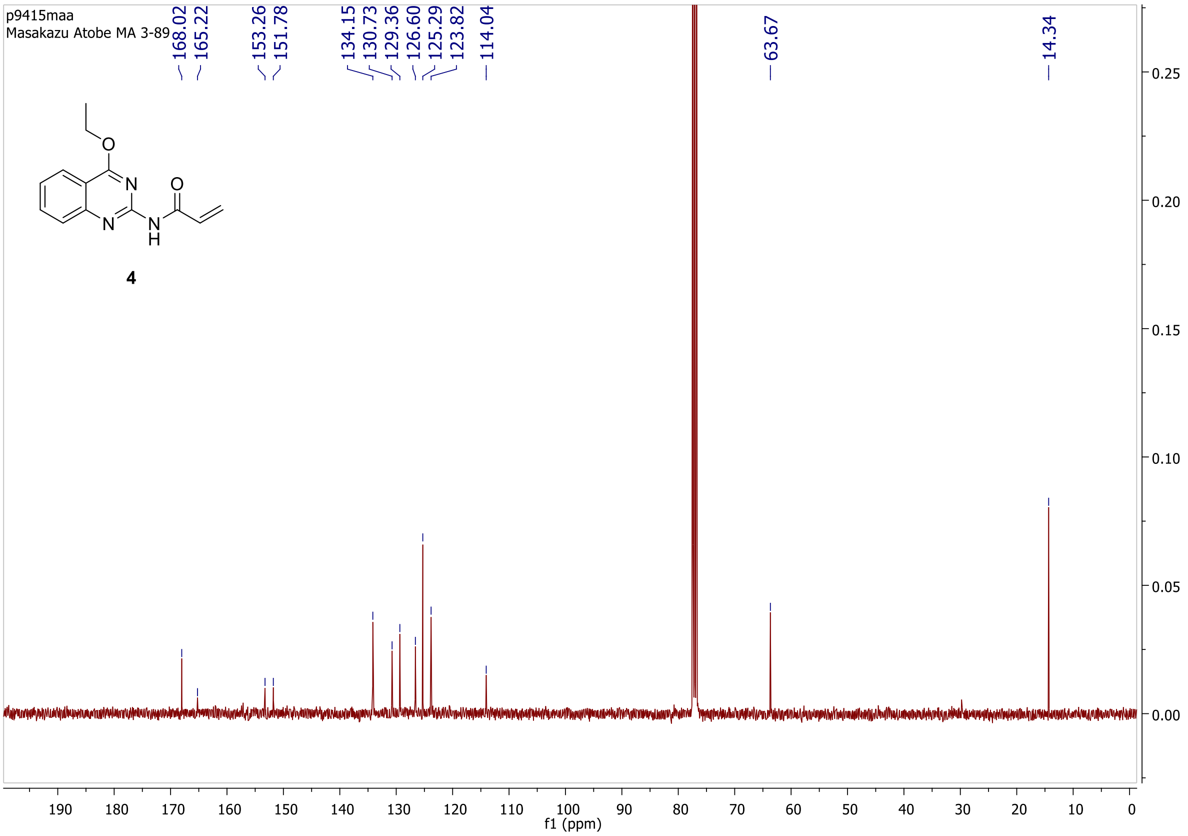

400 MHz ^{1}H NMR spectrum; 100.6 MHz ^{13}C NMR spectrum; $CDCl_3$

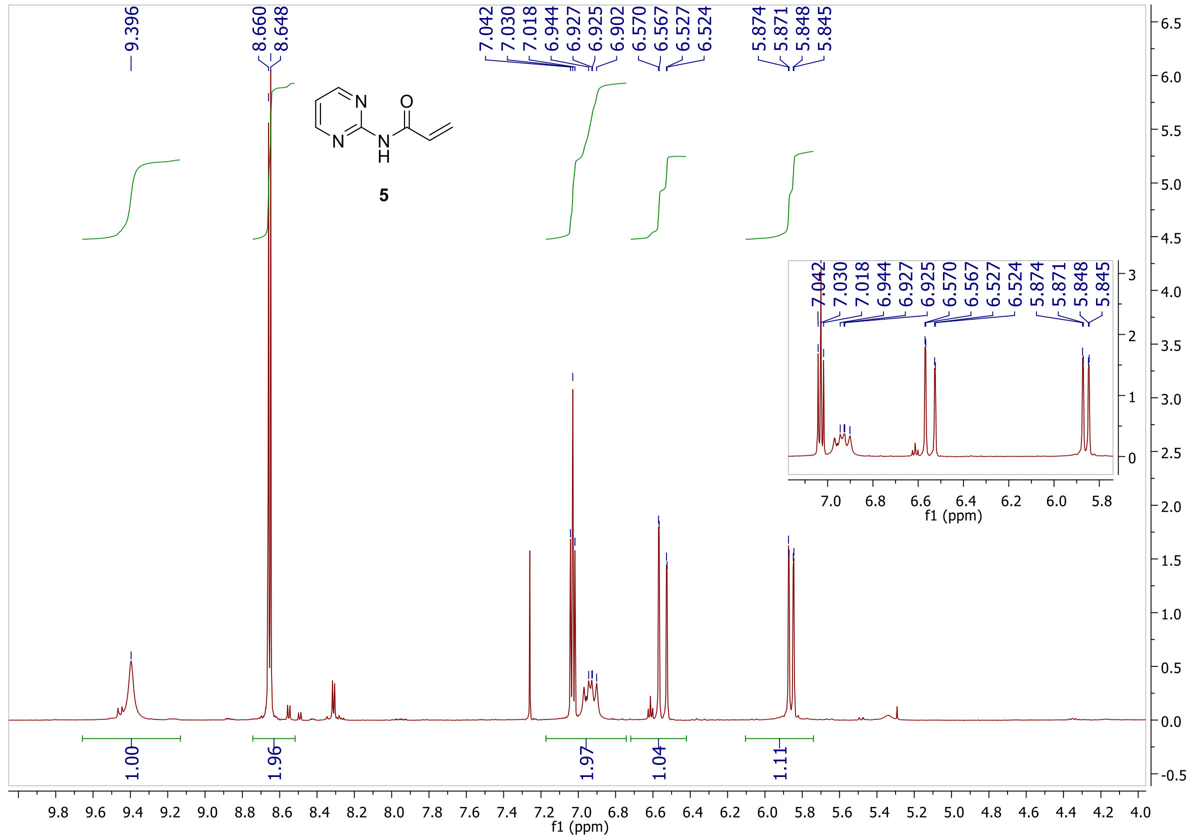

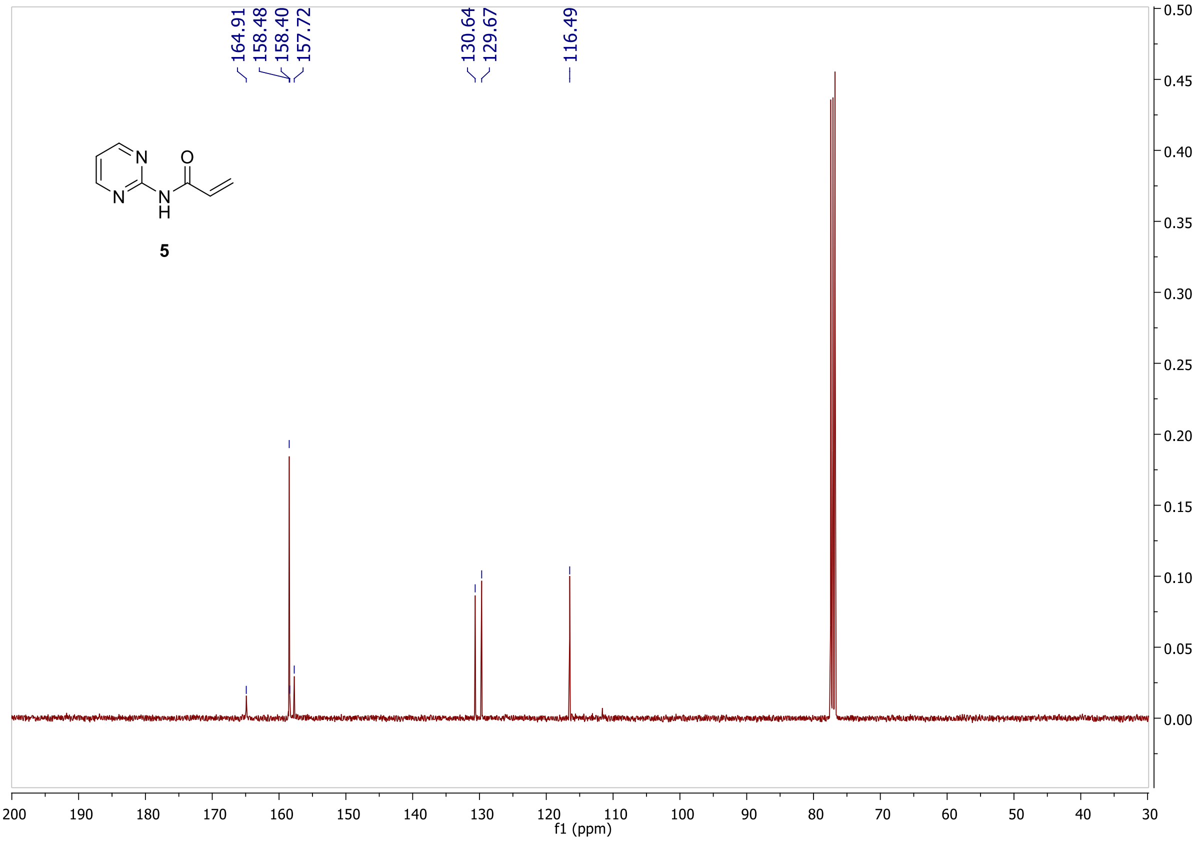

400 MHz ^{1}H NMR spectrum; 100.6 MHz ^{13}C NMR spectrum; $CDCl_3$

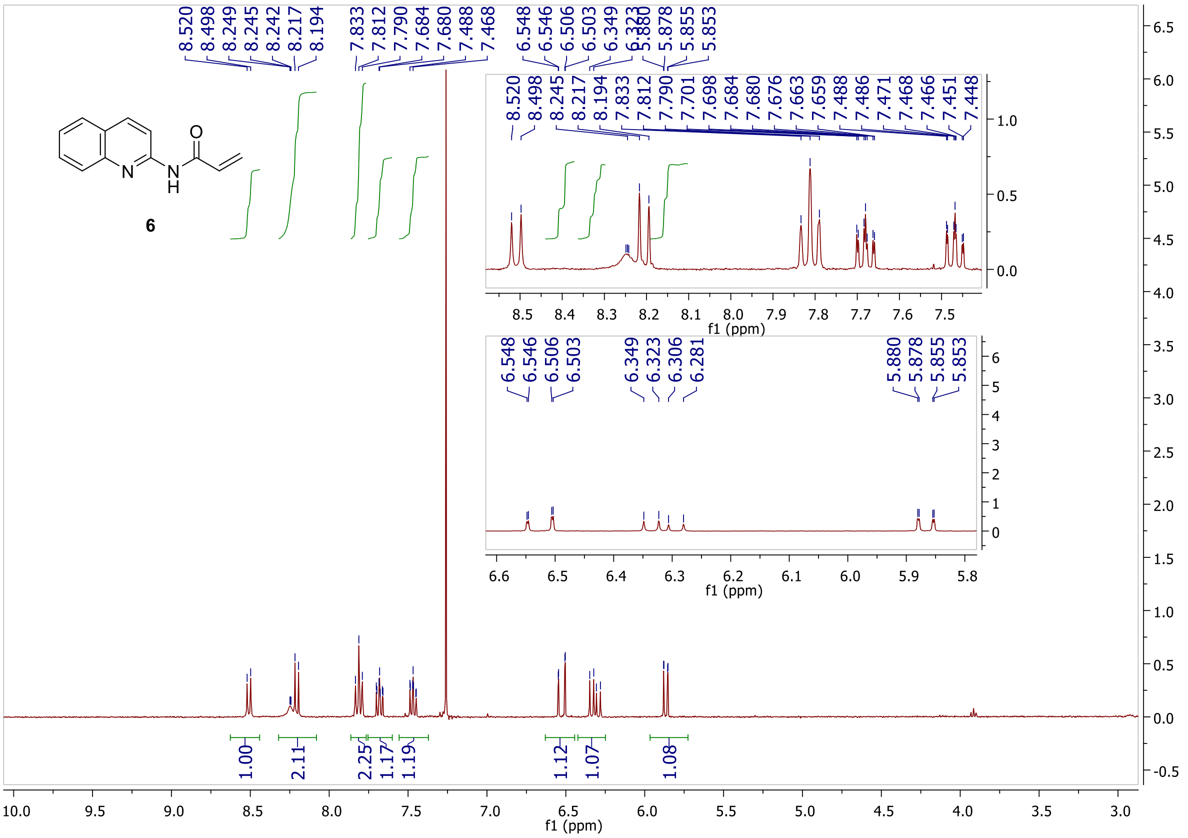

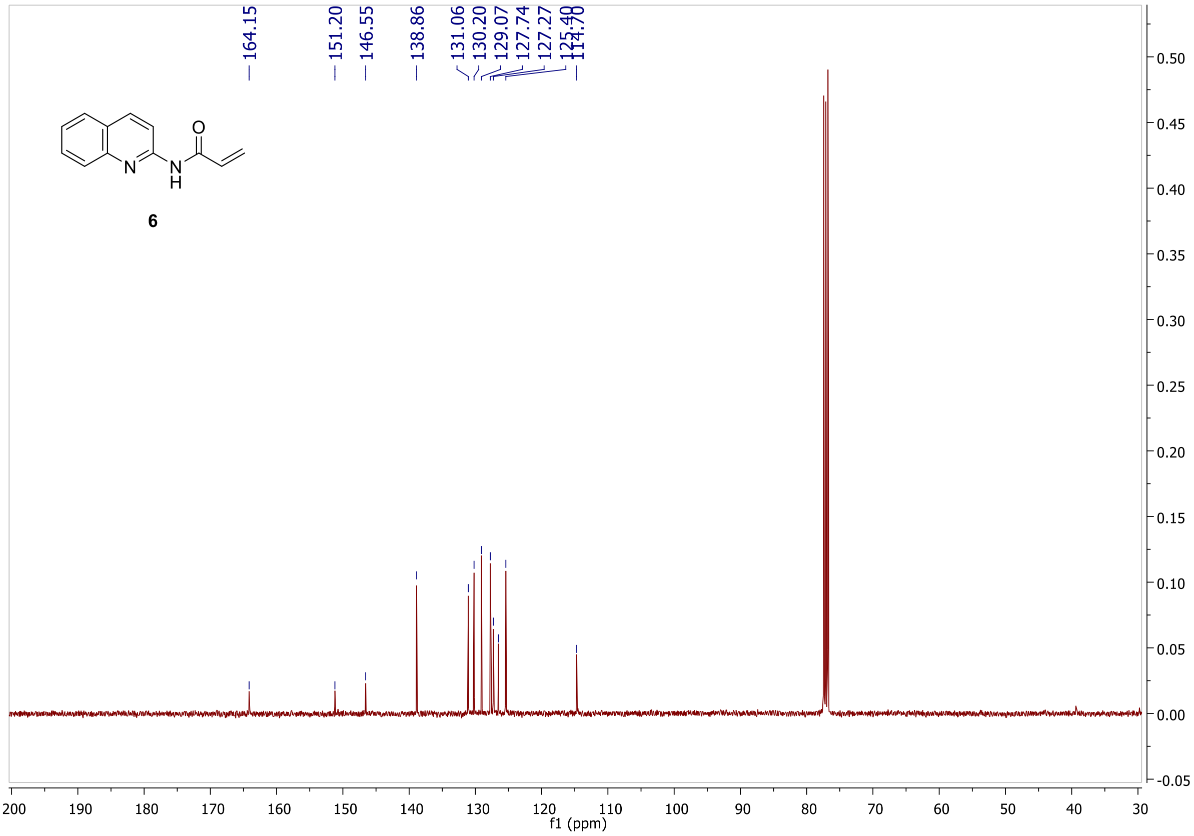

400 MHz ^{1}H NMR spectrum; 100.6 MHz ^{13}C NMR spectrum; $CDCl_3$

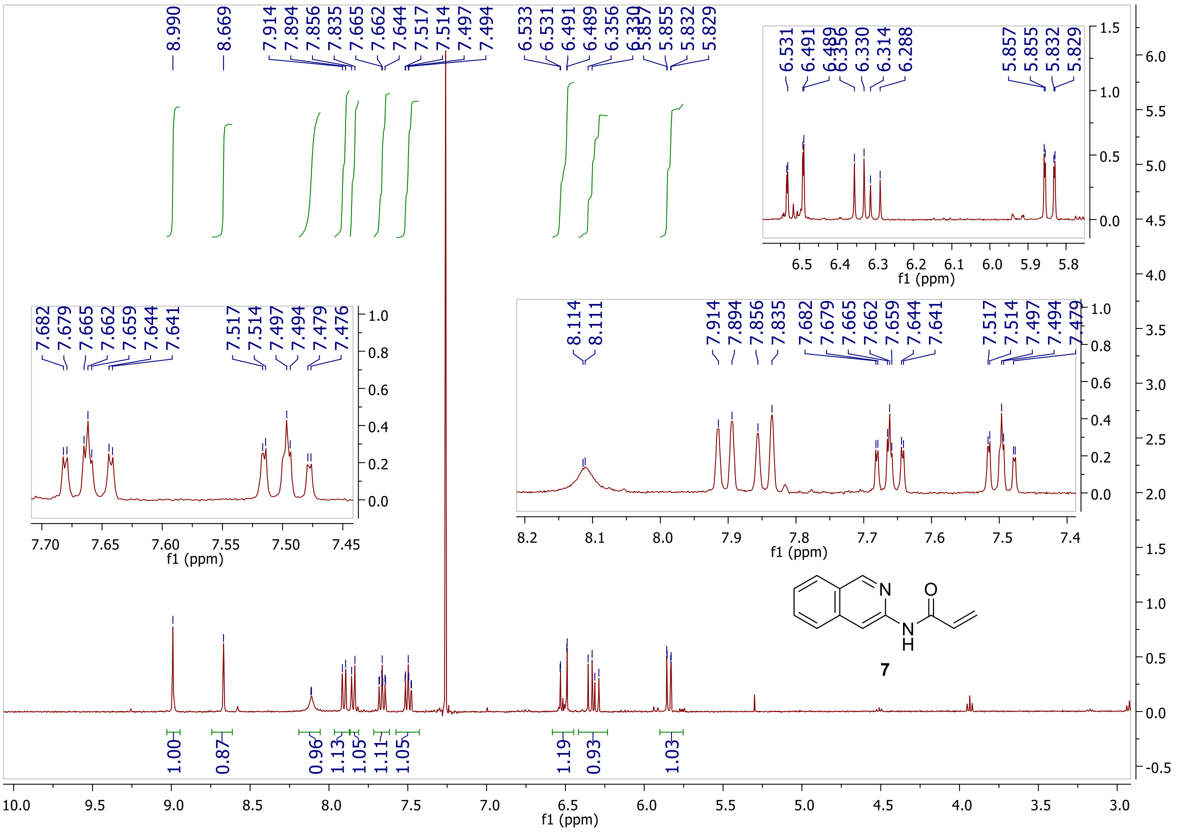

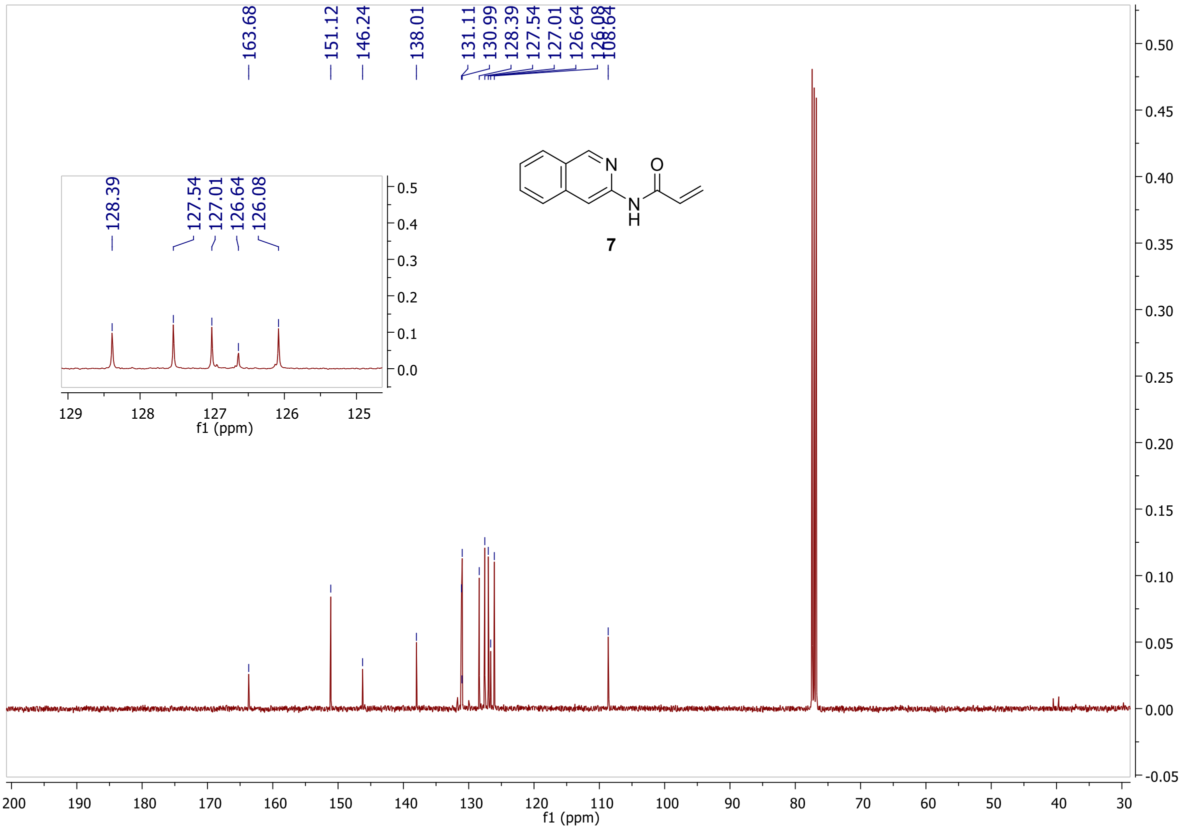

400 MHz ^{1}H NMR spectrum; 100.6 MHz ^{13}C NMR spectrum; $CDCl_3$

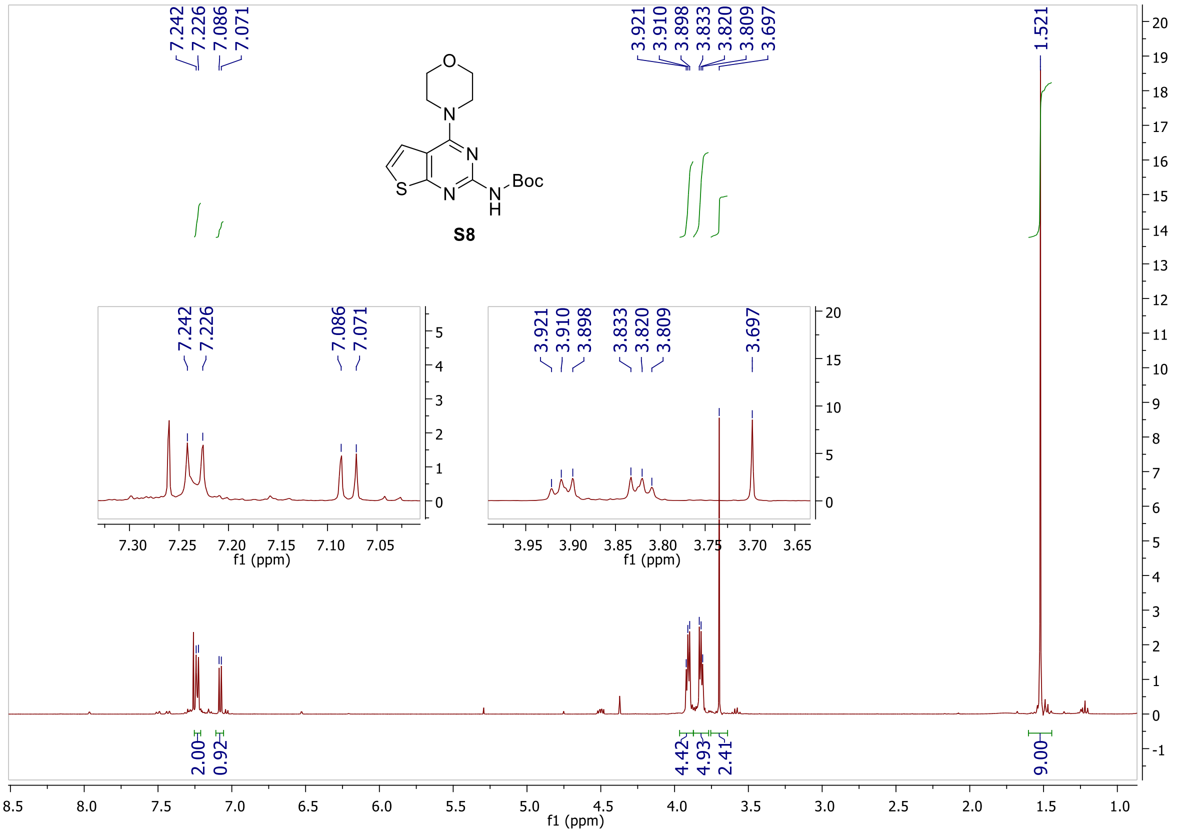

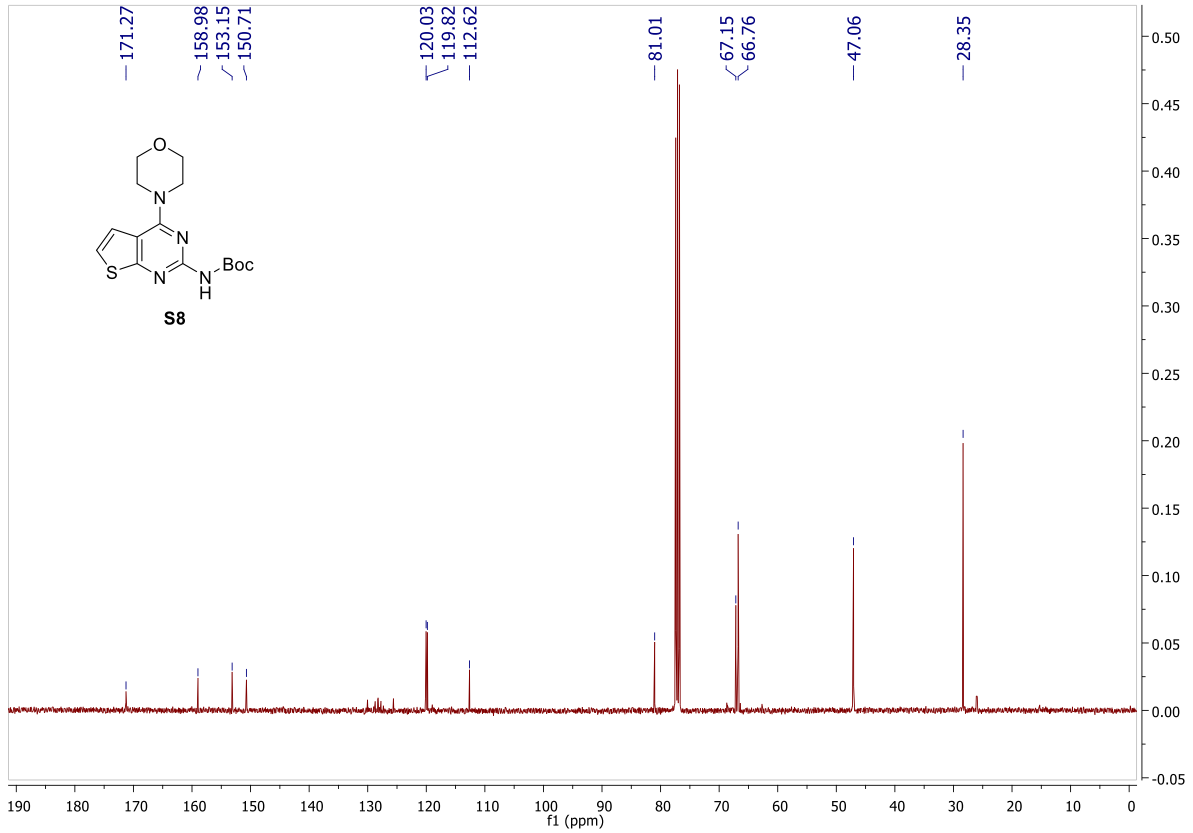

400 MHz ^{1}H NMR spectrum; 100.6 MHz ^{13}C NMR spectrum; $CDCl_3$

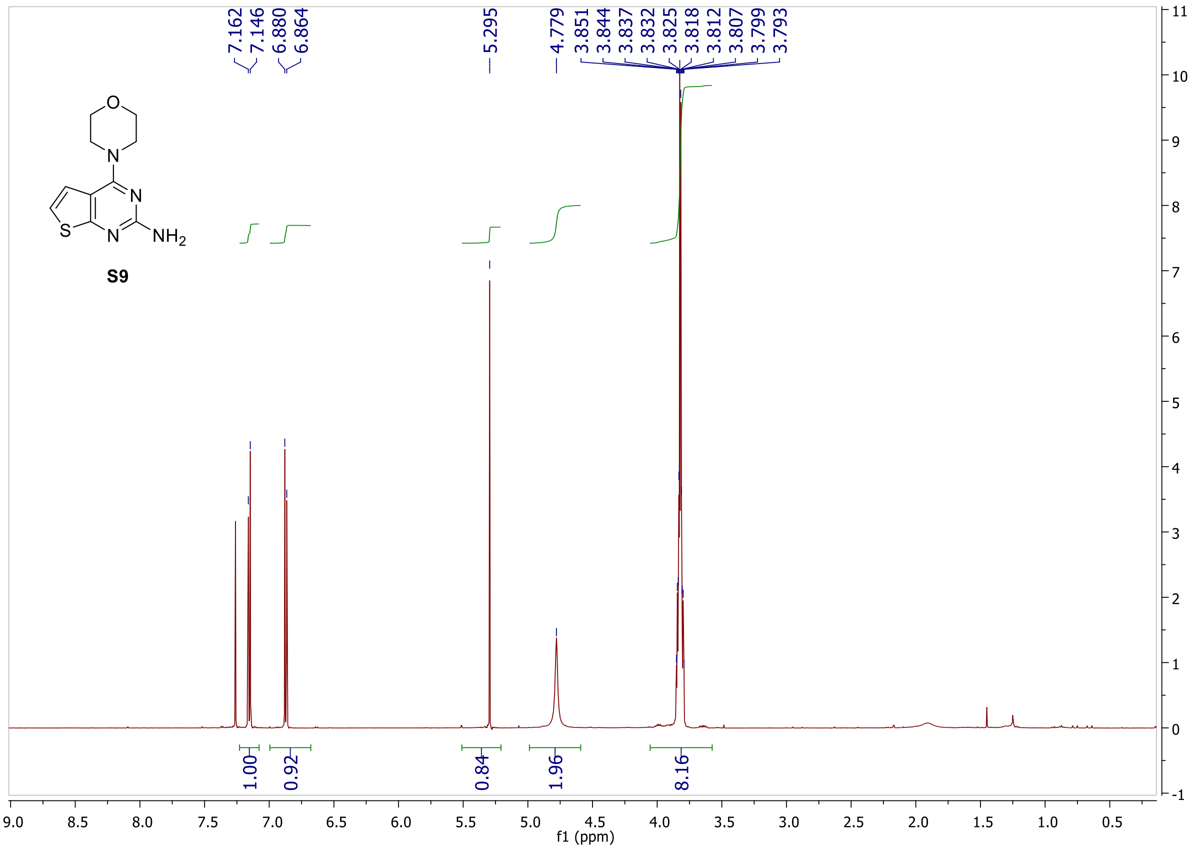

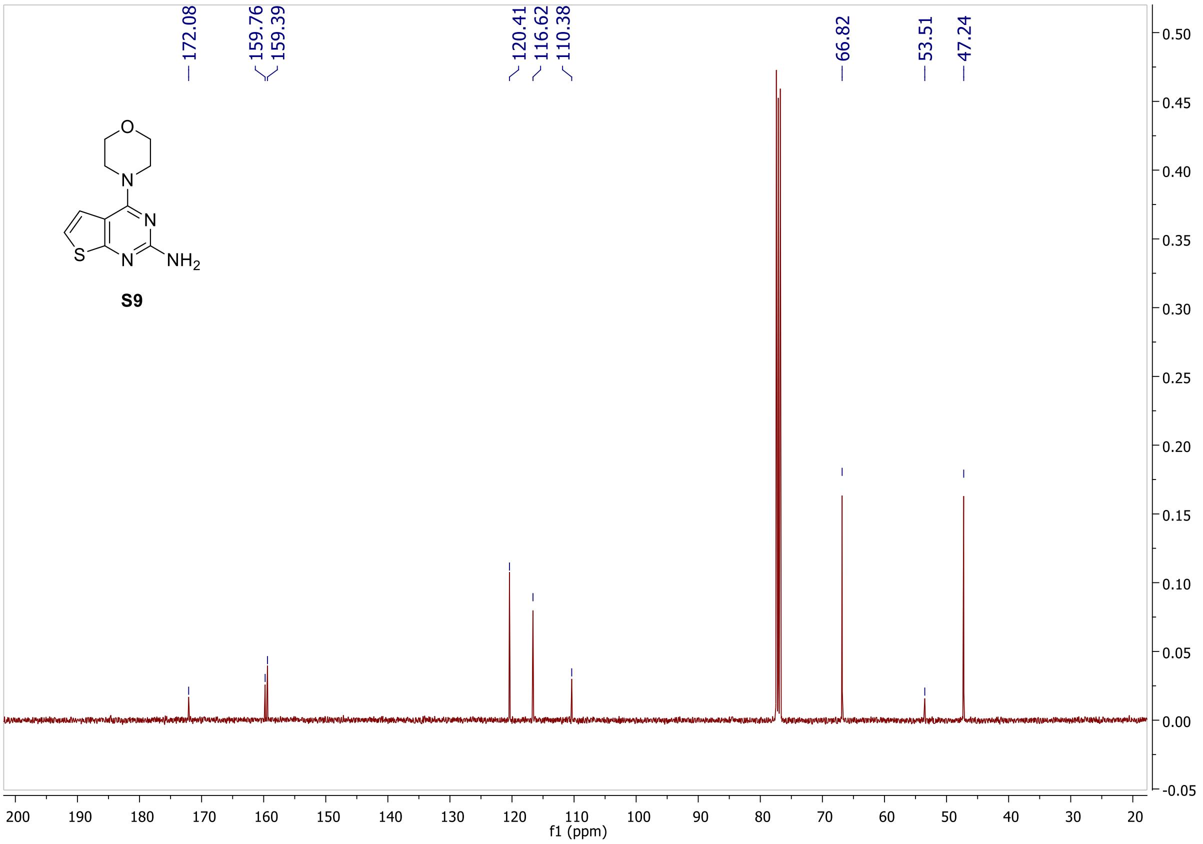

400 MHz ^{1}H NMR spectrum; 100.6 MHz ^{13}C NMR spectrum; $CDCl_3$

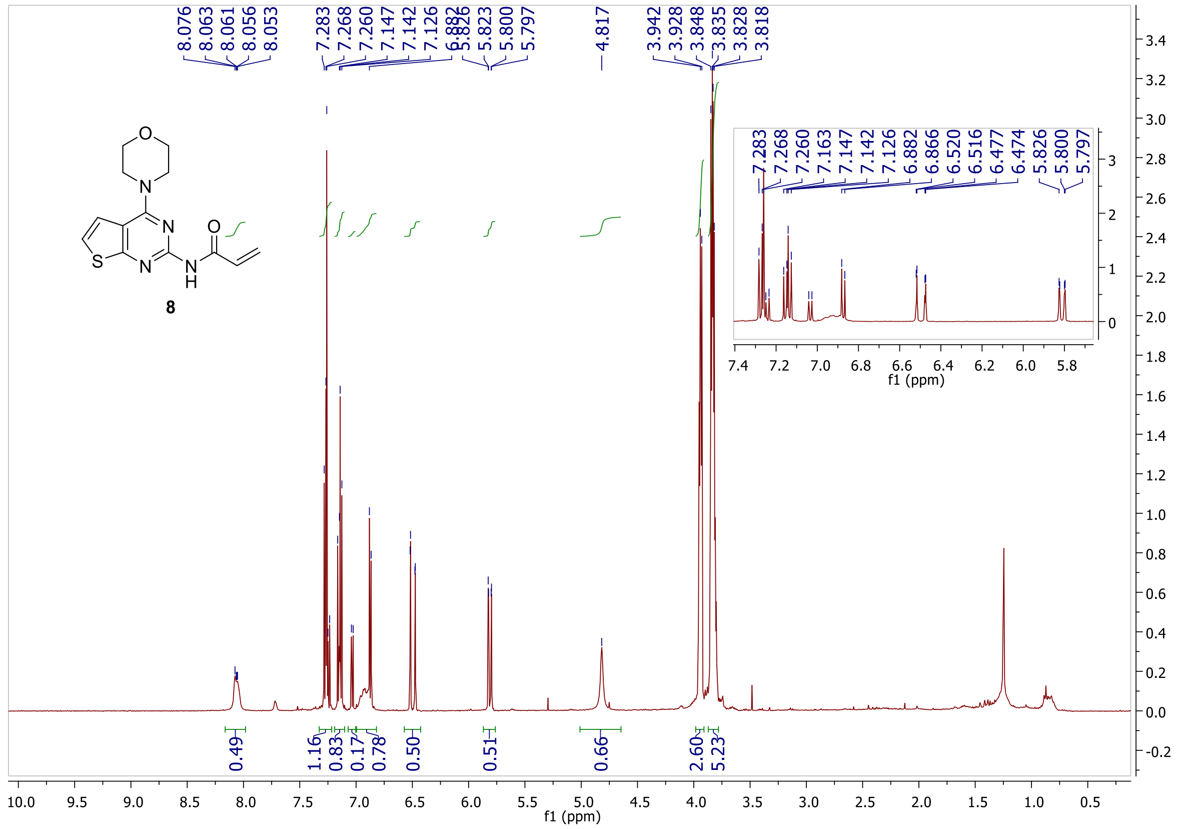

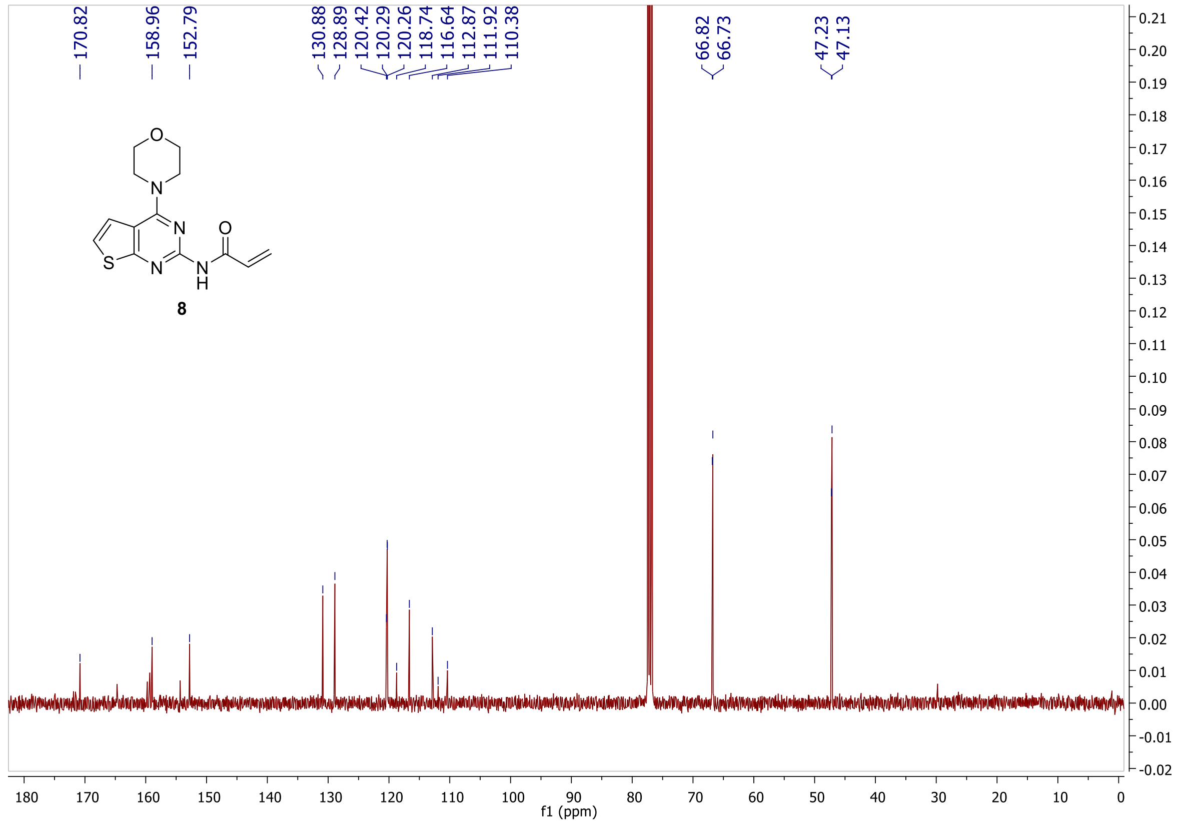

400 MHz ^{1}H NMR spectrum; 100.6 MHz ^{13}C NMR spectrum; MeOD-d_4

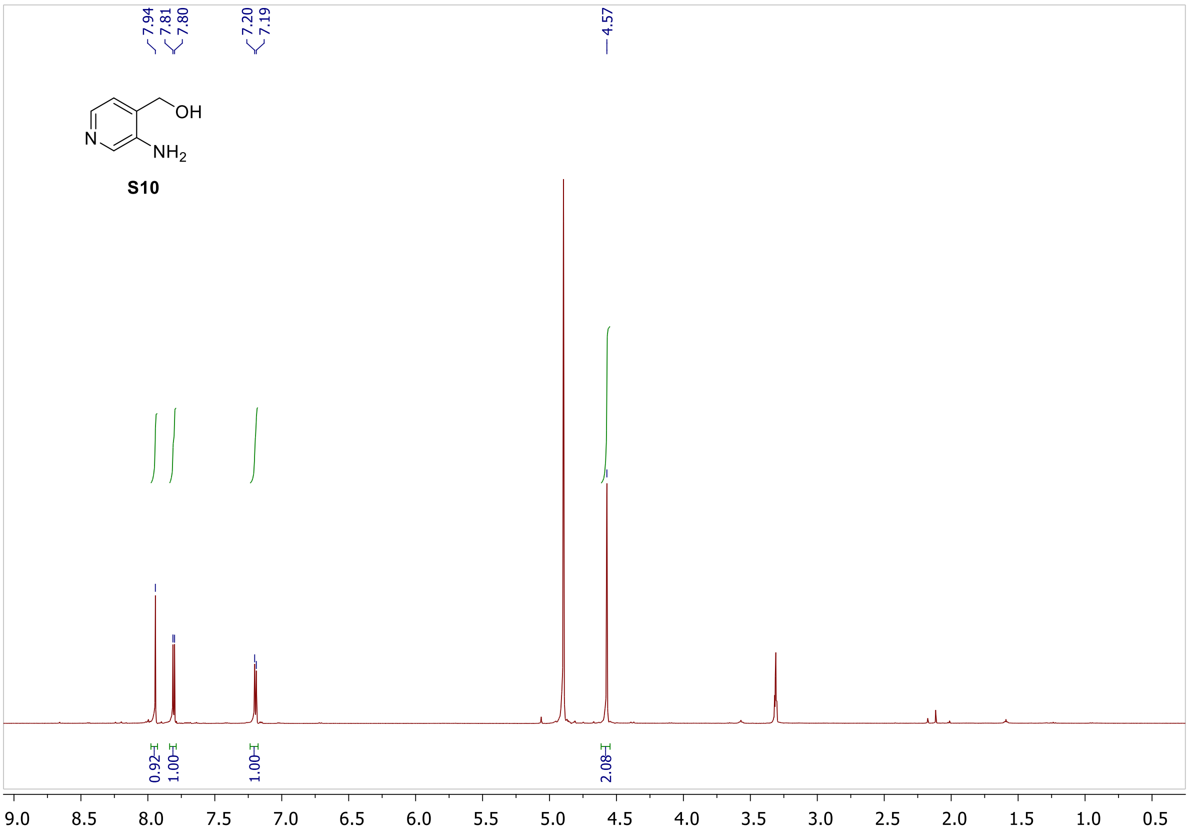

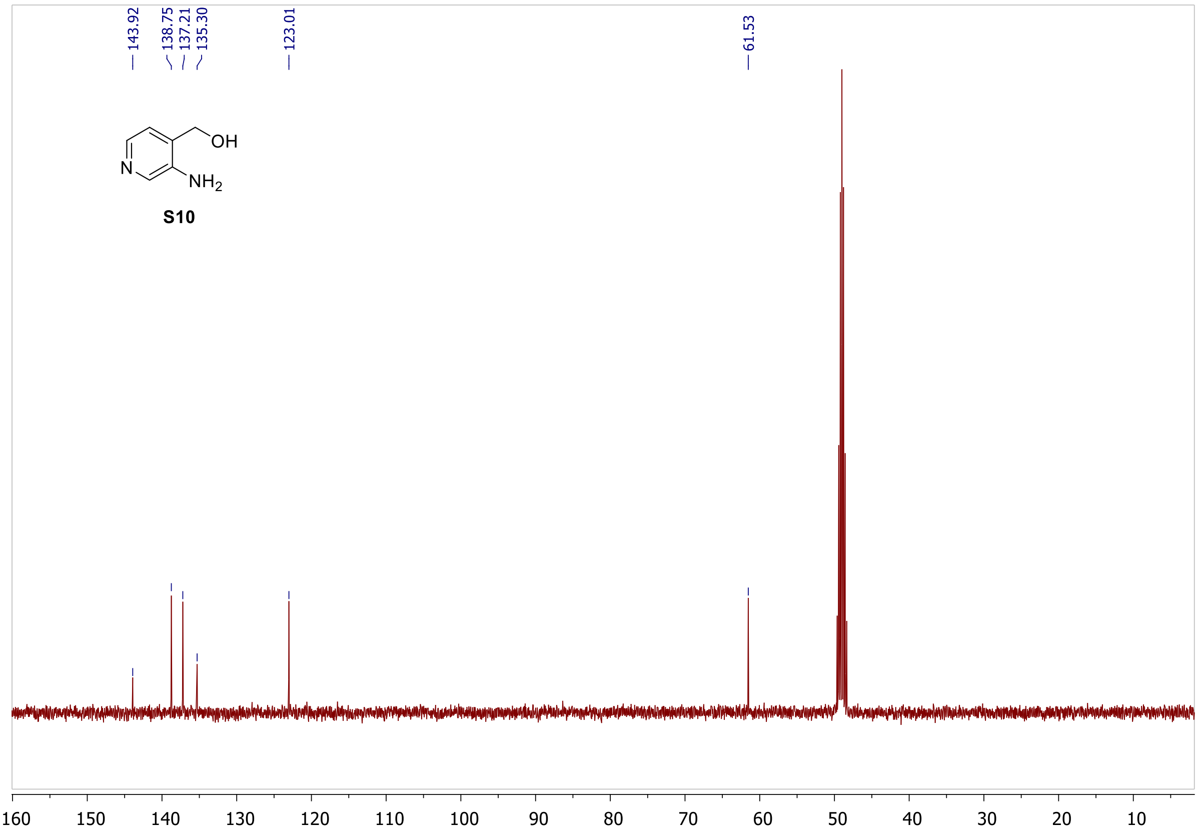

400 MHz ^{1}H NMR spectrum; $CDCl_3$

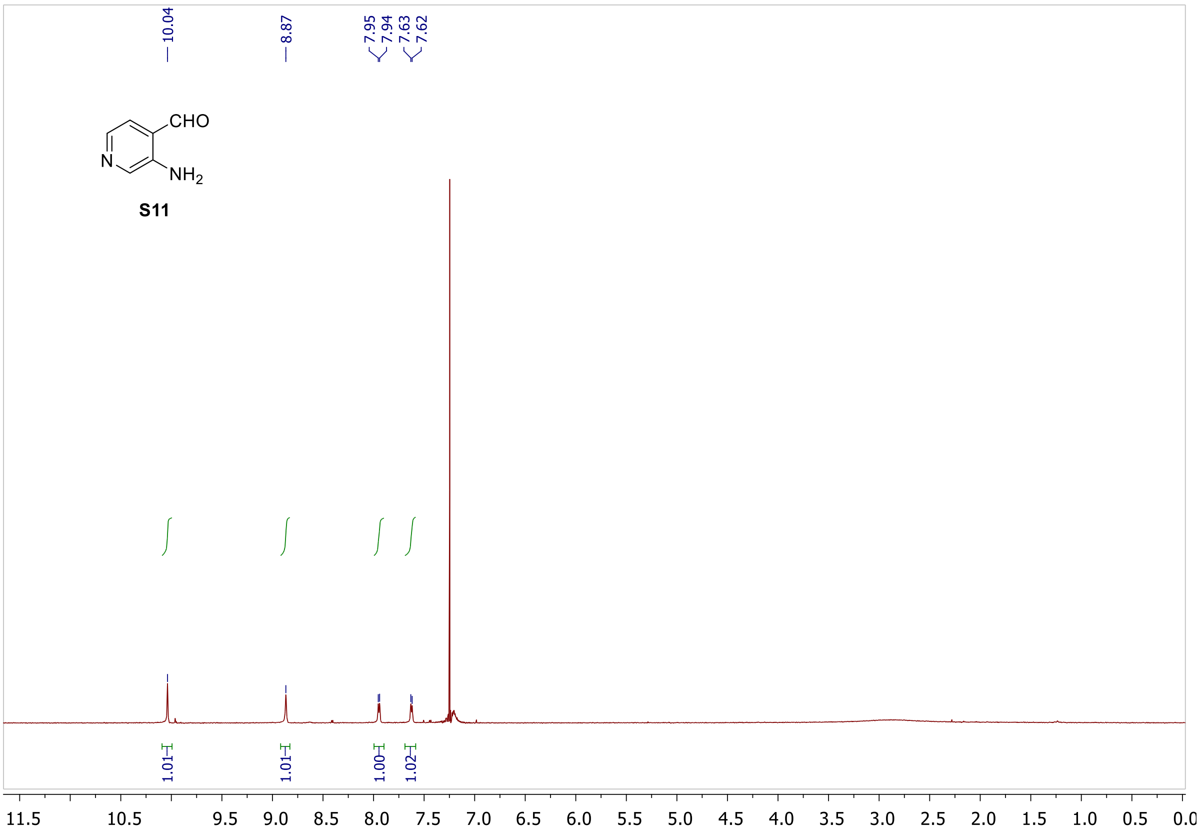

400 MHz ^{1}H NMR spectrum; MeOD-d_4

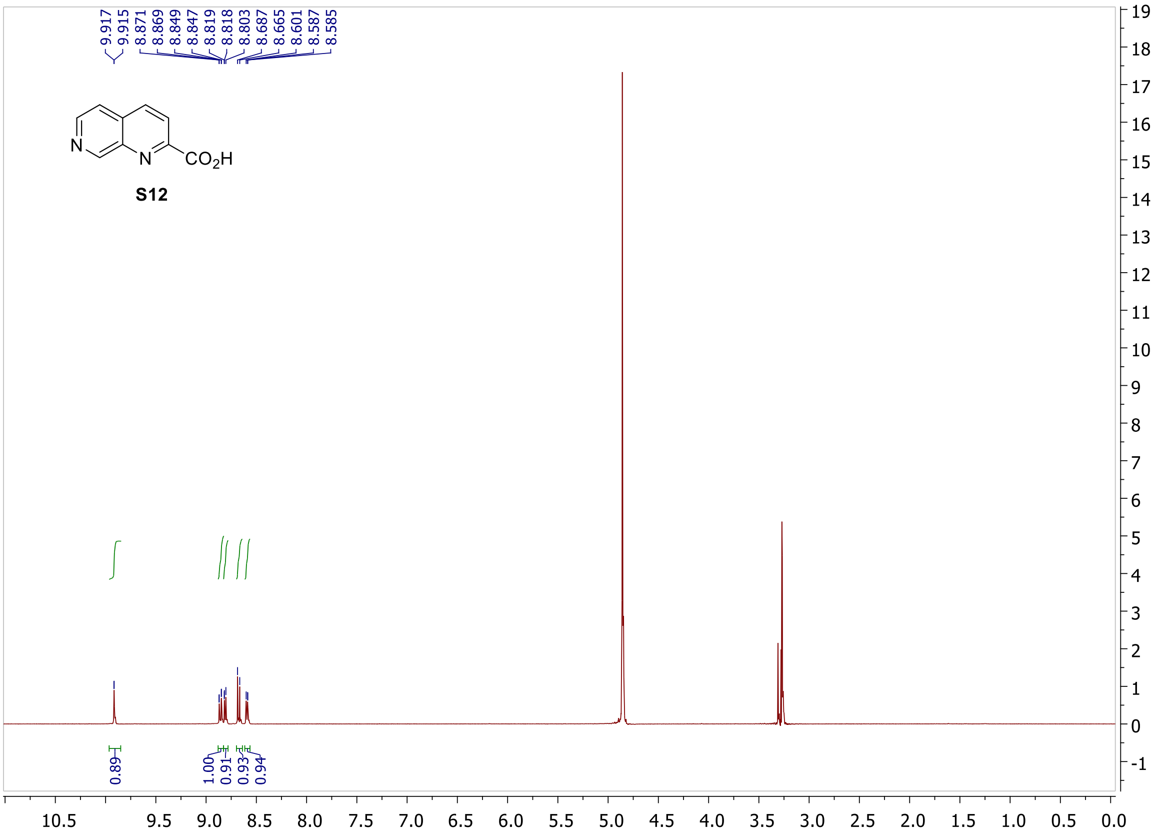

400 MHz ^{1}H NMR spectrum; 100.6 MHz ^{13}C NMR spectrum; $CDCl_3$

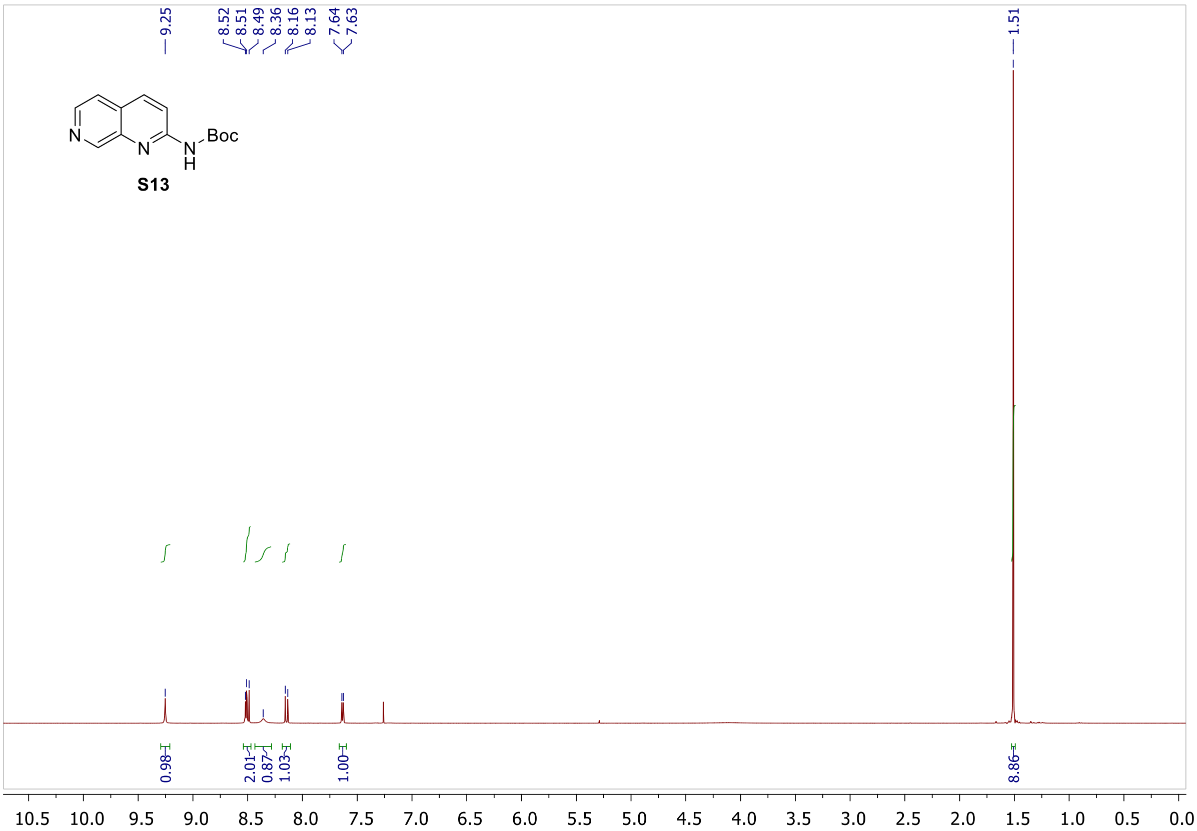

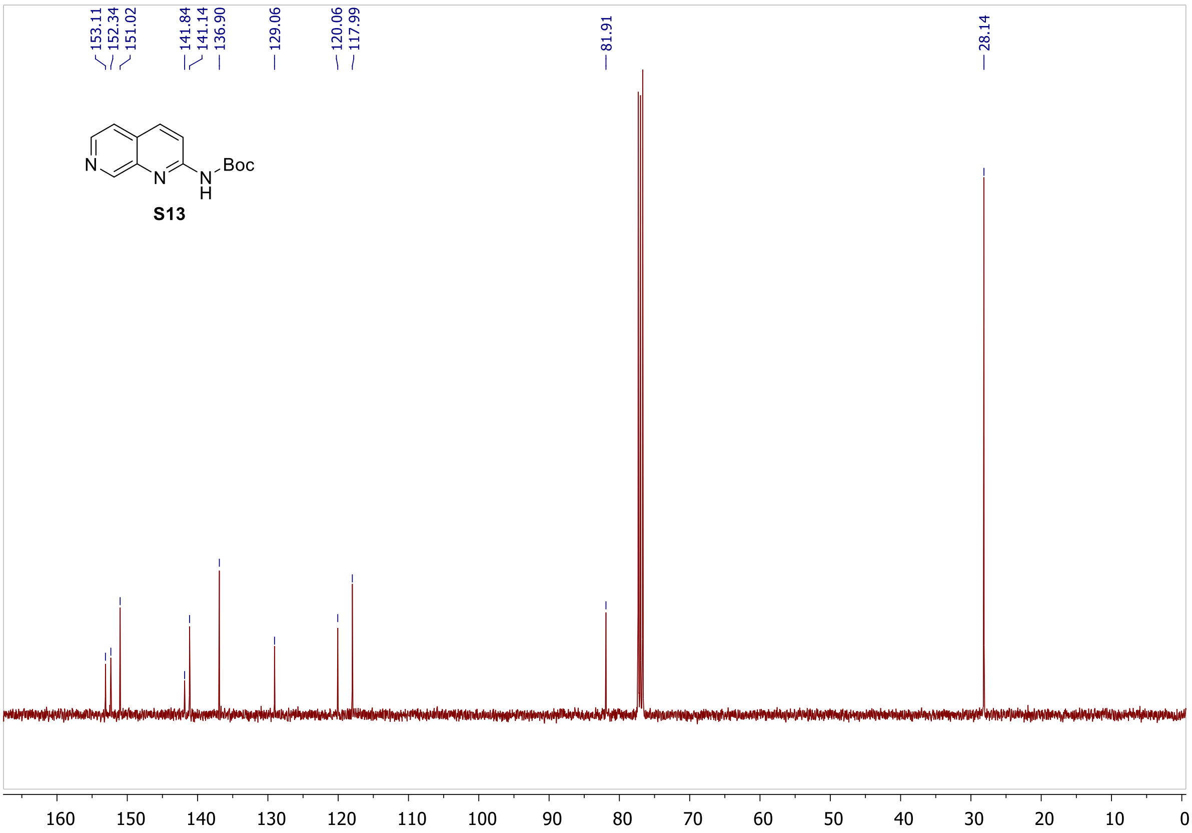

400 MHz ^{1}H NMR spectrum; 100.6 MHz ^{13}C NMR spectrum; $CDCl_3$

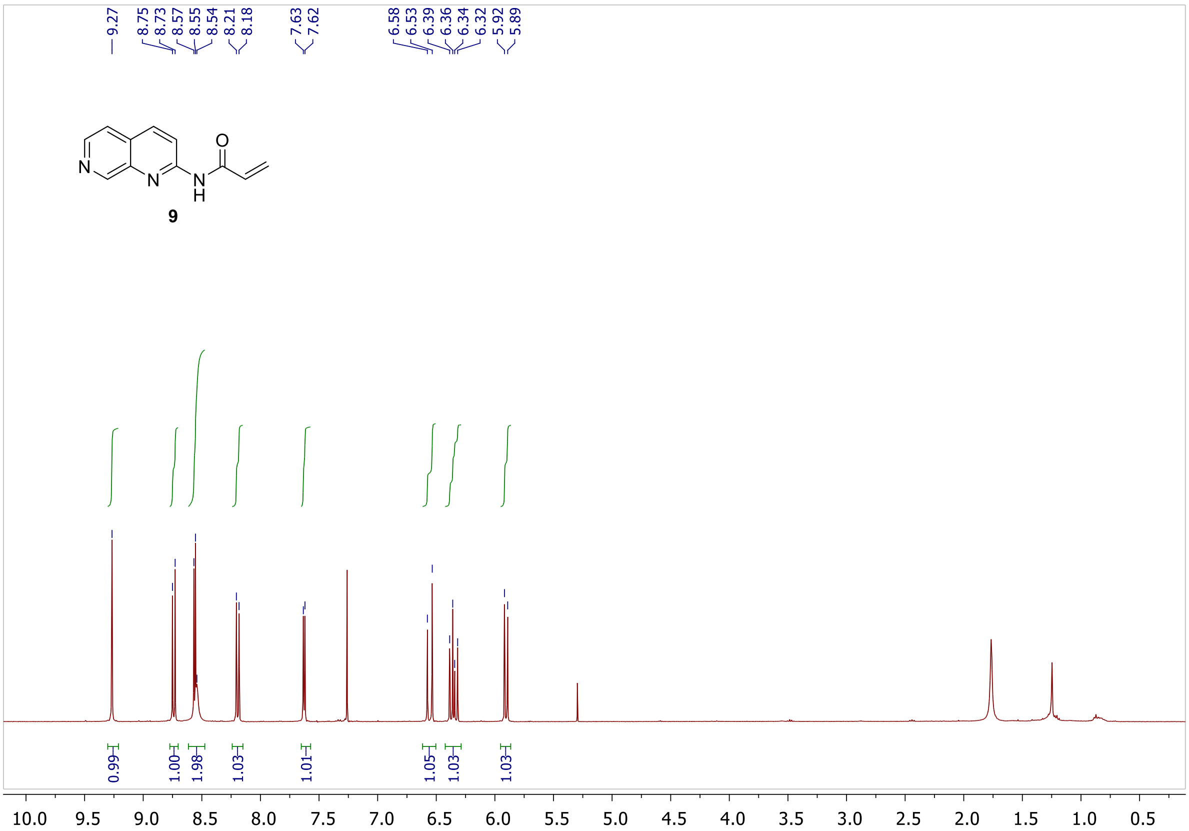

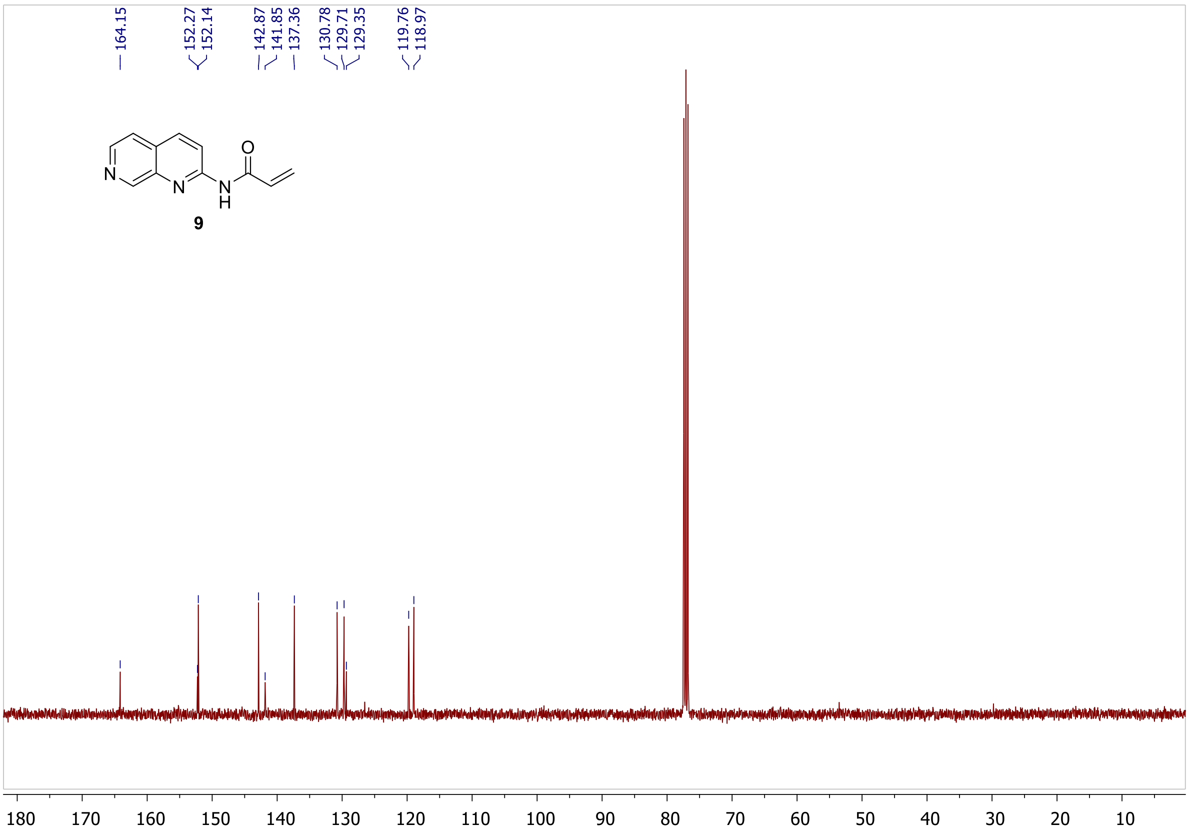

400 MHz ^{1}H NMR spectrum; 100.6 MHz ^{13}C NMR spectrum; MeOD-d_4

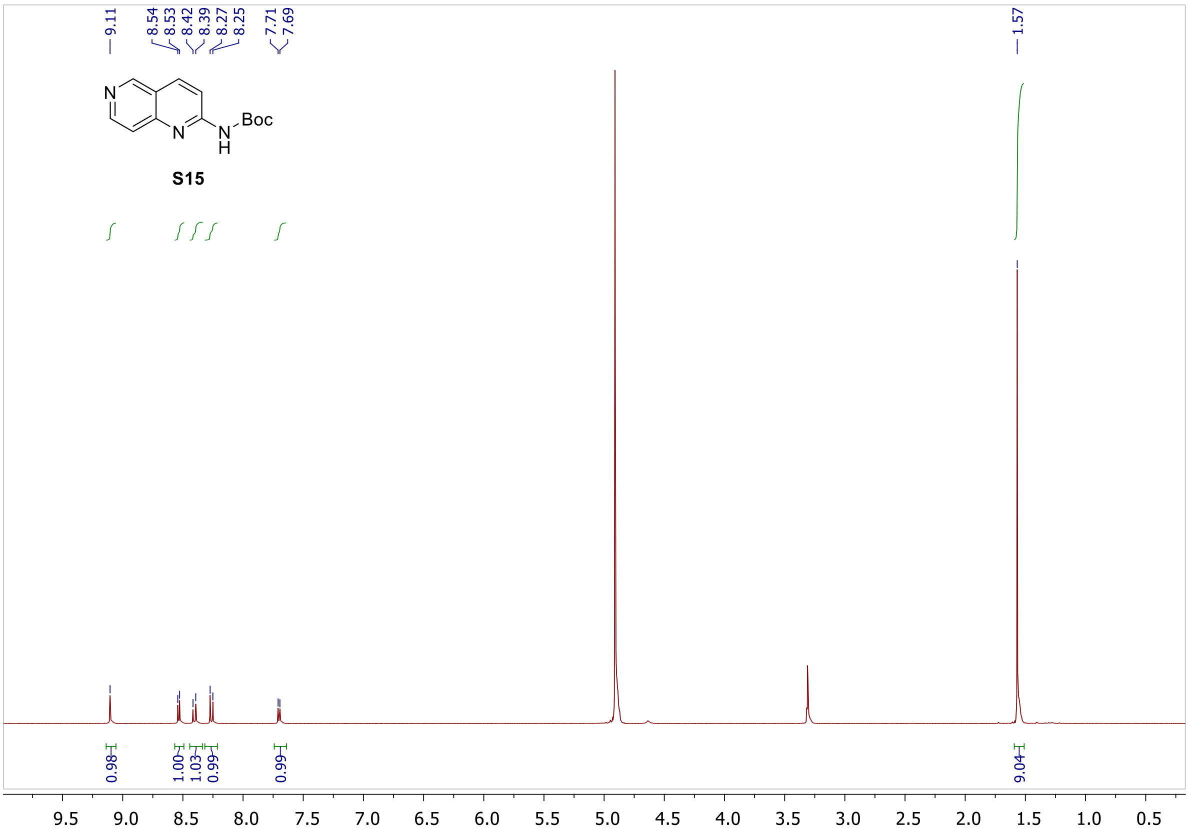

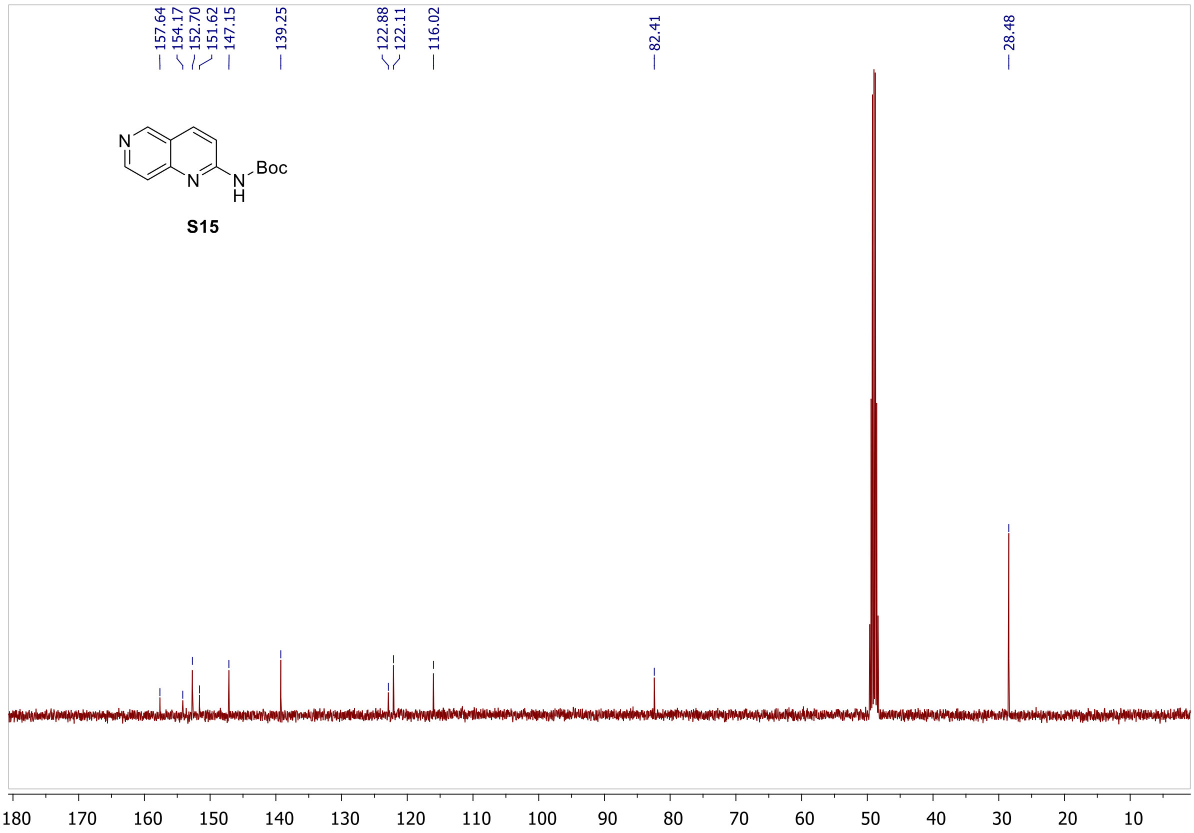

400 MHz ^{1}H NMR spectrum; 100.6 MHz ^{13}C NMR spectrum; MeOD-d_4

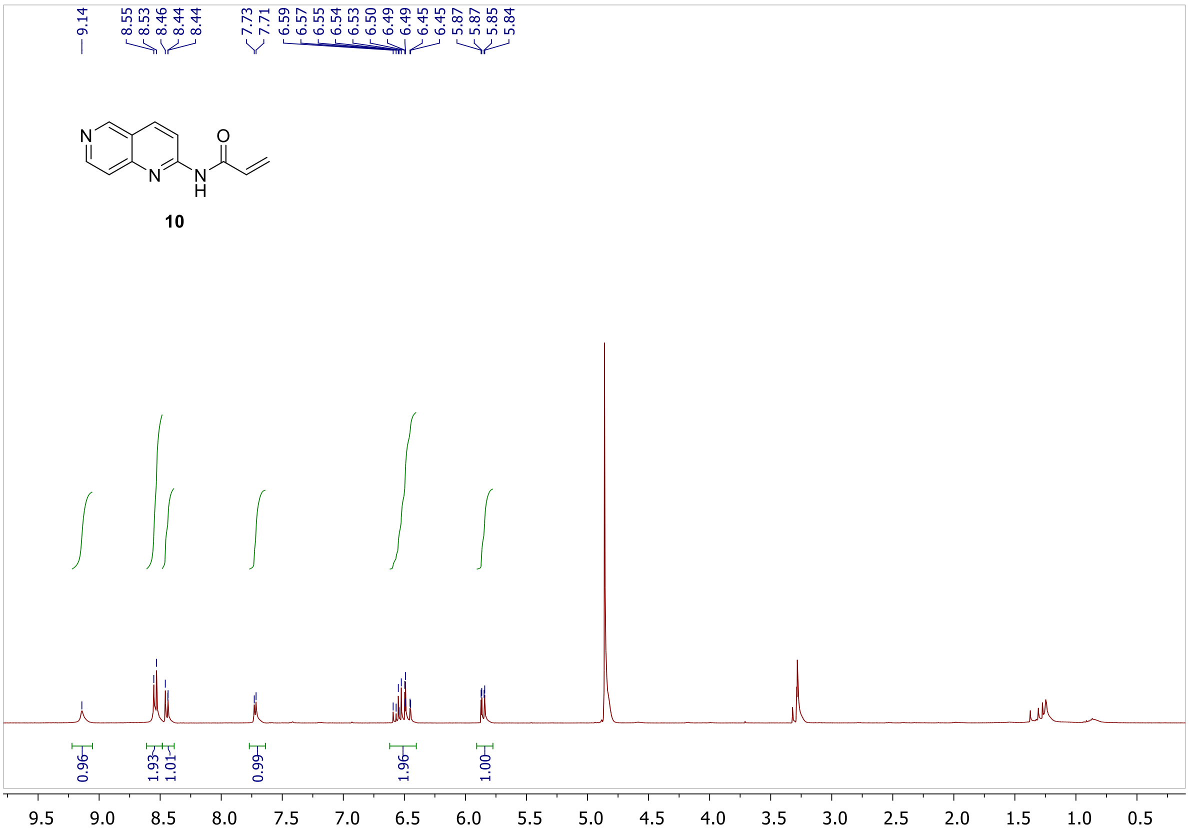

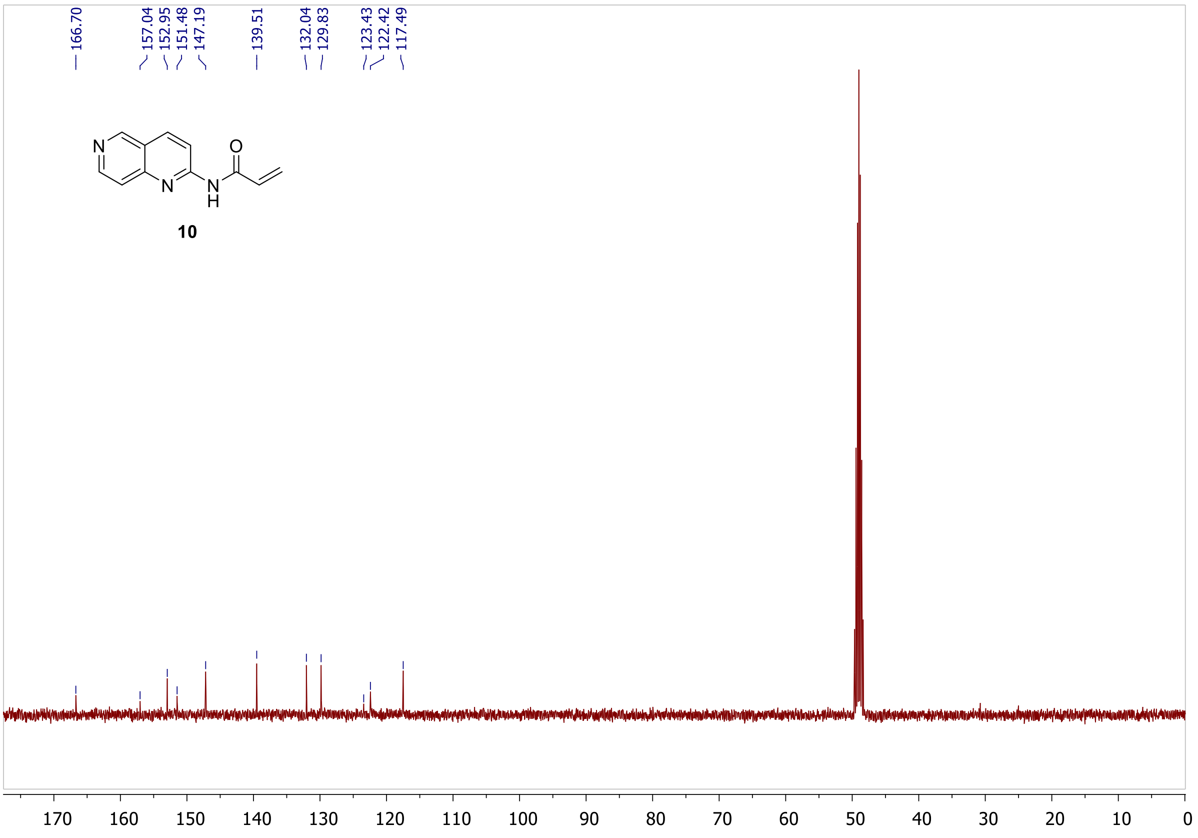

Supplement: Supplementary file 1 [file SC-008-C7SC01966A-s001.pdf]
